# The Perception of Naturalness Correlates with Low-Level Visual Features of Environmental Scenes

**DOI:** 10.1371/journal.pone.0114572

**Published:** 2014-12-22

**Authors:** Marc G. Berman, Michael C. Hout, Omid Kardan, MaryCarol R. Hunter, Grigori Yourganov, John M. Henderson, Taylor Hanayik, Hossein Karimi, John Jonides

**Affiliations:** 1 Department of Psychology, The University of Chicago, Chicago, IL, United States of America; 2 Department of Psychology, New Mexico State University, Las Cruces, NM, United States of America; 3 Department of Natural Resources and Environment, The University of Michigan, Ann Arbor, MI, United States of America; 4 Department of Psychology, The University of South Carolina, Columbia, SC, United States of America; 5 Department of Psychology, The University of Michigan, Ann Arbor, MI, United States of America; University of British Columbia, Canada

## Abstract

Previous research has shown that interacting with natural environments vs. more urban or built environments can have salubrious psychological effects, such as improvements in attention and memory. Even viewing pictures of nature vs. pictures of built environments can produce similar effects. A major question is: What is it about natural environments that produces these benefits? Problematically, there are many differing qualities between natural and urban environments, making it difficult to narrow down the dimensions of nature that may lead to these benefits. In this study, we set out to uncover visual features that related to individuals' perceptions of naturalness in images. We quantified naturalness in two ways: first, implicitly using a multidimensional scaling analysis and second, explicitly with direct naturalness ratings. Features that seemed most related to perceptions of naturalness were related to the density of contrast changes in the scene, the density of straight lines in the scene, the average color saturation in the scene and the average hue diversity in the scene. We then trained a machine-learning algorithm to predict whether a scene was perceived as being natural or not based on these low-level visual features and we could do so with 81% accuracy. As such we were able to reliably predict subjective perceptions of naturalness with objective low-level visual features. Our results can be used in future studies to determine if these features, which are related to naturalness, may also lead to the benefits attained from interacting with nature.

## Introduction

Research has demonstrated that interacting with natural environments can have beneficial effects on memory and attention for healthy individuals [1]–[3] and for patient populations [4]–[6]. In addition, views of natural settings have been found to reduce crime and aggression [7], [8] and also improve recovery from surgery [9].

All of this evidence points to the importance of interacting with natural environments to promote mental and physical health. Yet, it is not clear exactly what it is about natural environments compared to urban or built environments that leads to these benefits. Problematically, there are numerous dimensions that differentiate natural from urban environments, so uncovering the most salient features that define natural environments would seem important given that there is something about natural environments that leads to salubrious effects for both cognitive and affective processing.

While there are a number of theories that posit why nature is restorative [2], [10]–[14], it would be difficult to use these theories to inform the design of green spaces because these theories tend not to outline in a prescriptive way how to design a natural space to obtain the most benefit. In his seminal 1995 paper, Kaplan does list some criteria that would appear to be important for a natural environment being restorative: the environment must have sufficient extent, the environment must be compatible with one's goals, the environment must give people the sense of being away, and the environment must be fascinating[10]. For the most part, it is currently not known how some of these concepts could be used to design a greenspace in a way to optimize psychological functioning.

The purpose of this research is to define low-level visual features that define objective and subjective measures of naturalness. We are not the first to examine how objective measures may characterize classes of natural and urban scenes as this has been done with great success and sophistication in the context of computer vision [15]–[19], and mammalian vision [20]–[23]. However, the purpose of those studies was to classify/categorize scene types or to relate the biology of primary vision to statistical regularities of natural scenes. Here our purpose was not the classification of scenes, but rather identifying simple, low-level visual features that related to subjective perceptions of naturalness and could be readily manipulated in visual stimuli. Future research could then use such features, which can be easily manipulated, to test and design new environments in ways that may improve psychological functioning.

We accomplished this in three experiments. In the first experiment we had participants rate the similarity of images of parks that had varied natural and built content. Afterwards we examined these similarity data using a multidimensional scaling analysis (MDS; [24], [25]). This technique was used to identify the underlying featural dimensions that participants relied on when making their similarity estimates (similar procedures have been utilized by Ward and colleagues: [26]–[28]. To obtain explicit labels for the uncovered dimensions from MDS (the makeup of MDS dimensions must be inferred from the organization of the space), we conducted a second experiment in which naïve participants examined the MDS output, and labeled the dimensions according to their subjective impression of how the space was organized.

The most common label for the first dimension, i.e., the dimension that explained the most variance in similarity, was naturalness. Importantly, these dimension weights correlated strongly with direct measures of naturalness on each of the images as determined by a second group of independent raters (Experiment 3). Finally, we quantified low-level visual features for all of our images and were able to relate some of these low-level visual features to direct measures of naturalness and also to the first MDS dimension that represented a latent measure of naturalness.

Importantly, it has not been determined the degree of correspondence between subjective and objective measures of ‘naturalness’ and this study addresses this point head on. Our results show a strong correspondence. Another equally important point is that by uncovering the features that are most related to perceived naturalness, or defining what a natural environment is, these features may be found to be causal in producing the positive effects of interacting with natural environments. The features that define perceived naturalness could then be manipulated in future work to determine how they impact the restorativeness of natural environments.

## Experiment 1: Spatial Multidimensional Scaling (MDS)

### Materials and Methods

#### Participants

Twenty participants from the University of Michigan took part in this study (mean age = 19.8; # female = 20). This research was approved by the Institutional Review Board of the University of Michigan (IRB #HUM00006681). All participants provided written informed consent as administered by the Institutional Review Board of the University of Michigan (IRB# HUM00006681). Participants were compensated $10/hour for their participation.

#### Materials

The stimuli used in this study were photographs (.BMP format) taken from parks built by the TKF Foundation, a private foundation based in Annapolis, MD. The parks were from a range of locations around the Baltimore, Washington D.C. and Annapolis area. The photographs were resized to a maximum of 200 pixels along either dimension, maintaining the original aspect ratio, and assuring that the images were large enough so that the visual information in the images was easily detectable. They were shown on monitor that was 41 cm×30 cm, at a resolution of 1280×1024.

#### Procedure

In order to assess the dimensions that characterized the TKF sites we composed a paradigm to compare images from 70 different TKF sites. We performed this experiment twice using different images of each site for each iteration of the experiment. We did so to ensure that our results were not idiosyncratic to the particular pictures that were selected; 62 sites had multiple pictures, but the other sites did not. Therefore, we used pictures from 16 additional TKF sites that only had single images (8 for each set). One set of images we labeled set 1 and the other set of images was labeled set 2.

On each trial, fifteen different pictures (pulled from a set of seventy total pictures) were shown to the participant, arranged in 3 discrete rows, with random item placement. Fifteen images was the largest set of images that could be displayed simultaneously to the participants without overcrowding the display. Participants were instructed to drag and drop the images in order to organize the space such that the distance among items was proportional to each pair's similarity (with closer in space denoting greater similarity). Participants were given as much time as they needed to scale each set; typically, trials lasted between 2 and 5 minutes. The x- and y-coordinates of each image was then recorded and the Euclidean distance between each pair of stimuli was calculated (for 15 stimuli there are 105 pairwise Euclidean distances). This procedure was performed repeatedly (over 29 trials), but with different image sets on each trial, so that all pairwise comparisons among the 70 total images were recorded. Thus, this provided a full similarity matrix comparing the ratings of each image to all of the other images (i.e., all 2415 comparisons) for each participant. This took participants about an hour to complete; similar rating procedures have been used by other researchers [29]–[31].

We controlled the selection of images on each trial by employing a Steiner System [32]; these are mathematical tools that can be used to ensure that each item in a pool is paired with every other item (across subsets/trials) at least once. A Steiner System is denoted S(*v, k, t*), where “v” is the total number of stimuli, “k” is the number of items in each subset, and “t” is the number of items that need to occur together. Thus for us, *v, k*, and *t*, are 70 (total images), 15 (images per trial), and 2 (denoting pairwise comparisons), respectively. Simply put, the Steiner System provides a list of subsets (i.e., trials) identifying which items should be presented together on each trial. For some combinations of *v* and *k*, there may exist a Steiner set the does not repeat pairwise comparisons (i.e., each pair of items is shown together once and only once). For other combinations (including ours), some stimuli must be shown with others more than once. Because this leads to multiple observations per “cell”, we simply took the average of the ratings for the pairs that were shown together more than once. Across participants, images were randomly assigned to numerical identifiers in the Steiner System, which ensured that each participant saw each pair of images together at least once, but that different people received different redundant pairings.

#### MDS Analysis

After the similarity matrices were composed, we performed multidimensional scaling [25] on the pairwise Euclidean distances using PROXSCAL [33], implemented in SPSS. PROXSCAL allows for both metric and non-metric MDS and we chose the metric version since pixel distances should be equivalent across the screen (i.e., the distance between 10 and 15 pixels should be psychologically the same as the distance between 30 and 35 pixels). We did not rotate the spaces because the group plots in MDS utilize orthogonal dimensions.

**Figure 1:**
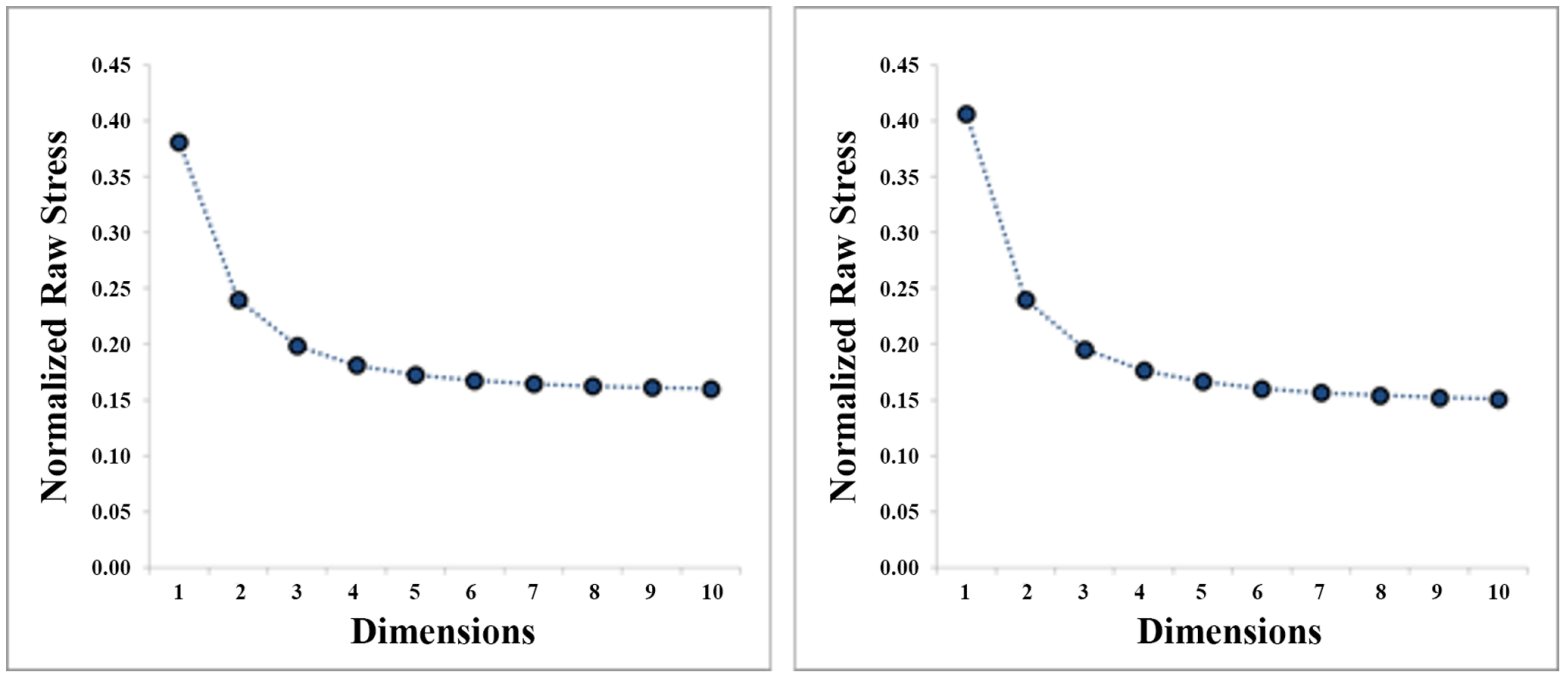
Scree plots for the *First set* (left) and the *Second set* (right) of images. Stress values are plotted as a function of the dimensionality in which the MDS data were scaled.

To determine the appropriate dimensionality for our data, we created Scree plots for each MDS space, plotting the model's stress against the number of dimensions used in the space. Stress functions vary across scaling algorithms (PROXSCAL uses “normalized raw stress”), but all are designed to measure the agreement between the estimated distances provided by the MDS output and the raw input proximities themselves (lower stress values indicate a better model fit). Scree plots are often used to determine the ideal dimensionality of the data by identifying the point at which added dimensions fail to improve the model fit substantially [34]. For all four datasets, we found that stress levels plateaued at 4 dimensions (see Fig. 1); thus, the data were analyzed in 4 dimensions.

### Experiment 1 Results

**Figure 2:**
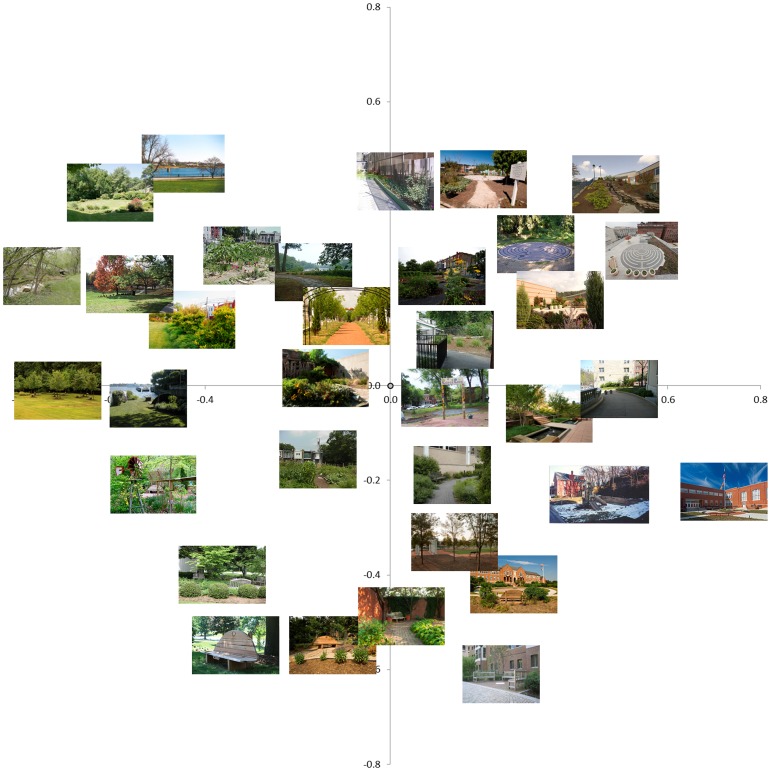
Plotted results of MDS dimensions 1 (X-axis) and 2 (Y-axis) for the *first* set, with pictures superimposed. The pictures are placed in the image based on their weights on dimension 1 and 2. A subset of the 70 images is plotted here because there are too many images to make this plot readable.

**Figure 3:**
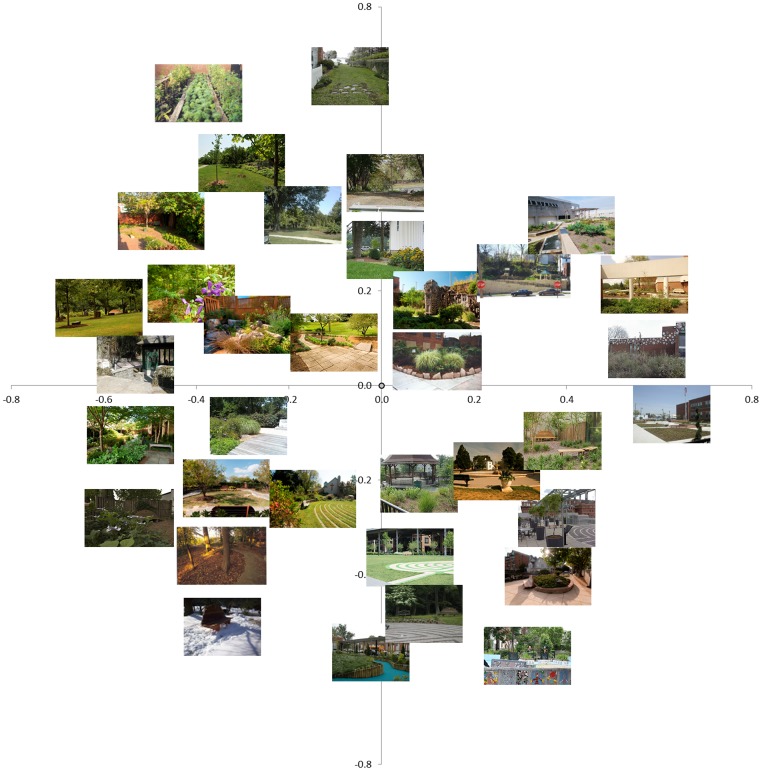
Plotted results of MDS dimensions 1 (X-axis) and 2 (Y-axis) for the *second* set, with pictures superimposed. The pictures are placed in the image based on their weights on dimension 1 and 2. A subset of the 70 images is plotted here because there are too many images to make this plot readable.

The results of the MDS analysis on the first set and the second set are displayed in Figs. 2 and 3. In those figures the scenes are superimposed on the resulting MDS plot so that the images are plotted based on their weights on dimension 1 and dimension 2. The data were scaled in 4 dimensions, as previously stated, in order to obtain the most appropriate organization of the space overall. However, we limited our forthcoming analyses on the weightings of dimensions 1 and 2, as those were the dimensions that explained the most variance in similarity.

To our eye, dimension 1 seemed to code for the naturalness of the images, with more ‘natural’ images having smaller/negative weights on dimension 1 and ‘built’ images having larger weights on dimension 1. It should be noted that the particular orientation of the dimensions is unimportant; what matters is the placement of items relative to other items. Thus, if the poles were reversed (i.e., ‘natural images had larger weights and ‘built’ images had smaller weights), the interpretation of a “naturalness” dimension would be unchanged.

Dimension 2 was a bit more difficult to characterize. Importantly, dimension 1 appeared to be similar for both the first set and second sets, suggesting that there was not something idiosyncratic about one set of images that produced these results. To validate that dimension 1 was coding for the naturalness of the scenes we conducted a second experiment.

## Experiment 2: Subjective Labeling of Dimensions

### Materials and Methods

To validate that dimension 1 (in both spaces) represented naturalness, we had naïve participants label dimensions 1 and 2 for the first set and the second set of images to identify what the most commonly composed labels were.

#### Participants

Fifty-seven participants from students from the University of Michigan and the University of South Carolina participated in our study (Michigan: 43 participants from two samples: Sample 1 Michigan 20 total, # female = 15; mean age = 19.95; Sample 2 Michigan 23 total, # female = 14, mean age = ∼24 (The ages were not recorded, but these were masters students so that mean age should be around 24); South Carolina: 14 participants, # female = 9, mean age = 20; note: the ages and gender of the participants were not recorded, but these were undergraduate students that matched the typical demographics from the experimental pool at the University of South Carolina). This research was approved by the Institutional Review Board of the University of Michigan (IRB #HUM00006681) and the Institutional Review Board of the University of South Carolina (IRB #Pro00028529). All participants provided written informed consent as administered by the institutional review board of the University of Michigan (IRB# HUM00006681) and the University of South Carolina (IRB# Pro00028529).

#### Procedure

We had participants provide subjective labels for dimensions 1 and 2 from the first set of images and from the second set images. Participants viewed posters of Figure 1 and 2 and were instructed to: “Come up with a single word or phrase that best describes to you what the difference is between the left and right of each poster, and what the difference is between the top and bottom of each poster. This means that you will be coming up with two labels for each poster, one for left/right, and one for top/bottom.” Participants then told an experimenter their labels for dimensions 1 and 2 for the first and second posters. These labels were then aggregated for analysis.

#### Analysis

From our reading of the generated labels, it appeared that the words nature or natural occurred frequently as descriptions of dimension 1. We performed a simple analysis where we counted the number of words that were repeated for the labels for the first dimension for the first and second sets of images. In addition, we removed preposition words from the labels, such as ‘to’ and ‘left’, to restrict the analysis to words with semantic content.

### Experiment 2 Results

Some of the most common themes that were uncovered from our analysis were: buildings (10 times listed), nature (9 times listed), space (8 times listed), paths (7 times listed), pathways (7 times listed), path (6 times listed), natural (5 times listed), gardens (5 times listed), manmade (4 times listed), organic (4 times listed), softscape hardscape (3 times listed) and difference (3 times listed). No other word was listed more than once. From this analysis it appeared that naïve participants were seeing what we were seeing, i.e., that dimension 1 seemed to be coding for something more vs. less natural or more built vs. more organic.

## Experiment 3: Rating the Perceived Naturalness of the Images

Based on the results of the poster labeling experiment, it appeared that MDS dimension 1 was coding for naturalness (low-scores) vs. manmadeness (high-scores). To test this directly, we had participants rate the perceived naturalness of each image.

### Materials and Methods

#### Participants

Fourteen participants from the University of Michigan participated in our study (mean age = 19.2; # female = 7). This research was approved by the Institutional Review Board of the University of Michigan (IRB #HUM00006681). All participants provided written informed consent as administered by the institutional review board of the University of Michigan (IRB# HUM00006681).

#### Materials

For this experiment, we added 50 high-natural and 50 low-natural images that were used in [1]. These include scenery of Nova Scotia and pictures of Ann Arbor, Detroit and Chicago. We added these images to 207 images that were from 87 TKF sites (images from 87 areas in urban parks from Annapolis, Baltimore and Washington), giving us a total of 307 images. These TKF images were all used in Experiment 1, but we included additional TKF images here that were not in Experiment 1 to have participants' rate naturalness on a larger set of data. In addition, in Experiment 1 the images were re-sized to be smaller, but the images in this experiment were shown in their native resolution and were in three different sizes: 512*384, 685*465, and 1024*680 pixels. Importantly, all image features were normalized to the size of the images.

#### Procedure

Participants provided their ratings of naturalness on all 307 images. Participants were shown a single image at a time and rated it on a scale of 1 to 7 for how natural they considered the image to be. A ‘1’ indicated that the participants considered the image to be very manmade and ‘7’ indicated that participants considered the image to be very natural. A ‘4’ indicated that the image was not judged to be very natural or manmade (therefore anything below 4 was judged to be more manmade and anything above 4 was judged as being more natural). We then correlated these values with the weights on dimension 1 to check for correspondence between the ratings of naturalness and the latent variable ‘naturalness’ as revealed in the MDS analysis.

### Results

**Figure 4:**
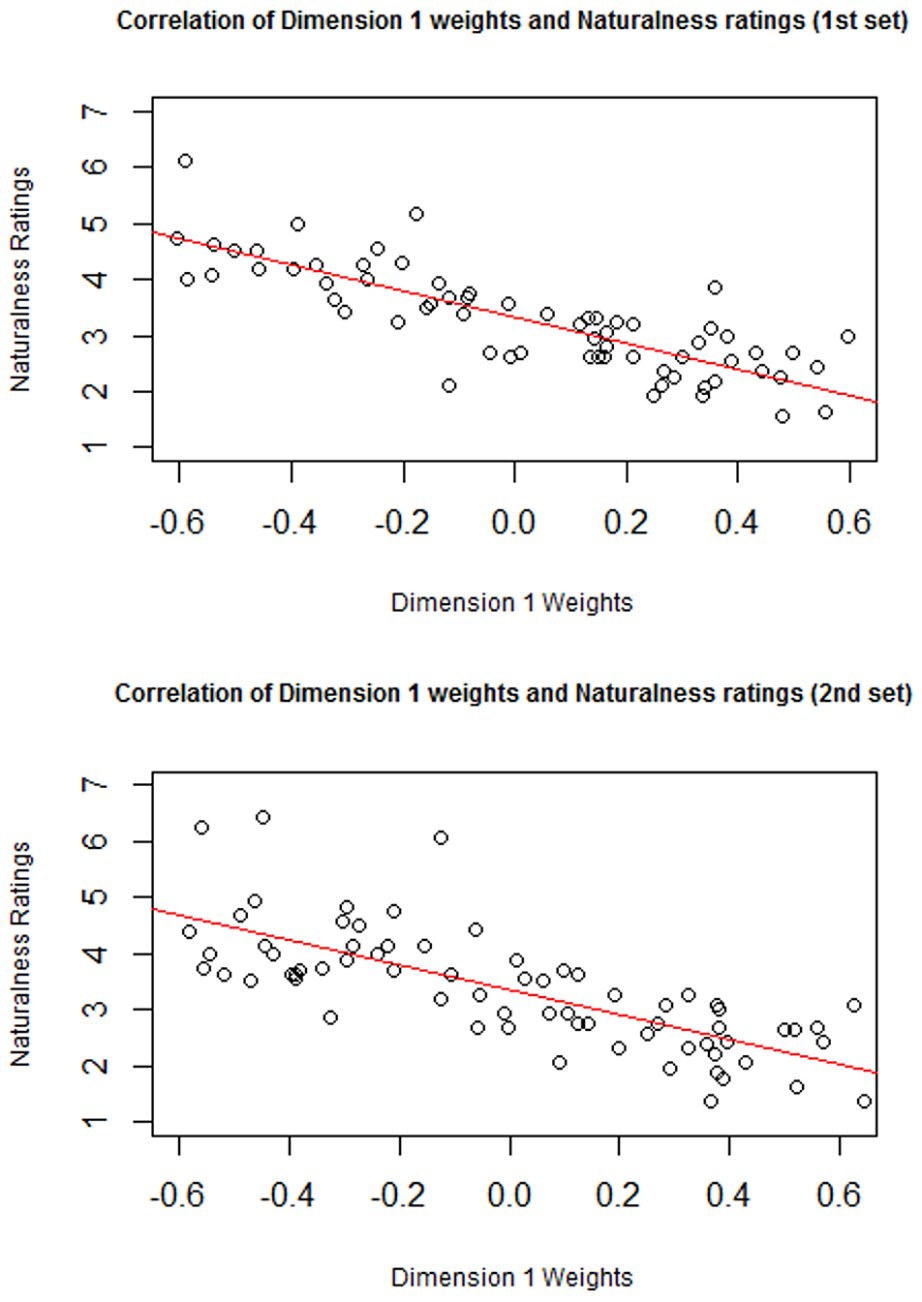
Correlation of Perceived Naturalness with weights on Dimension 1 for the first set and second sets of images.

Significant correlations were found between perceived naturalness ratings and weights on dimension 1 for both the first set, r(70) = −.84, p<.0001 and the second set, r(70) = −.75, p<.0001. The scatter plots can be seen in Fig. 4. The correlations are negative because negative weights on dimension 1 indicate more ‘naturalness.’

The significant correlation between weights on dimension 1 from MDS and the direct naturalness ratings suggests that MDS dimension 1 is coding for naturalness. In many respects, weights on dimension 1 can be interpreted as representing latent naturalness as participants were simply rating the similarity of the images, and naturalness could have been one of many factors that was used to rate similarity. Taken together, this suggests that the MDS analysis produced highly interpretable and reliable dimensions.

## Classifying Naturalness with Low-level Visual Features

The analyses thus far have established that individuals have consistent perceptions of what they consider to be natural images and that this dimension explains a good deal of variance in people's ratings of similarity of urban parks. Another question that we asked is whether low-level, objective visual features were related to subjective measures of naturalness. If we can find significant relationships between visual features and perceived naturalness, then it is possible that those features most related to naturalness may produce the positive psychological benefits that are attained from interactions with natural environments.

### Low-Level Visual Features

Ten low-level visual features were used in our analysis and were correlated with the perceived naturalness ratings to see if any low-level visual features were related to perceived naturalness. These features were divided into color properties and spatial properties.

#### Color properties

**Figure 5:**
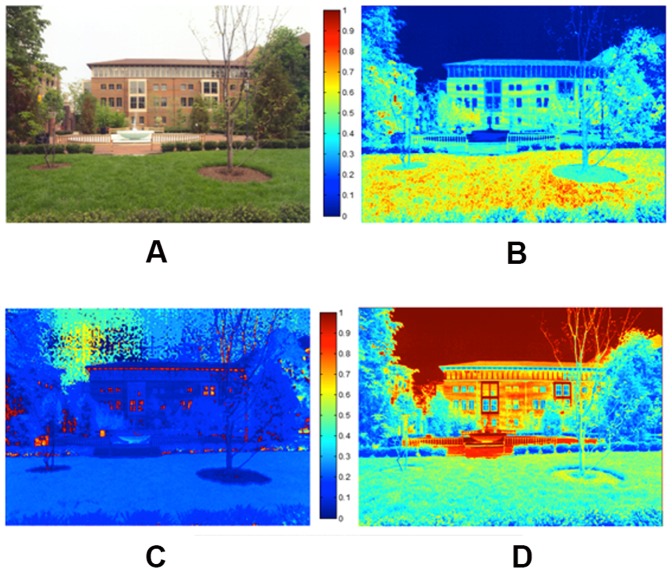
a) A sample image (b) Image's saturation map (c) Image's hue map (d) Image's brightness map.

**Figure 6:**
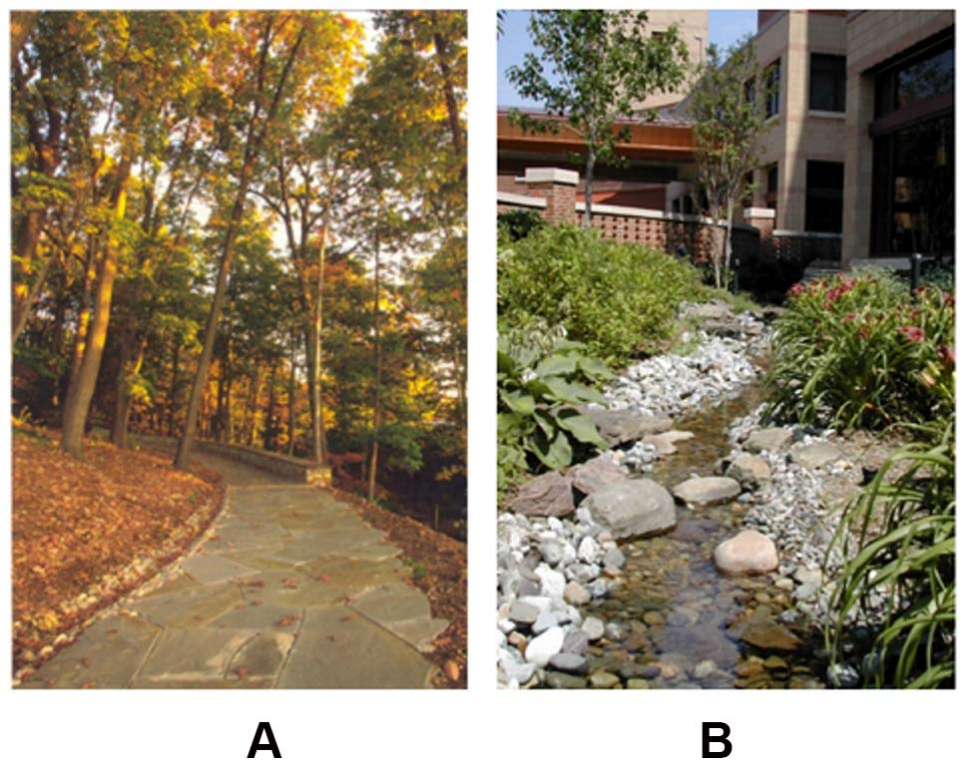
Comparison of two images in their color diversity properties. a) SDHue  = 0.11, SDSat = 0.22, SDbright = 0.21 b) SDHue = 0.19, SDSat = 0.26, SDbright = 0.26.

Color properties of the images were calculated based on the standard HSV model (Hue, Saturation, and Value) using the MATLAB image processing toolbox built-in functions (MATLAB and Image Processing Toolbox Release 2012b, The MathWorks, Inc., Natick, Massachusetts, United States). 1) **Hue** is the degree to which a stimulus can be described as similar to or different from stimuli that are described as red, green, or blue. Hue describes a dimension of color that is readily experienced (i.e., the dominant wavelength in the color). We calculated the average hue across all image pixels and the average standard deviation of hue across all of an image's pixels for each image. The average hue represents the hue level of the image and the 2) **standard deviation of hue** (SDhue) represents the degree of diversity in the image's hue. 3) **Saturation** (Sat) is the degree of dominance of hue mixed in the color, or the ratio of the dominant wavelength to other wavelengths in the color. We calculated the average saturation of each image across all image pixels, as well as the 4) **standard deviation of saturation** for each image (SDsat). We also measured the overall darkness-to-lightness of a pixel's color depending on the brightness of the pixel. This dimension of color is called 5) **Brightness** (Bright) or the value of the color. We computed the average brightness of all pixels for each image, as well as the 6) **standard deviation of brightness** in each image (SDbright). Fig. 5 shows hue, saturation, and brightness maps of a sample image in our experiment, and Fig. 6 compares two images in terms of their color diversity (SDHue, SDSat and SDbright).

#### Spatial Properties

**Figure 7:**
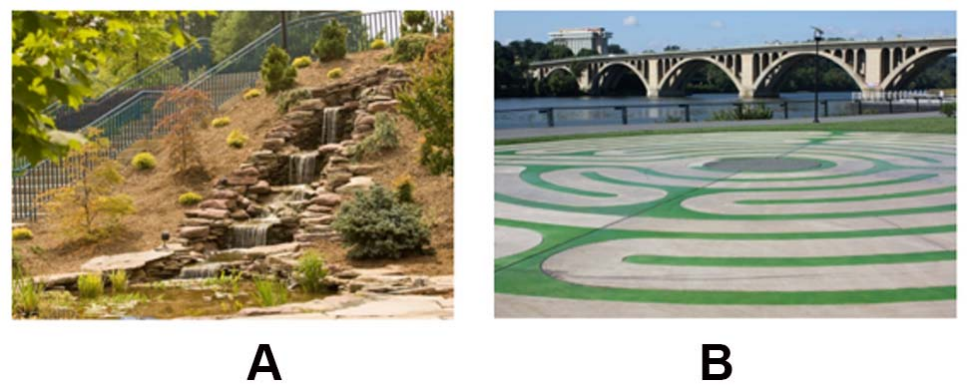
Comparison of two images in their Entropy a) Entropy = 7.63 b) Entropy = 7.10.

In this section we describe how we calculated the spatial features of our images. A greyscale histogram of an image shows the distribution of intensity values of pixels that construct an image. Each pixel could have an intensity value of 0 to 255 (8-bit grayscale) and for a histogram with 256 bins, the probability value of the *n*th bin of the histogram (□_□_) shows the number of pixels in the image that have an intensity value of n-1 over the total number of pixels in the image. 7) **Entropy** of a grey scale image is a statistical measure of randomness that can be used to characterize part of the texture of an image using the intensity histogram. We used a simple definition of Entropy: 
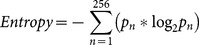
(Eq. 1)Where 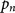 is the probability value of the *n*th bin of the histogram. Entropy shows the average “information” content of an image. The more the intensity histogram resembles a uniform distribution (all intensity values occur with the same probability in the image), the greater the entropy value becomes in the image. We calculated the entropy of the images as a measure of uncertainty or “information” content (versus redundancy) in the image's intensity values. More comprehensive and sophisticated definitions of image entropy have previously been applied for natural images, but those are out of the scope of this study. Here we aim to define simple features that are not computationally intensive to calculate and can be readily manipulated in visual stimuli ([35], [36]. Fig. 7 shows a comparison of high vs. low entropy in two images.

Another image feature that we calculated in this study concerned the spatial or structural properties of images provided by image gradients. An image gradient is a map of the image's brightness intensity or color changes in a given direction. The points of discontinuity in brightness (rapid brightness or color changes) mainly consisted of object, surface, or scene boundaries and fine details of texture in an image and are called edges. Images in this study (especially the more natural scenery) contain complex detailed texture and fragmentations, which could lead to some complexities in edge detection.

The most commonly used method for edge detection is the Canny edge detection algorithm [37]. This usually consists of five stages: first, blurring (or smoothing) an image with a Gaussian filter to reduce noise; second, finding the image gradients using derivatives of Gaussian operators; third, suppressing non-maximum gradient values; fourth, double thresholding weak and strong edges; and finally, edge tracking of weak or disconnected edges by hysteresis. This method is therefore less likely than the others to be influenced by noise, and more likely to detect true weak edges [38]. We used MATLAB's built in function ‘edge’ and set the method to ‘canny’ to calculate lower and upper thresholds to be used by the canny edge detection for each image. Then, the same function was used for each image with the determined sensitivity thresholds multiplied by either 0.8 (high sensitivity threshold) or 1.6 (low sensitivity threshold). We weighted faint and salient edges differently, so that each pixel could have a value of 0, 1, and 2 depending on how sharp of an edge it belonged to. Pixels assigned values of ‘0’ were not identified as edges by the canny edge detection algorithm at high sensitivity thresholds; pixels assigned values of ‘1’ were only detected as edges when using the high sensitivity threshold and not when using the less sensitive threshold (and therefore were less salient edges); finally, pixels assigned values of ‘2’ were detected as edges with the lower sensitivity threshold and therefore were the most salient.

Next, we quantified the pixels belonging to straight lines (horizontal, vertical and oblique lines) so that straight edge density and non-straight edge (curved or fragmented edges) density of images could be quantified and separated. Because of the complexity of the images, a typical Hough transform-based method could not detect straight lines accurately. Instead, we used a simple gradient-based connected component algorithm to detect straight lines in the images.

First, the images were convolved with the derivative of a Gaussian filter in the X and the Y directions to compute the gradient directions for Canny edges. Then each edge was assigned to one of 8 directions based on its value of 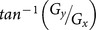 where G_y_ and G_x_ are the y and x gradients. Then the connected components for the edge pixels in each direction were determined and labeled using MATLAB's ‘bwconncomp’ function. Finally, the Eigenvalues of the covariance matrix of the X and the Y coordinates of points for each connected component (i.e., edge) were used to compute the direction (i.e., the direction of the first principal component vector) and the straightness of the components. The first PC of the edges' coordinates should be parallel to the edge's direction and the second PC captures the variability of the edge's coordinates perpendicular to its direction. Pixels of a connected component above a threshold of straightness (i.e., the singular value for the first principle component needed to be greater than 10^4^ times larger than the singular value for second component) met the criterion of a “straight edge.”

**Figure 8:**
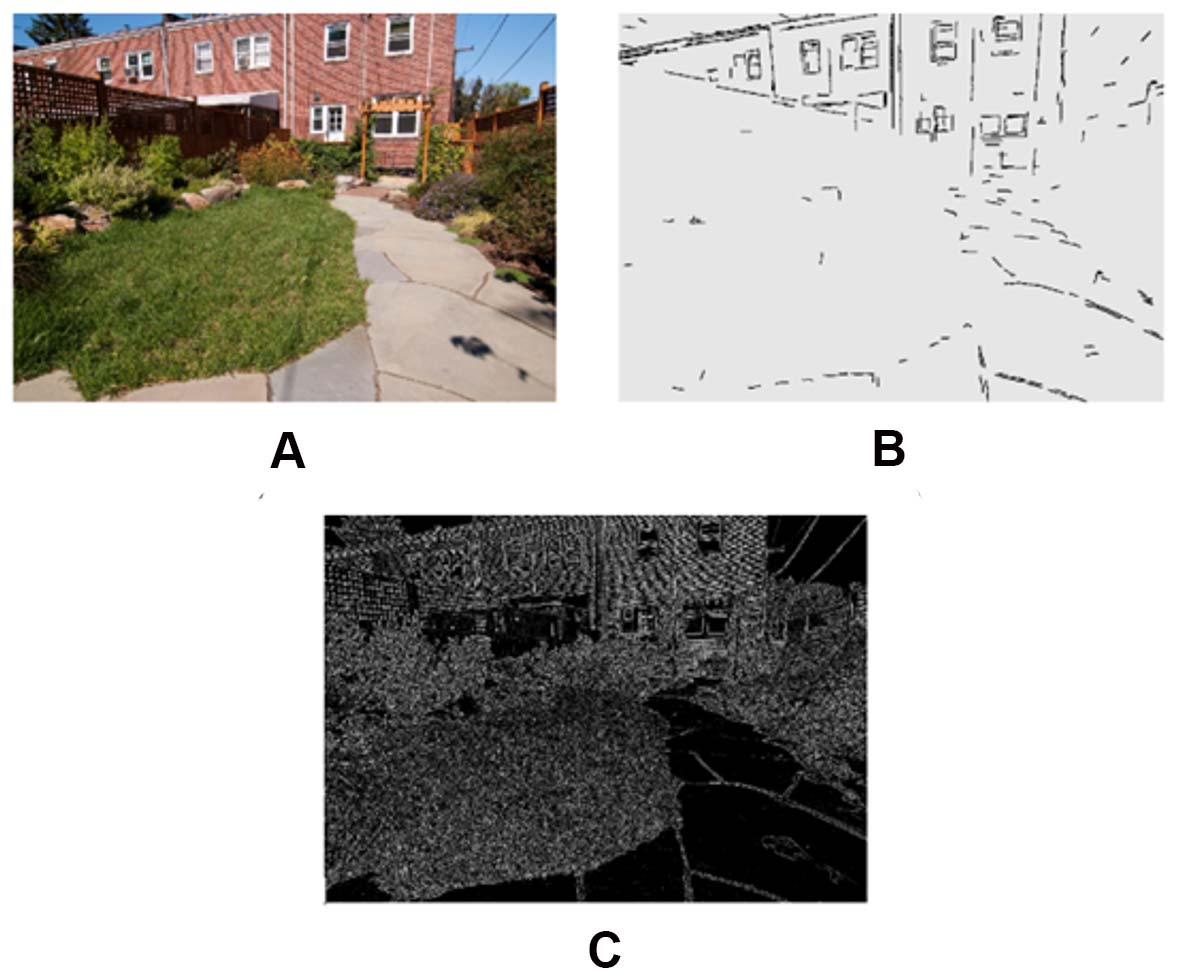
(a) Sample image, (b) the detected straight edges of the sample image(c) the edge density map of the sample image.

The number of pixels on straight edges and those on non-straight edges were divided by total number of pixels in the image to create 8) **Straight Edge Density** (SED), and 9) **None-straight edge density** (NSED). Fig. 8 shows maps of detected edges and straight edges in a sample image. Importantly, all of these features were normalized to the number of pixels in the image.

### Results

**Figure 9:**
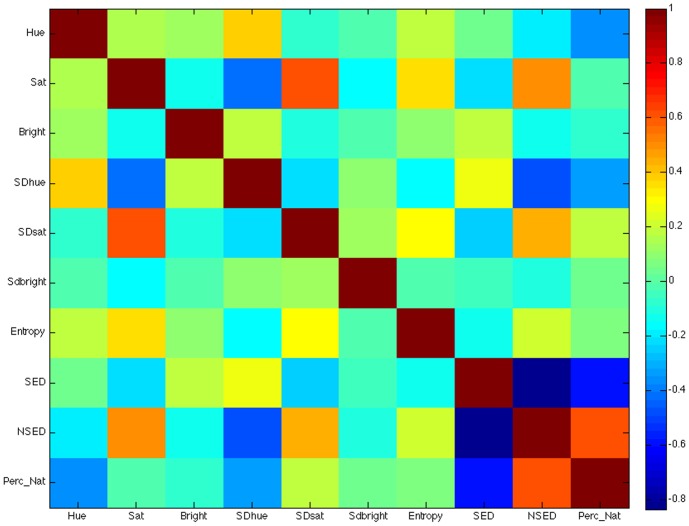
Correlation Matrix of low-level visual features with perceived naturalness. The color bar indicates the strength of the correlation from -.8 to 1.

Fig. 9 displays the correlation of the low-level visual features with perceived naturalness ratings. Hue, NSED, SED, SDhue, and SDsat all significantly correlated with naturalness. These data suggest that low-level visual features (objective measures) can be used to predict individuals' perceptions of naturalness. To test this more directly, we trained a linear discriminant classification algorithm to predict whether an image was rated as natural or not based on these low-level visual features.

### Linear and Quadratic Discriminant Classification of Perceived Naturalness

To examine how reliably these low-level visual image features predicted the perceived naturalness of the images, we trained two multivariate machine-learning algorithms, the linear discriminant classifier and the quadratic discriminant classifier, utilizing the low-level visual features to predict the perceived naturalness of the images. Utilizing a leave-one-out framework we could test how well each classifier could accurately predict the perceived naturalness of the image.

#### Methods

We implemented two multivariate machine-learning algorithms to classify individual's perceptions of naturalness of images based on low-level visual features of the images. The first classifier that we used was a Linear Discriminant (LD) classification algorithm that attempts to define a plane that separates two classes. Implementation of LD classification was performed using the ‘classify’ function in the Statistics toolbox in Matlab (the classifier type was set to ‘linear’). LD classifiers use a multivariate Gaussian distribution to model the classes and classify a vector by assigning it to the most probable class. The linear discriminant classification model contains an assumption of homoscedasticity, i.e., that all classes are sampled from populations with the same covariance matrix. For our purposes, this assumption means that (a) the variance of each low-level visual feature does not change for high vs. low natural images and (b) the correlation between each pair of features is the same for high and low natural images. Importantly, the assumption of homoscedasticity is equivalent to separating the two classes, (high and low natural images), with a linear plane in feature space. The plane is defined as a linear combination of features; the weight of each feature reflects the contribution of this feature to classification (that is, most relevant features have the highest absolute value of weight).

The second classification algorithm that we implemented was a Quadratic Discriminant (QD). Implementation of QD classification was performed using the ‘classify’ function in the Statistics toolbox in Matlab (the classifier type was set to ‘quadratic’). Like the LD classifier the QD classifier uses a multivariate Gaussian distribution to model the classes and classify a vector by assigning it to the most probable class. However, the QD model contains no assumption of homoscedasticity, and instead estimates the covariance matrices separately for each class (that is, the variances of and the correlations between features are allowed to differ across high vs. low-natural images). This indicates that when implementing QD the two classes are separated by a non-linear curved surface. Both LD and QD algorithms have been implemented with great success to classify brain states and participants brain activity patterns [39]–[41].

We evaluated the success of each classifier using a cross-validation approach. A subset of images was used to train the classifier, and the image type (high natural vs. low natural) was predicted for the images that were not included in the training set. At each iteration, two images (1 high-natural and 1 low-natural) were held out for testing, and the remaining 305 were used to train the classifier; this process was repeated so that all combinations of high and low natural images were determined by classification.

For each combination of left-out high- and low-natural image we computed whether the image type was predicted accurately. The proportion of images that were accurately predicted was our metric of prediction accuracy, our main measure of the efficacy of the classifier.

#### Results

**Figure 10:**
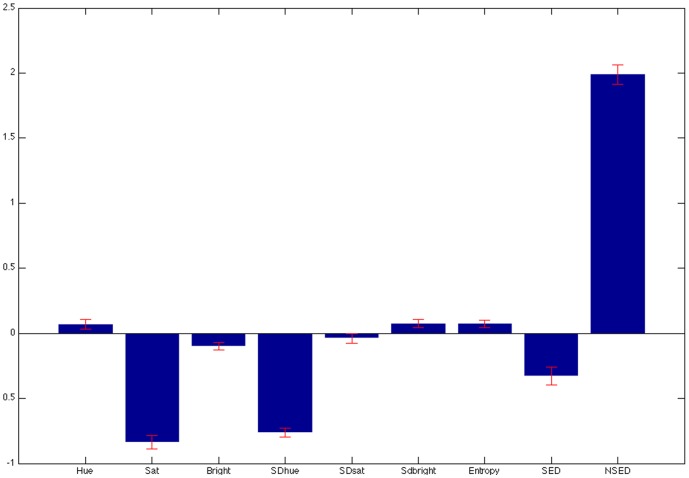
Feature weights for the LD classification algorithm in predicting high- vs. low- perceived naturalness of the images. A high absolute value of the weight indicates that that feature is important for classification. A positive weight indicates that that increasing this feature would lead to increased perceived naturalness; a negative weight indicates that increasing this feature would lead to a decrease in perceived naturalness. Error bars reflect 2 standard deviations from the mean.

The LD classifier was able to successfully predict whether an image was perceived as high- vs. low-natural with 79% accuracy. This prediction accuracy is well above chance performance (50%) and suggests that these low-level visual features reliably predict individuals' perceptions of naturalness. When we examined the features that appear most critical to classification, we found that edge density, the number of straight edges, and the standard deviation of hue were the most critical features. These feature weights are displayed in Fig. 10. More edge density, fewer straight edges and lower standard deviations in hue (less hue diversity) were all related to greater perceived naturalness.

In addition, we noticed that some features, such as the number of curved edges, had a non-linear relationship with ratings of naturalness. Therefore, we ran the classification analysis a second time, but using a non-linear classifier, i.e. the quadratic discriminant, which would capitalize on these non-linear relationships, as well as the interactions of them. When doing so classification accuracy increased to 81%.

Researchers have shown that the principle components of natural images from their frequency spectral maps could be sensitive in capturing some of the systematic properties of natural versus man-made scenery [16], [42], [43]. In order to find out how the visual features used in this study correlate with the spectral principal components of the images, we ran a principal component analysis on the images similar to that of [16]. We resized each image to 256*256 pixels and did a discrete Fourier transform on each image, and then reshaped it to a single column vector (65536*1). All 307 spectral images were then aggregated into a 65536*307 matrix and a principal component analysis was performed on the concatenated image matrix.

**Figure 11:**
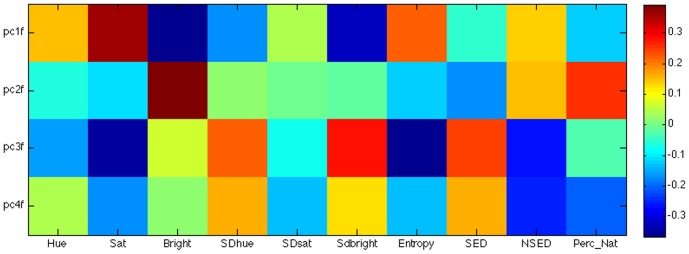
Correlation Matrix of low-level visual features with the first 4 Principal Components across the 307 images. The color bar indicates the strength of the correlation from -.4 to +.4

The first 4 principal components explained 95% of the variability in the magnitude of Fourier coefficients between images and were correlated with the low-level visual features. Fig. 11 shows the correlation matrix of these PC's with the simple image features. The PCs did correlate significantly with some of our features such as many of the color features, entropy, SED and NSED. As such, we performed another classification analysis utilizing these 4 principal components to predict perceived naturalness. When doing so we obtained above chance accuracy for both LD (prediction accuracy = 60.4%) and QD (prediction accuracy = 64.0%). However, classification with these PCs was not as strong as the classification accuracy calculated with the derived low-level features. In summary, these features significantly predicted ratings of perceived naturalness, linking low-level objective measures to subjective measures of naturalness.

To further inspect the relation of these visual features with the naturalness dimension we previously obtained from the MDS analysis, we also regressed the weights on dimension 1 on the image features for both the first set and second set of stimuli. The results show that these features explain a significant amount of variance in dimension 1 weights (40% for first set and 35% for odd set; p<10^−6^ and p<10^−5^, respectively). The results for the regressions are shown in Tables 1 and 2. These results complement the results from the classification analysis and show that low-level features do reliably predict subjective perceptions of naturalness.

**Table 1:** Regression of Dimension 1 on features from the First set experiment.

Measure	β	SE	CI	t-value
SDhue	0.16*	0.04	[0.08, 0.24]	3.21
Entropy	0.14*	0.04	[0.06, 0.22]	3.30
NSED	−0.19*	0.04	[−0.27, −0.11]	−4.64
SED	0.15*	0.04	[0.07, 0.24]	3.06
Saturation	0.01	0.04	[−0.07, 0.09]	0.28
Brightness	−0.05	0.04	[−0.13, 0.03]	−1.22
Hue	−0.08	0.05	[−0.18, 0.02]	−1.54
SDbright	0.05	0.04	[−0.03, 0.13]	1.47

Note. Adjusted R^2^ = 0.40, F (8, 61) = 6.66.

*P<0.05, CI  =  Confidence interval for α = 0.05, SE = Standard error.

**Table 2:** Regression of dimension 1 on features from the Second set experiment.

Measure	β	SE	CI	t-value
SDhue	0.11*	0.04	[0.04, 0.18]	2.53
Entropy	0.06	0.04	[−0.02, 0.14]	1.28
NSED	−0.13*	0.04	[−0.27, −0.11]	−2.88
SED	0.08*	0.04	[0.01, 0.15]	2.00
Saturation	0.03	0.05	[−0.07, 0.13]	0.70
Brightness	−0.01	0.04	[−0.09, 0.07]	−0.26
Hue	−0.05	0.04	[−0.13, 0.03]	−1.38
SDbright	0.06	0.04	[−0.02, 0.14]	1.62

Adjusted R^2^ = 0.34, F (8, 61) = 5.86.

Note. *P<0.05, CI  =  Confidence interval for α = 0.05, SE = Standard error.

## Discussion

Previous research has shown that interacting with natural environments can have a salubrious effect on cognitive and affective processing compared to interacting with more urban/manmade environments [1], [3], [5]. This suggests that there is something about natural environments that differs from urban environments that could improve psychological functioning.

The problem is that finding such features is difficult given how many features differ between these two environments. In this work, we found that individuals' perceptions of naturalness are quite consistent. This is corroborated by the fact that in the MDS analysis, the dimension that explained the most variance in individuals' perceptions of similarity in scenes was strongly related to direct ratings of naturalness. To take this one step further, we were able to link perceptions of naturalness with objective low-level visual features such as the density of edges, straight lines and hue diversity. This means that we have objective measures that significantly predict perceived naturalness and therefore may also be features that could be manipulated to improve psychological functioning.

Notably, our work replicates important previous research by Ward and colleagues. In that work, a sample of 20 photographs was used with more extreme levels of natural content (e.g., inside a rainforest, the Grand Canyon) and urban content (e.g., an aerial view of San Francisco, an smoggy freeway). With these images, Ward and colleagues had participants make pairwise similarity judgments between the 20 images (i.e., 190 pairs) and then ran an MDS analysis. Importantly, the first dimension that was uncovered from those experiments coded for the naturalness vs. constructed character of the images[26], [28]. In another study naturalness was also correlated with dimension 1 weights, but was confounded with the openness vs. enclosedness of the images [27]; it is also worth noting that the number of significant dimensions in those studies was also around 4–5, which is similar to our study). The results presented here replicate this earlier work on a much larger set of images and with less variability in image content (i.e., our images were not just at the extremes of naturalness vs. manmadeness, but had a large distribution with many intermediately rated images). Therefore, our more restricted range of environments may better represent the types of environments encountered in daily life. In addition, the results also demonstrate that the phenomena are measurable even across a more subtle range of naturalness.

Importantly, this work extends upon the previous findings by identifying low-level visuals features that could be manipulated to uncover if any of these features may improve psychological outcomes (i.e., attention and mood). Without identifying the features that are related to naturalness, it would be difficult to construct an experiment aimed at uncovering physical features of the environment that may lead to improvements in psychological functioning, and even more difficult to design a future built environment to improve psychological functioning. Our work helps to provide a foundation and methods for classifying and quantifying our physical environment in psychologically and behaviorally meaningful ways [26], [44].

Additionally, many researchers have achieved great success in training machine-learning algorithms on low-level features to classify natural vs. man-made environments using sophisticated analyses [16], [17]. Here we utilized simpler metrics to link with subjective measures of perceived naturalness, because our goal was to define simple, objective measures that could be easily manipulated (e.g., the number of curved and fragmented edges, average color diversity or the number of straight lines) to study if those features may improve psychological functioning.

One limitation of this study is the fact that we used images of natural and urban environments, which are by definition abstractions of the true environment. More specifically, JPEG and other lossy image compression schemes can alter spatial and color image statistics [45]. One could draw conclusions about human perceptions of naturalness for compressed scenes in terms of their statistics, but this may not capture human perception of natural scenes and urban scenes in the “wild.” However, the fact that [1]used some of the images that were also used here and found restorative effects (i.e., improvements in memory and attention) after exposure to those natural images suggests that compressed images do preserve at least some of the visual properties of scenes that lead to psychological benefits. Additionally, there are practical considerations when performing such studies and it would be difficult to quantify many of these low-level features in the wild.

Another potential limitation is that one may be able to create abstract images that contain many of the features of nature, but are not perceived as being natural (e.g., a Jackson Pollock painting). This would make classifying those images difficult, but it is possible that exposure to abstract art that contains many of the low-level features of nature could be restorative and is a topic that we plan to pursue in the future, i.e., whether the low-level features on there own are restorative or if the semantic meaning of nature is necessary for restoration.

Along these same lines, it is not clear if the low-level visual features used in our study objectively measure naturalness. For example, our data suggest that environments perceived as more natural contain more non-straight edges, fewer straight edges, and less color saturation, but it could be that other non-natural environments could also be found to share some of these distributional properties that we are finding for our natural environments (e.g., some of Antoni Gaudi's architecture in Barcelona). As such it is not necessarily the case that these low-level visual features define naturalness per se. However, based on the success of our algorithms in predicting the perceptions of naturalness, we are confident that these low-level features are related to naturalness, but may not be exclusive to natural environments. More images and scene-types would be needed to draw more definitive conclusions.

In his seminal 1995 paper, Kaplan lists some criteria that would appear to be important for a natural environment to be restorative: The environment must have sufficient extent; the environment must be compatible with one's goals; the environment must give people the sense of being away; and the environment must be fascinating [10]. We have attempted to take the work of Kaplan one step farther by defining the low-level visual features that may drive perceptions of naturalness with the hope that these features may be causal to the beneficial effects of interacting with natural environments. It is our hope that in future work we can more fully identify the features of nature that may lead to psychological benefits so that those features can be utilized in future designs of the built environment.

## Supporting Information

S1 Data **Feature values for the images used in our study.**
(XLS)

S2 Data **Images used in our study.**
(ZIP)

## References

[1] Berman MG, Jonides J, Kaplan S (2008) The Cognitive Benefits of Interacting With Nature. Psychological Science 19:1207. 19121124 10.1111/j.1467-9280.2008.02225.x

[2] Kaplan S, Berman MG (2010) Directed Attention as a Common Resource for Executive Functioning and Self-Regulation. Perspectives on Psychological Science 5:43. 26162062 10.1177/1745691609356784

[3] Berto R (2005) Exposure to restorative environments helps restore attentional capacity. Journal of Environmental Psychology 25:249.

[4] Taylor AF, Kuo FE (2009) Children With Attention Deficits Concentrate Better After Walk in the Park. Journal of Attention Disorders 12:402. 18725656 10.1177/1087054708323000

[5] Berman MG, Kross E, Krpan KM, Askren MK, Burson A, et al (2012) Interacting with nature improves cognition and affect for individuals with depression. Journal of Affective Disorders 140:300–305. 22464936 10.1016/j.jad.2012.03.012 PMC3393816

[6] Cimprich B, Ronis DL (2003) An environmental intervention to restore attention in women with newly diagnosed breast cancer. Cancer nursing 26:284. 12886119 10.1097/00002820-200308000-00005

[7] Kuo FE, Sullivan WC (2001) Environment and crime in the inner city - Does vegetation reduce crime? Environment and Behavior 33:343.

[8] Kuo FE, Sullivan WC (2001) Aggression and violence in the inner city - Effects of environment via mental fatigue. Environment and Behavior 33:543.

[9] Ulrich RS (1984) VIEW THROUGH A WINDOW MAY INFLUENCE RECOVERY FROM SURGERY. Science 224:420–421. 6143402 10.1126/science.6143402

[10] Kaplan S (1995) The Restorative Benefits of Nature - Toward an Integrative Framework. Journal of Environmental Psychology 15:169.

[11] Ulrich RS, Simons RF, Losito BD, Fiorito E, Miles MA, et al (1991) Stress Recovery during Exposure to Natural and Urban Environments. Journal of Environmental Psychology 11:201.

[12] Mayer FS, Frantz CM, Bruehlman-Senecal E, Dolliver K (2009) Why Is Nature Beneficial? The Role of Connectedness to Nature. Environment and Behavior 41:607.

[13] Wilson EO (1984) Biophilia. Cambridge, MA: Harvard University Press.

[14] Kellert SR, Wilson EO (1993) The Biophilia Hypothesis. Washington, D.C.: Island Press.

[15] Oliva A, Torralba A (2001) Modeling the shape of the scene: A holistic representation of the spatial envelope. International journal of computer vision 42:145–175.

[16] Torralba A, Oliva A (2003) Statistics of natural image categories. Network: computation in neural systems 14:391–412. 12938764

[17] Fei-Fei L, Perona P. A bayesian hierarchical model for learning natural scene categories; 2005. IEEE. pp. 524–531.

[18] Huang J, Mumford D. Statistics of natural images and models; 1999. IEEE.

[19] Ruderman DL (1994) The statistics of natural images. Network: computation in neural systems 5:517–548.

[20] Field DJ (1987) Relations between the statistics of natural images and the response properties of cortical cells. JOSA A 4:2379–2394. 10.1364/josaa.4.002379 3430225

[21] van Hateren JH, van der Schaaf A (1998) Independent component filters of natural images compared with simple cells in primary visual cortex. Proceedings of the Royal Society of London Series B: Biological Sciences 265:359–366. 9523437 10.1098/rspb.1998.0303 PMC1688904

[22] Olshausen BA (1996) Emergence of simple-cell receptive field properties by learning a sparse code for natural images. Nature 381:607–609. 8637596 10.1038/381607a0

[23] Baddeley RJ, Hancock PJ (1991) A statistical analysis of natural images matches psychophysically derived orientation tuning curves. Proceedings of the Royal Society of London Series B: Biological Sciences 246:219–223. 1686086 10.1098/rspb.1991.0147

[24] Shepard RN (1980) Multidimensional scaling, tree-fitting, and clustering. Science 210:390–398. 17837406 10.1126/science.210.4468.390

[25] Hout MC, Papesh MH, Goldinger SD (2013) Multidimensional scaling. Wiley Interdisciplinary Reviews: Cognitive Science 4:93–103. 23359318 10.1002/wcs.1203 PMC3555222

[26] Ward LM (1977) Multidimensional scaling of the molar physical environment. Multivariate Behavioral Research 12:23–42. 26804142 10.1207/s15327906mbr1201_2

[27] Ward LM, Porter CA (1980) Age-group differences in cognition of the molar physical environment: A multidimensional scaling approach. Canadian Journal of Behavioural Science/Revue canadienne des sciences du comportement 12:329.

[28] Ward LM, Russell JA (1981) Cognitive set and the perception of place. Environment and Behavior 13:610–632.

[29] Goldstone R (1994) AN EFFICIENT METHOD FOR OBTAINING SIMILARITY DATA. Behavior Research Methods Instruments & Computers 26:381–386.

[30] Hout MC, Goldinger SD, Ferguson RW (2013) The versatility of SpAM: A fast, efficient, spatial method of data collection for multidimensional scaling. Journal of Experimental Psychology: General 142:256. 22746700 10.1037/a0028860 PMC3465534

[31] Kriegeskorte N, Mur M (2012) Inverse MDS: inferring dissimilarity structure from multiple item arrangements. Frontiers in psychology 3. 10.3389/fpsyg.2012.00245 PMC3404552 22848204

[32] Doyen J, Hubaut X, Vandensavel M (1978) Ranks of incidence matrices of Steiner triple systems. Mathematische Zeitschrift 163:251–259.

[33] Busing F, Commandeur JJ, Heiser WJ, Bandilla W, Faulbaum F (1997) PROXSCAL: A multidimensional scaling program for individual differences scaling with constraints. Softstat 97:67–74.

[34] Jaworska N, Chupetlovska-Anastasova A (2009) A review of multidimensional scaling (MDS) and its utility in various psychological domains. Tutorials in Quantitative Methods for Psychology 5:1–10.

[35] Kersten D (1987) Predictability and redundancy of natural images. JOSA A 4:2395–2400. 10.1364/josaa.4.002395 3430226

[36] Chandler DM, Field DJ (2007) Estimates of the information content and dimensionality of natural scenes from proximity distributions. JOSA A 24:922–941. 17361279 10.1364/josaa.24.000922

[37] Klette R, Zamperoni P (1996) Handbook of image processing operators. Handbook of image processing operators, by Klette, Reinhard; Zamperoni, Piero Chichester; New York: Wiley, 1996 1.

[38] Canny J (1986) A COMPUTATIONAL APPROACH TO EDGE-DETECTION. Ieee Transactions on Pattern Analysis and Machine Intelligence 8:679–698. 21869365

[39] Berman MG, Yourganov G, Askren MK, Ayduk O, Casey BJ, et al. (2013) Dimensionality of brain networks linked to life-long individual differences in self-control. Nature Communications 4. 10.1038/ncomms2374 PMC3555568 23340413

[40] Yourganov G, Schmah T, Churchill NW, Berman MG, Grady CL, et al (2014) Pattern classification of fMRI data: Applications for analysis of spatially distributed cortical networks. NeuroImage 96:117–132. 24705202 10.1016/j.neuroimage.2014.03.074

[41] Yourganov G, Schmah T, Small SL, Rasmussen PM, Strother SC (2010) Functional connectivity metrics during stroke recovery. Archives italiennes de biologie 148:259–270. 21175012

[42] Field D (1999) Wavelets, vision and the statistics of natural scenes. Philosophical Transactions of the Royal Society of London Series A: Mathematical, Physical and Engineering Sciences 357:2527–2542.

[43] Hancock PJ, Baddeley RJ, Smith LS (1992) The principal components of natural images. Network: computation in neural systems 3:61–70.

[44] Craik KH (1973) Environmental psychology. Annual review of psychology 24:403–422.

[45] Leykin A, Cutzu F. Differences of edge properties in photographs and paintings; 2003. IEEE. pp. III-541-544 vol. 542.

