# Supplementary figures and images for: The Perception of Naturalness Correlates with Low-Level Visual Features of Environmental Scenes (part 2 of 2)
Source: PLoS One. 2014 Dec 22;9(12):e114572. doi: 10.1371/journal.pone.0114572 (PMC4273965; doi:10.1371/journal.pone.0114572)

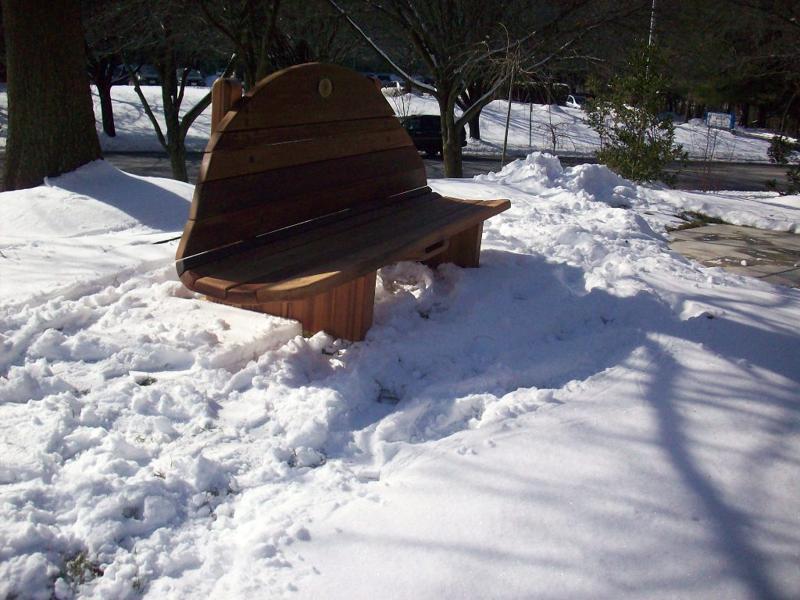

Supplement: S2 Data — Images used in our study. (ZIP) [file pone.0114572.s002.zip › Stimuli/MDS600X800/MDS19.jpg]

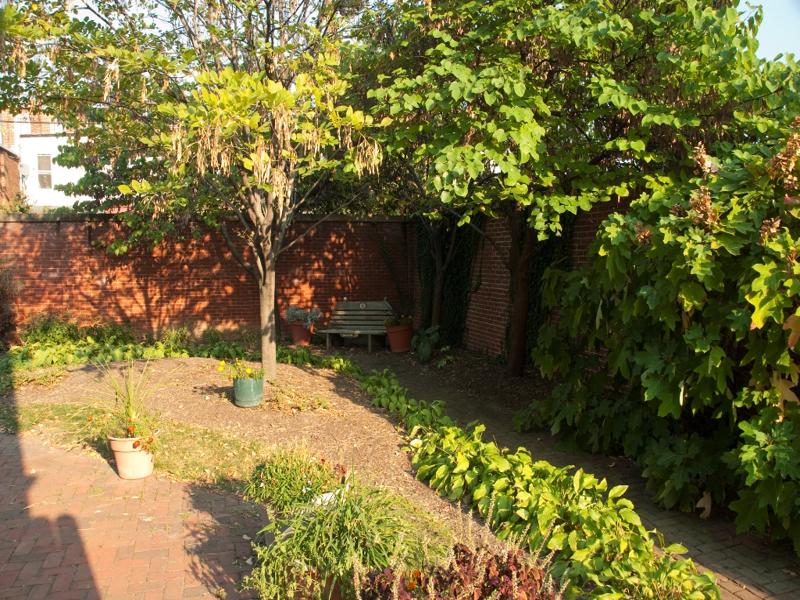

Supplement: S2 Data — Images used in our study. (ZIP) [file pone.0114572.s002.zip › Stimuli/MDS600X800/MDS190.jpg]

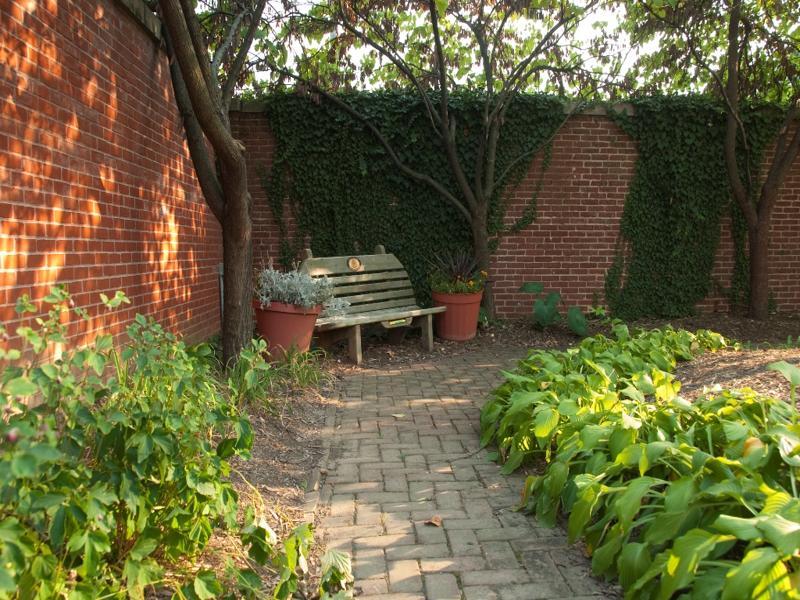

Supplement: S2 Data — Images used in our study. (ZIP) [file pone.0114572.s002.zip › Stimuli/MDS600X800/MDS191.jpg]

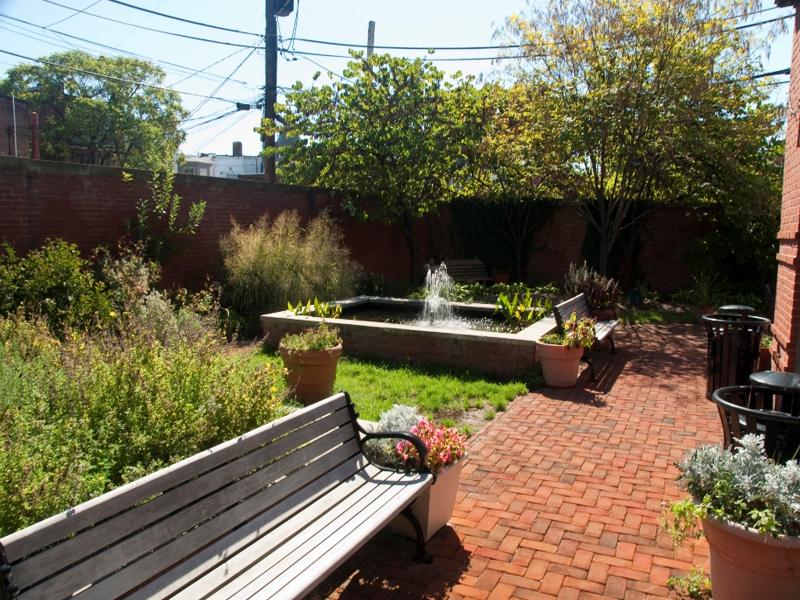

Supplement: S2 Data — Images used in our study. (ZIP) [file pone.0114572.s002.zip › Stimuli/MDS600X800/MDS192.jpg]

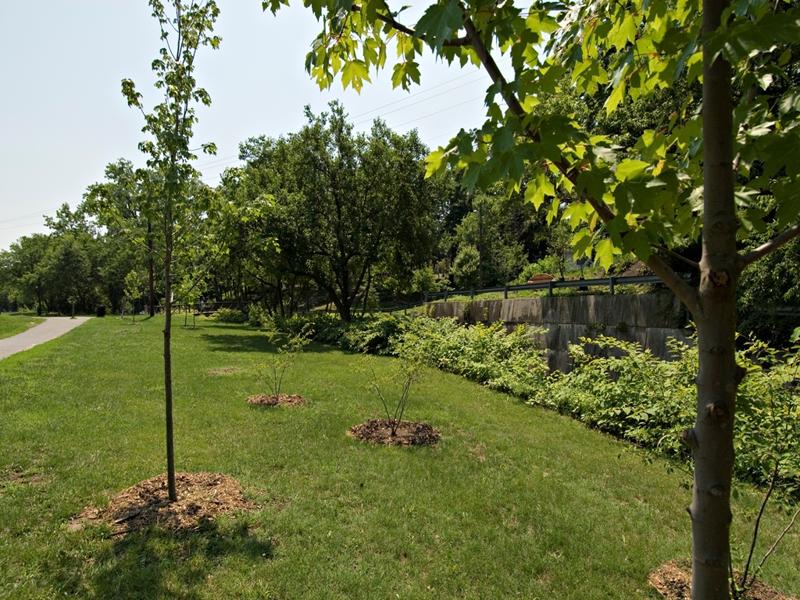

Supplement: S2 Data — Images used in our study. (ZIP) [file pone.0114572.s002.zip › Stimuli/MDS600X800/MDS193.jpg]

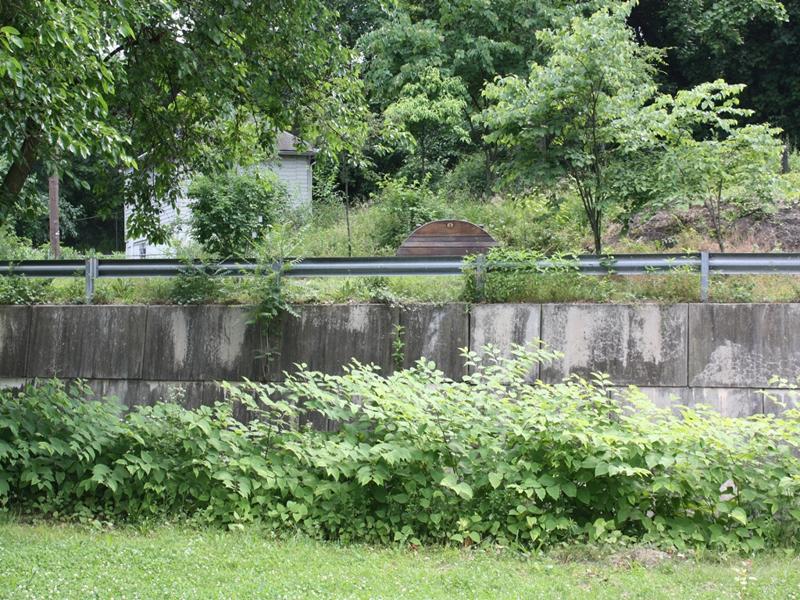

Supplement: S2 Data — Images used in our study. (ZIP) [file pone.0114572.s002.zip › Stimuli/MDS600X800/MDS194.jpg]

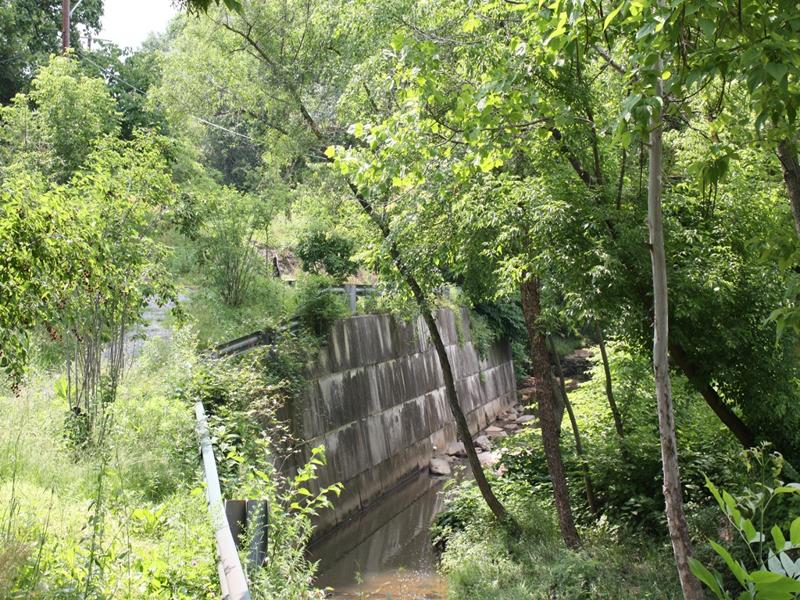

Supplement: S2 Data — Images used in our study. (ZIP) [file pone.0114572.s002.zip › Stimuli/MDS600X800/MDS195.jpg]

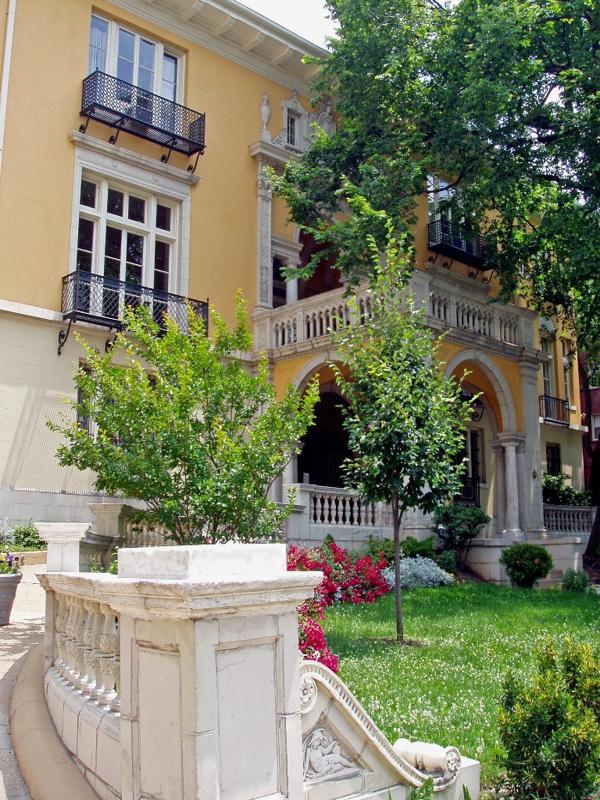

Supplement: S2 Data — Images used in our study. (ZIP) [file pone.0114572.s002.zip › Stimuli/MDS600X800/MDS196.jpg]

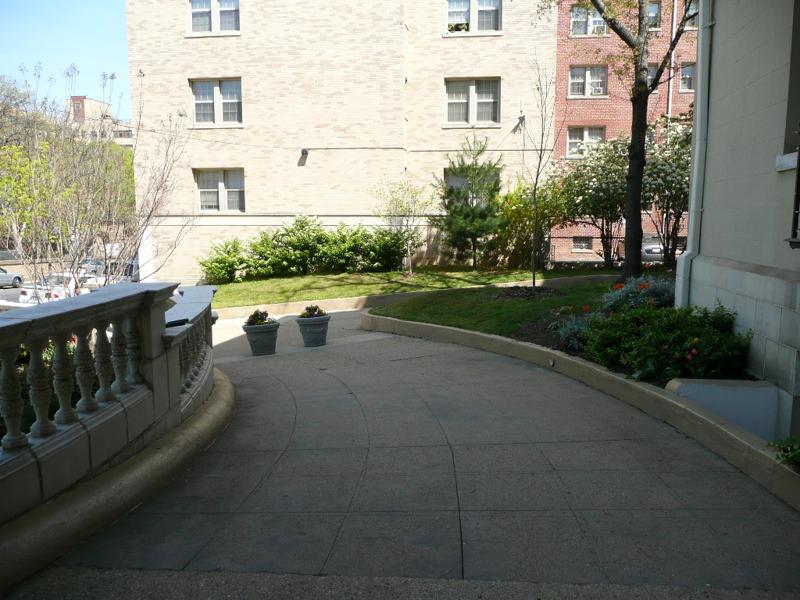

Supplement: S2 Data — Images used in our study. (ZIP) [file pone.0114572.s002.zip › Stimuli/MDS600X800/MDS197.jpg]

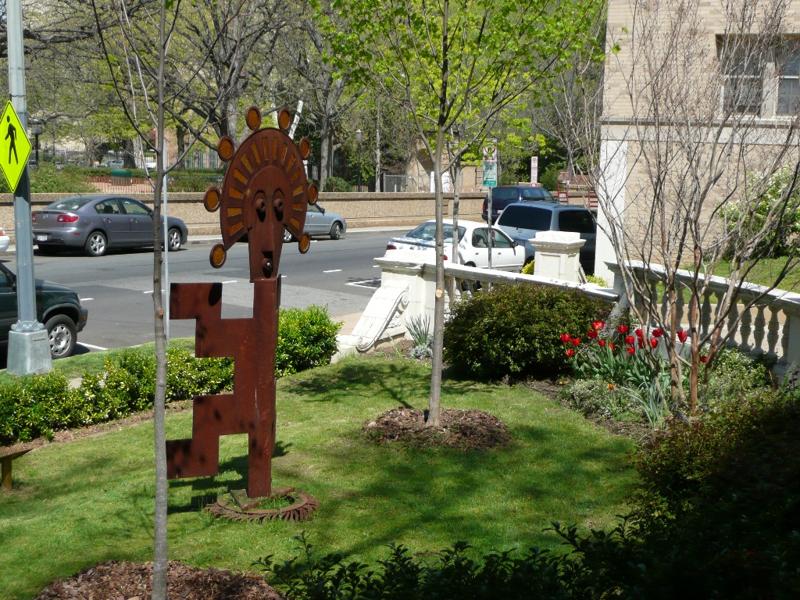

Supplement: S2 Data — Images used in our study. (ZIP) [file pone.0114572.s002.zip › Stimuli/MDS600X800/MDS198.jpg]

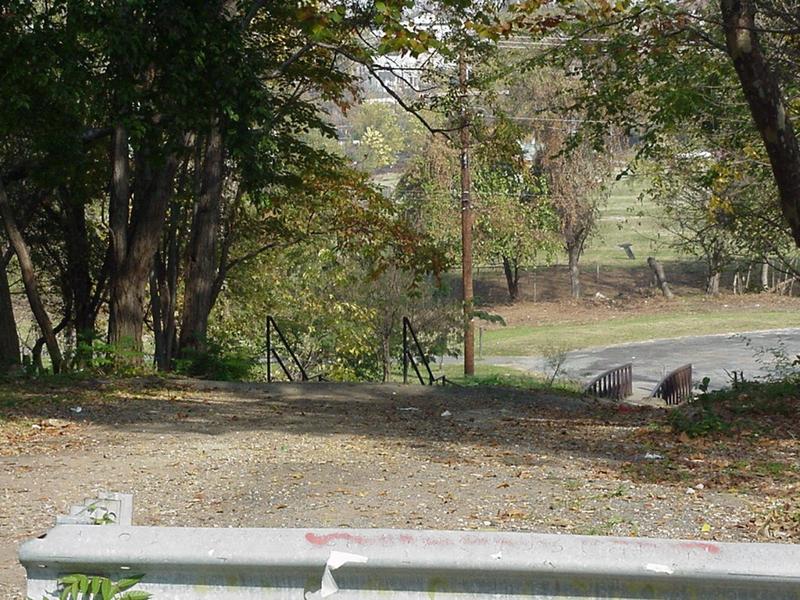

Supplement: S2 Data — Images used in our study. (ZIP) [file pone.0114572.s002.zip › Stimuli/MDS600X800/MDS199.jpg]

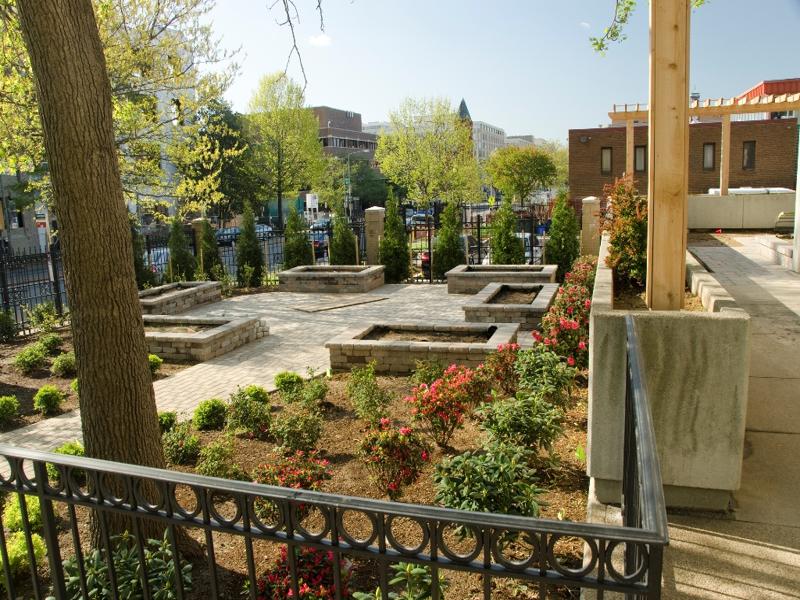

Supplement: S2 Data — Images used in our study. (ZIP) [file pone.0114572.s002.zip › Stimuli/MDS600X800/MDS2.jpg]

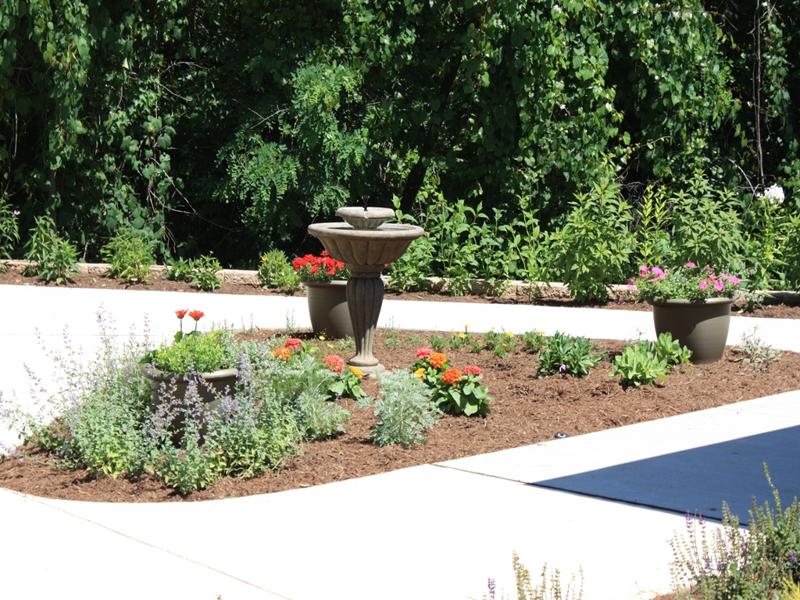

Supplement: S2 Data — Images used in our study. (ZIP) [file pone.0114572.s002.zip › Stimuli/MDS600X800/MDS20.jpg]

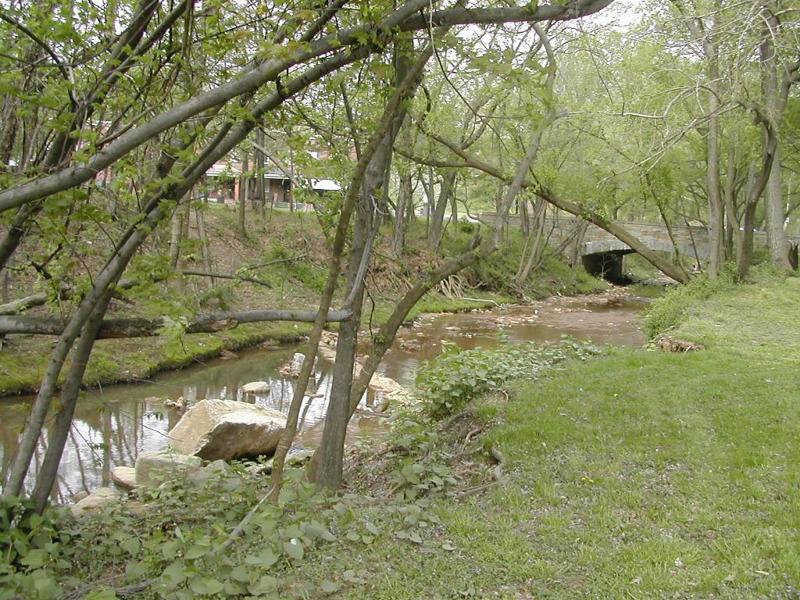

Supplement: S2 Data — Images used in our study. (ZIP) [file pone.0114572.s002.zip › Stimuli/MDS600X800/MDS200.jpg]

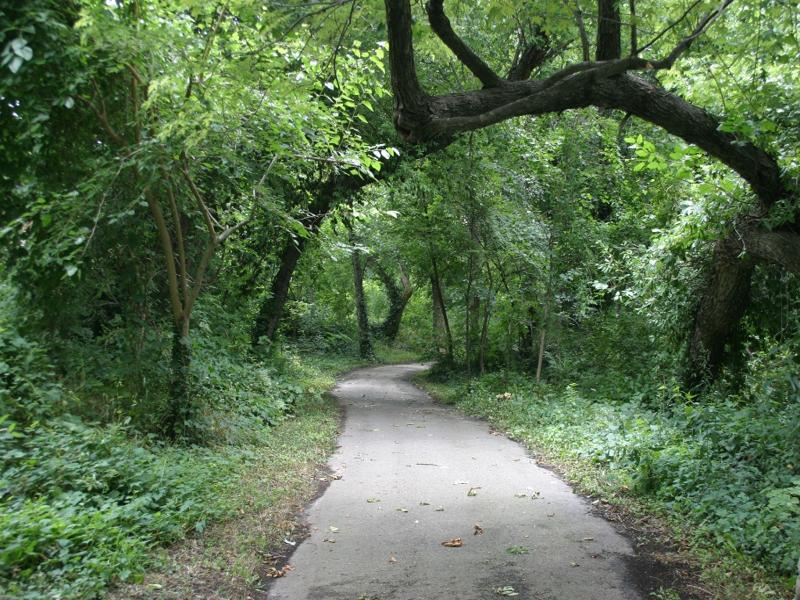

Supplement: S2 Data — Images used in our study. (ZIP) [file pone.0114572.s002.zip › Stimuli/MDS600X800/MDS201.jpg]

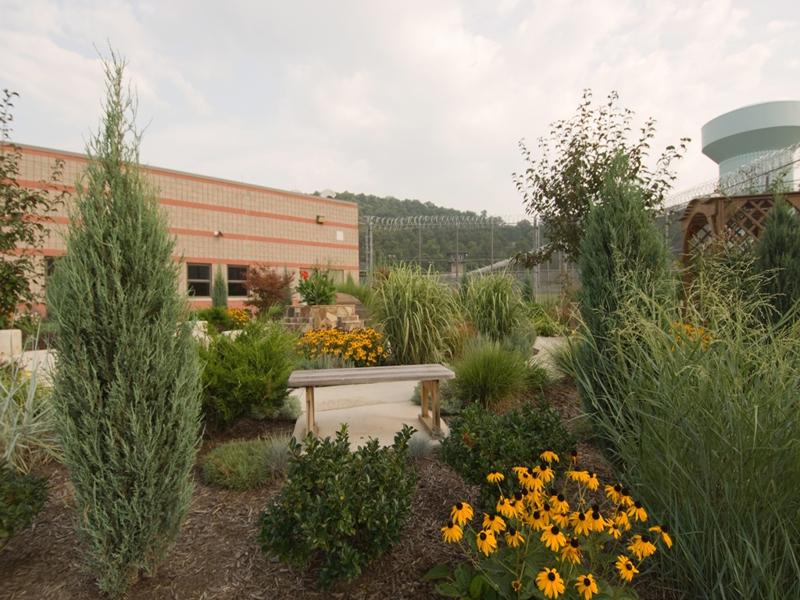

Supplement: S2 Data — Images used in our study. (ZIP) [file pone.0114572.s002.zip › Stimuli/MDS600X800/MDS202.jpg]

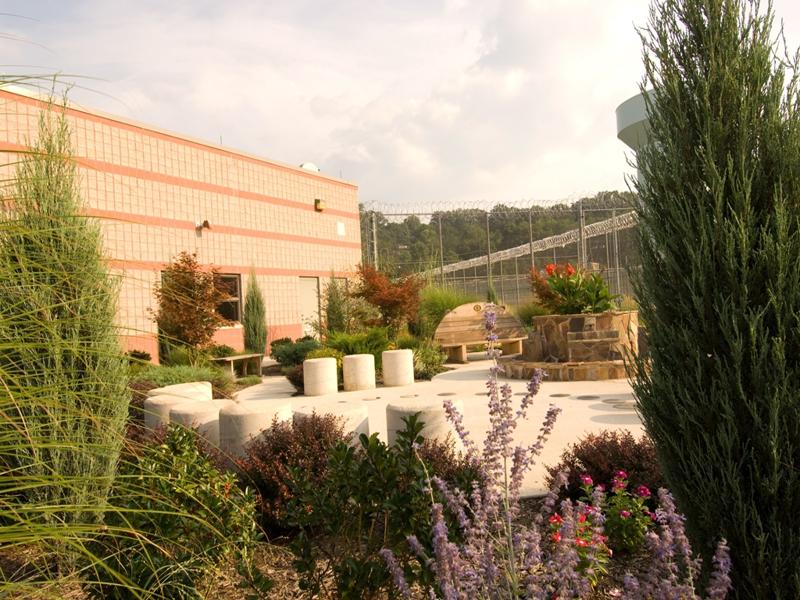

Supplement: S2 Data — Images used in our study. (ZIP) [file pone.0114572.s002.zip › Stimuli/MDS600X800/MDS203.jpg]

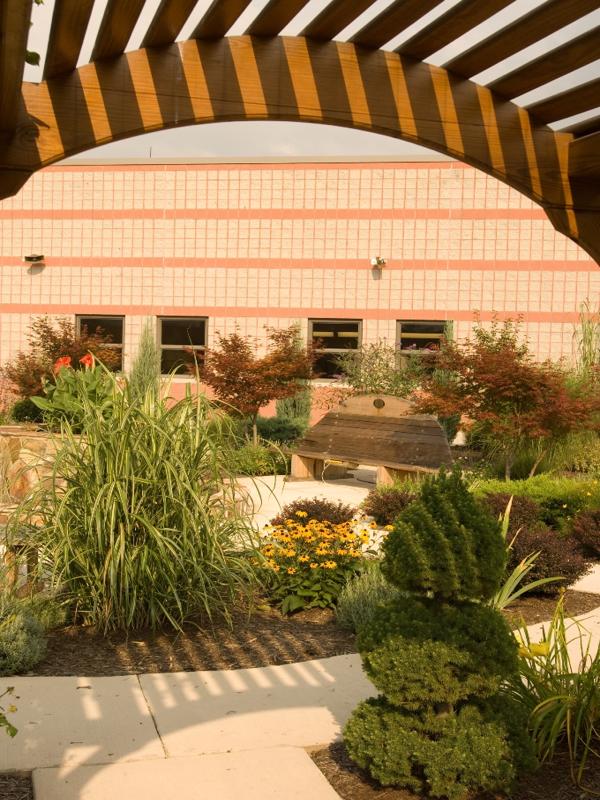

Supplement: S2 Data — Images used in our study. (ZIP) [file pone.0114572.s002.zip › Stimuli/MDS600X800/MDS204.jpg]

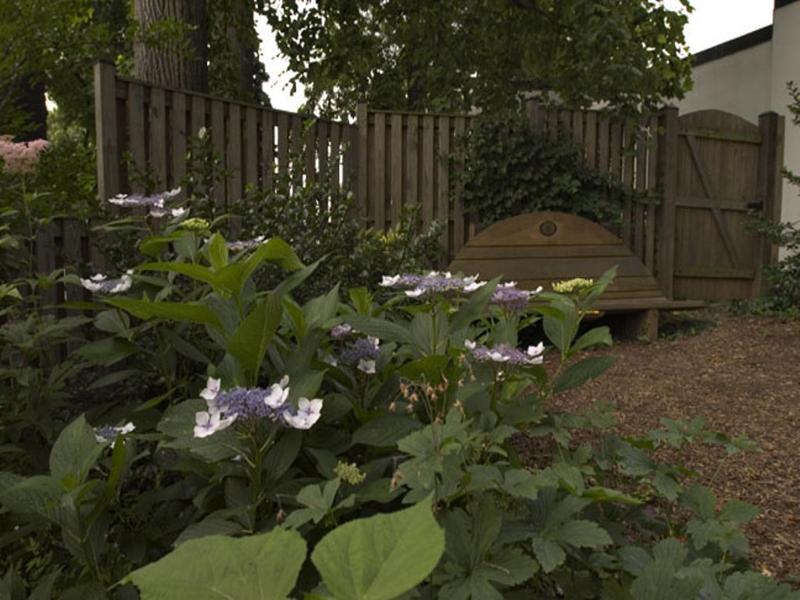

Supplement: S2 Data — Images used in our study. (ZIP) [file pone.0114572.s002.zip › Stimuli/MDS600X800/MDS205.jpg]

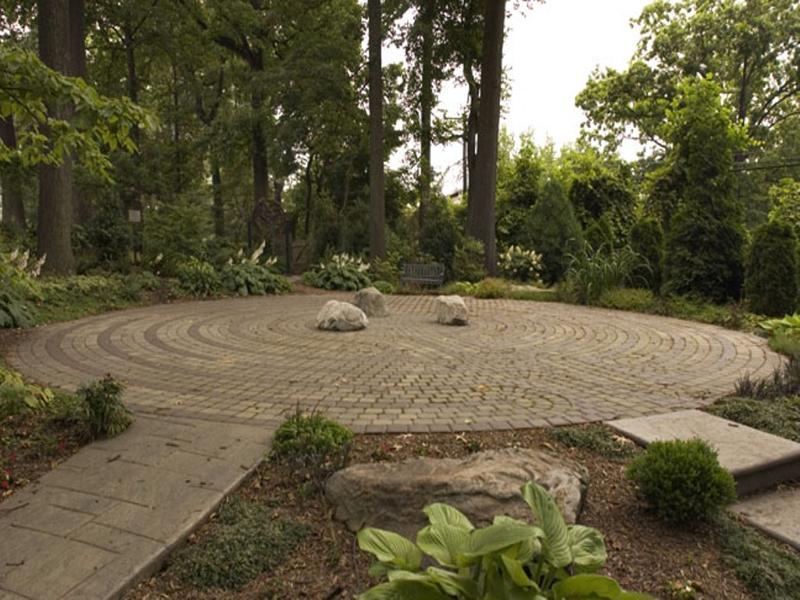

Supplement: S2 Data — Images used in our study. (ZIP) [file pone.0114572.s002.zip › Stimuli/MDS600X800/MDS206.jpg]

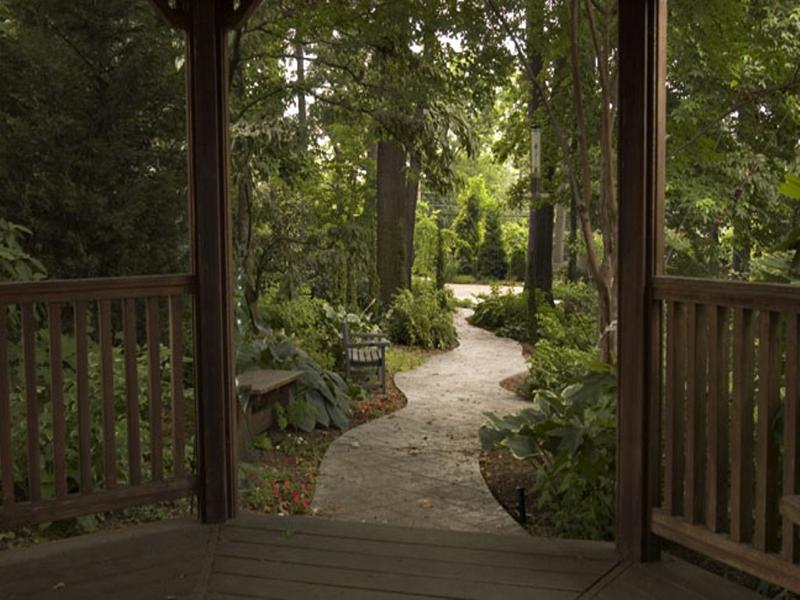

Supplement: S2 Data — Images used in our study. (ZIP) [file pone.0114572.s002.zip › Stimuli/MDS600X800/MDS207.jpg]

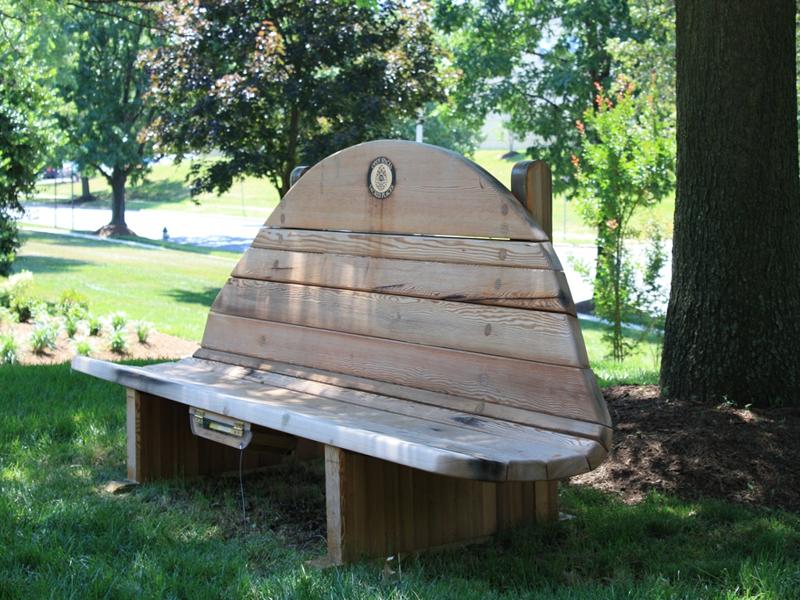

Supplement: S2 Data — Images used in our study. (ZIP) [file pone.0114572.s002.zip › Stimuli/MDS600X800/MDS21.jpg]

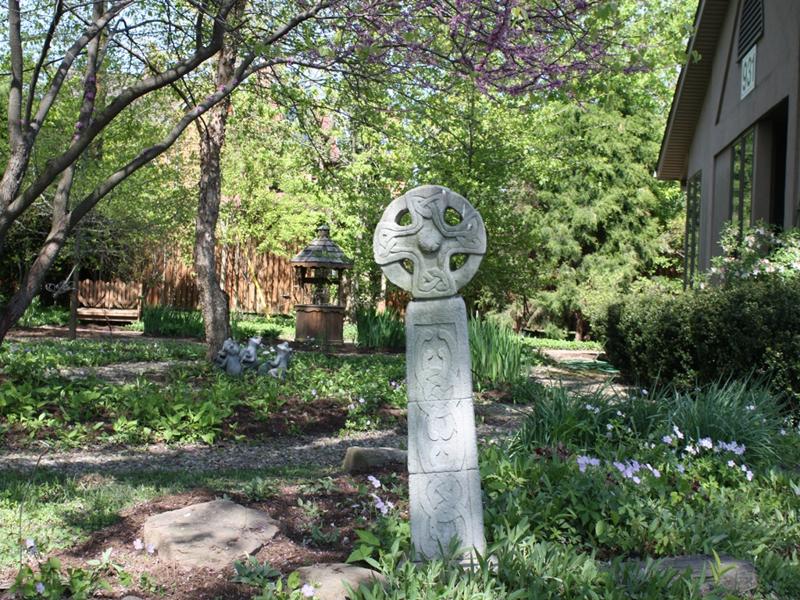

Supplement: S2 Data — Images used in our study. (ZIP) [file pone.0114572.s002.zip › Stimuli/MDS600X800/MDS22.jpg]

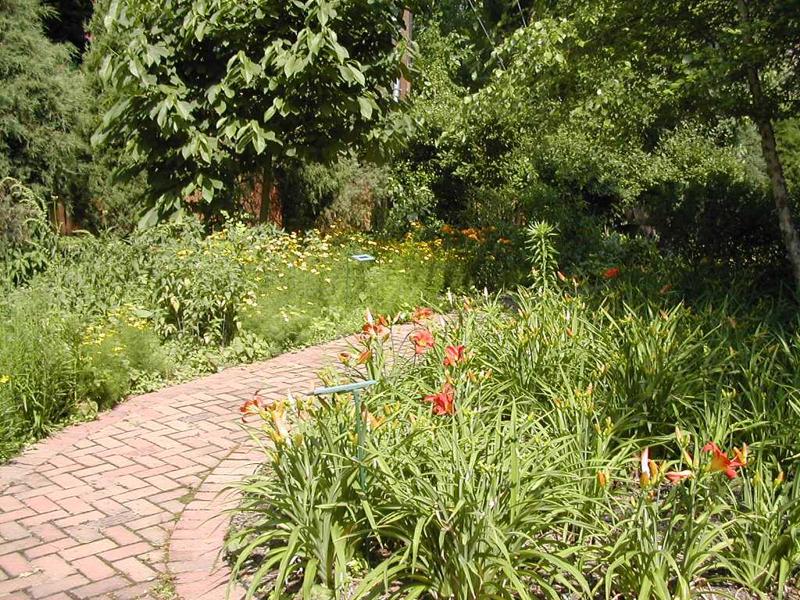

Supplement: S2 Data — Images used in our study. (ZIP) [file pone.0114572.s002.zip › Stimuli/MDS600X800/MDS23.jpg]

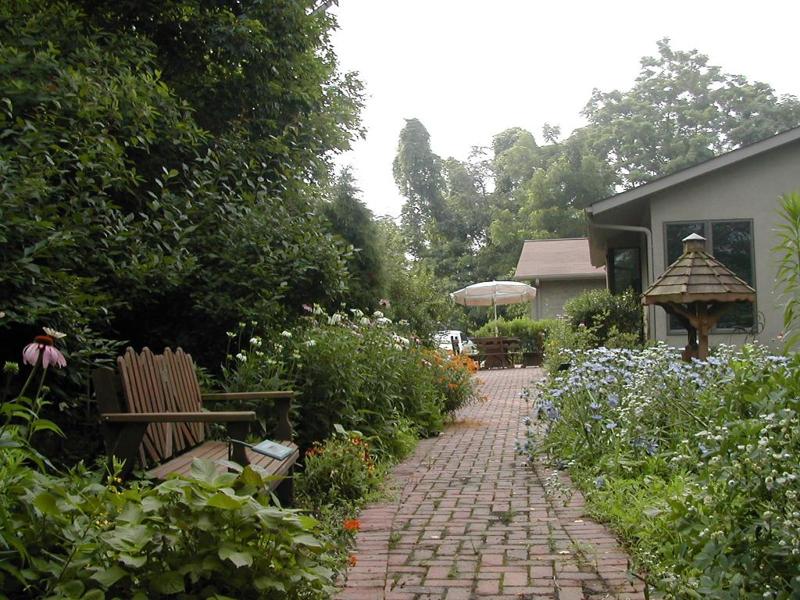

Supplement: S2 Data — Images used in our study. (ZIP) [file pone.0114572.s002.zip › Stimuli/MDS600X800/MDS24.jpg]

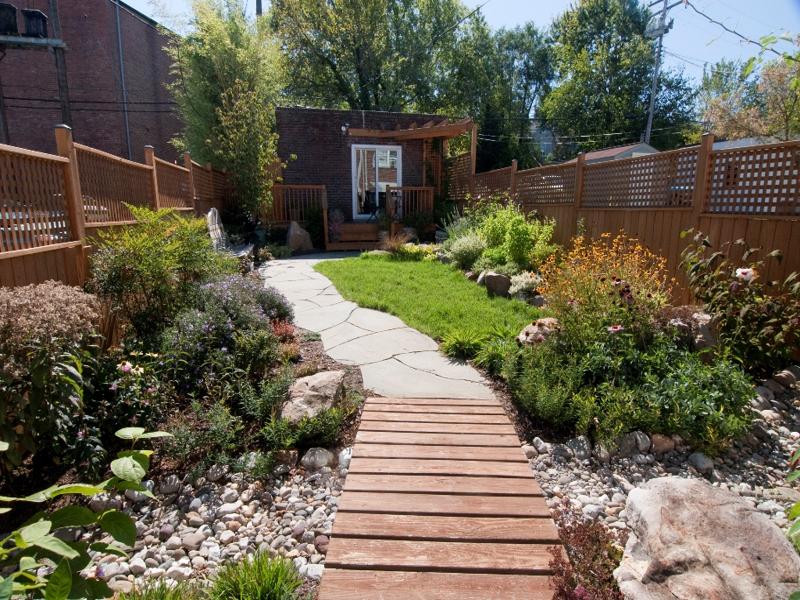

Supplement: S2 Data — Images used in our study. (ZIP) [file pone.0114572.s002.zip › Stimuli/MDS600X800/MDS25.jpg]

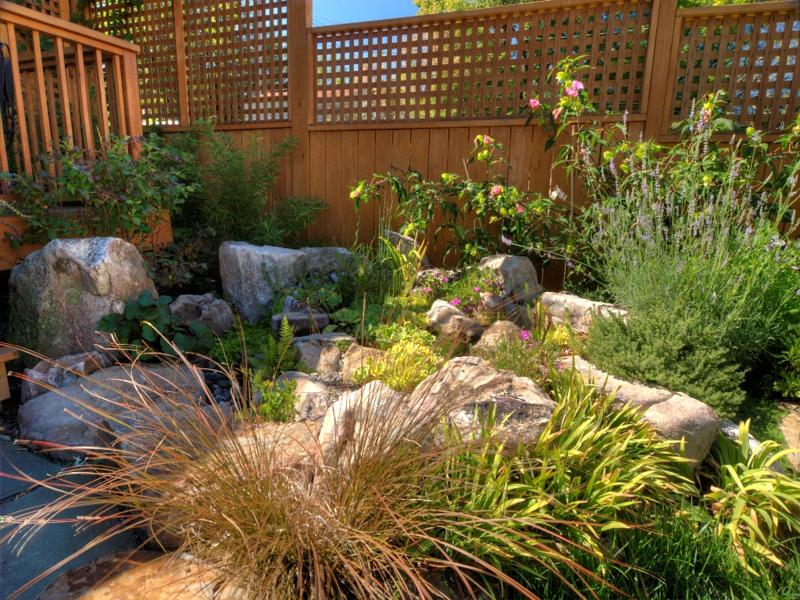

Supplement: S2 Data — Images used in our study. (ZIP) [file pone.0114572.s002.zip › Stimuli/MDS600X800/MDS26.jpg]

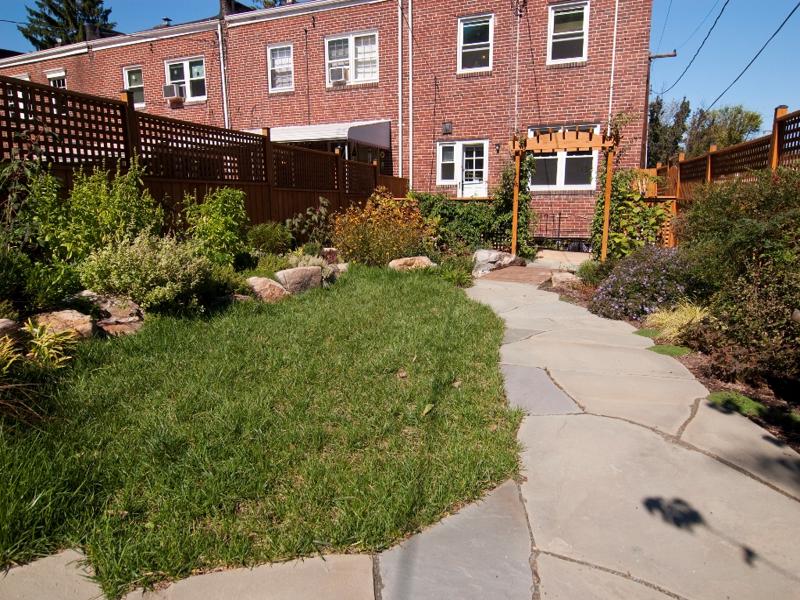

Supplement: S2 Data — Images used in our study. (ZIP) [file pone.0114572.s002.zip › Stimuli/MDS600X800/MDS27.jpg]

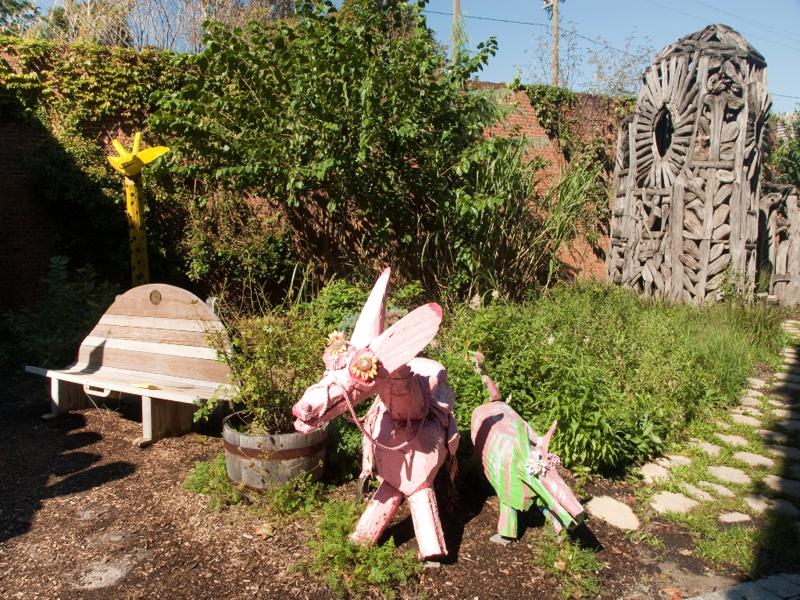

Supplement: S2 Data — Images used in our study. (ZIP) [file pone.0114572.s002.zip › Stimuli/MDS600X800/MDS28.jpg]

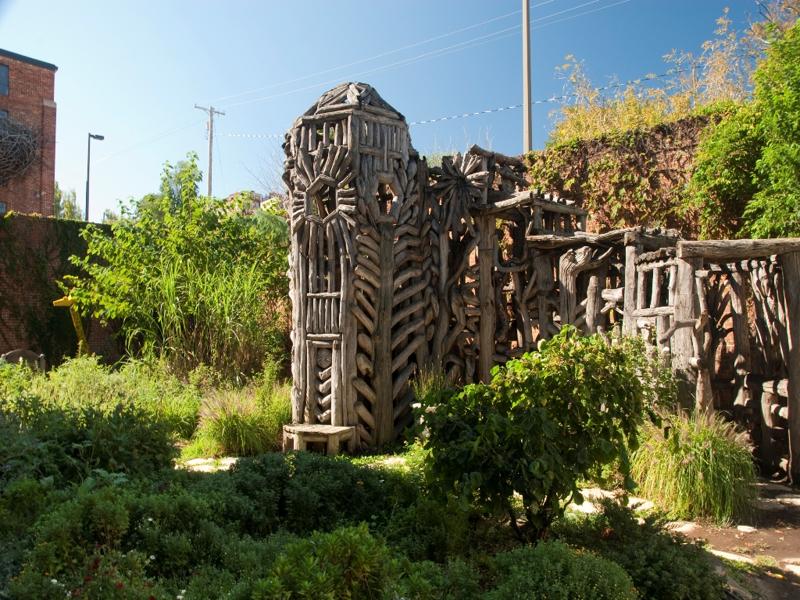

Supplement: S2 Data — Images used in our study. (ZIP) [file pone.0114572.s002.zip › Stimuli/MDS600X800/MDS29.jpg]

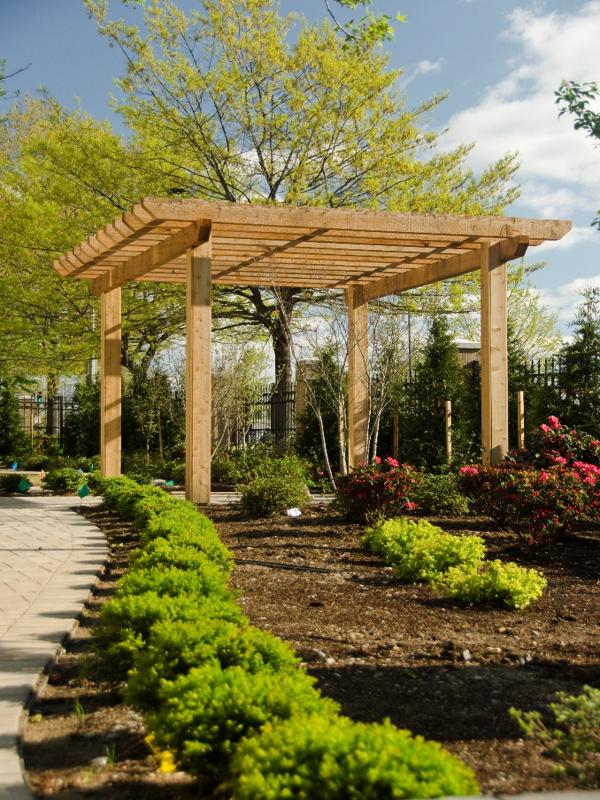

Supplement: S2 Data — Images used in our study. (ZIP) [file pone.0114572.s002.zip › Stimuli/MDS600X800/MDS3.jpg]

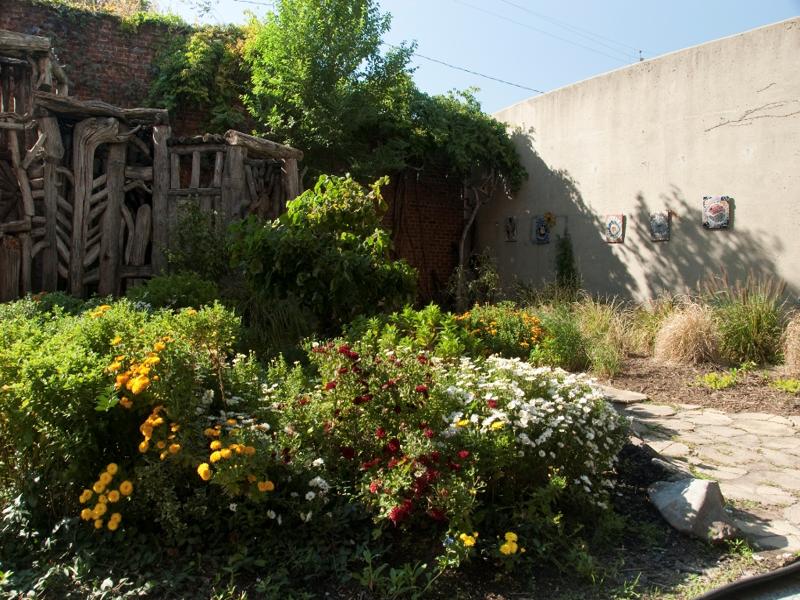

Supplement: S2 Data — Images used in our study. (ZIP) [file pone.0114572.s002.zip › Stimuli/MDS600X800/MDS30.jpg]

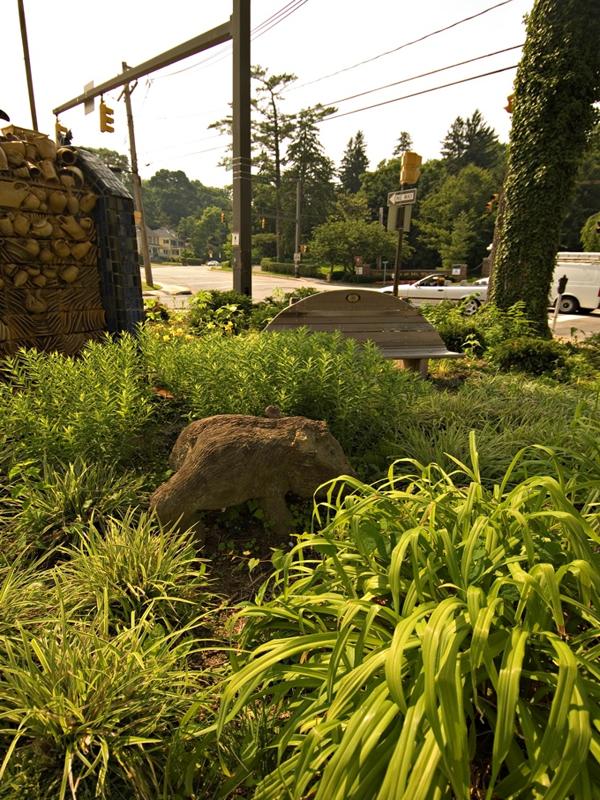

Supplement: S2 Data — Images used in our study. (ZIP) [file pone.0114572.s002.zip › Stimuli/MDS600X800/MDS31.jpg]

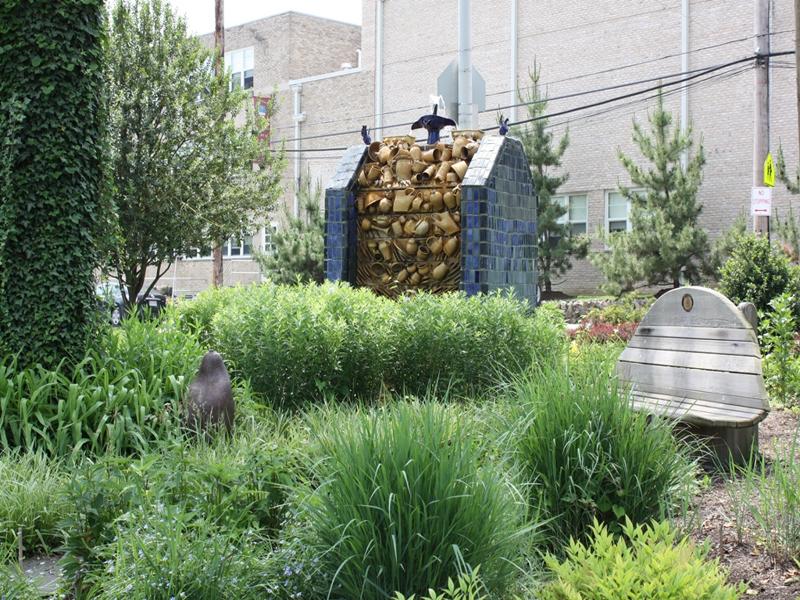

Supplement: S2 Data — Images used in our study. (ZIP) [file pone.0114572.s002.zip › Stimuli/MDS600X800/MDS32.jpg]

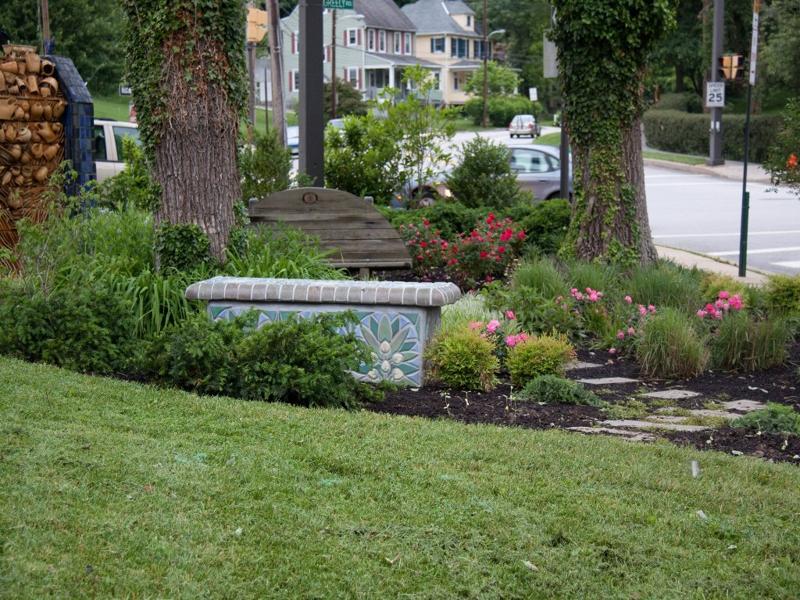

Supplement: S2 Data — Images used in our study. (ZIP) [file pone.0114572.s002.zip › Stimuli/MDS600X800/MDS33.jpg]

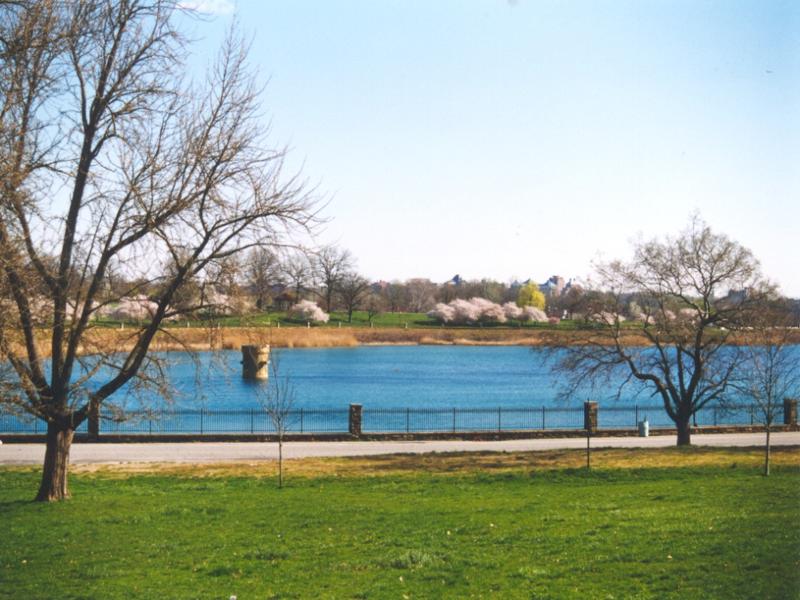

Supplement: S2 Data — Images used in our study. (ZIP) [file pone.0114572.s002.zip › Stimuli/MDS600X800/MDS34.jpg]

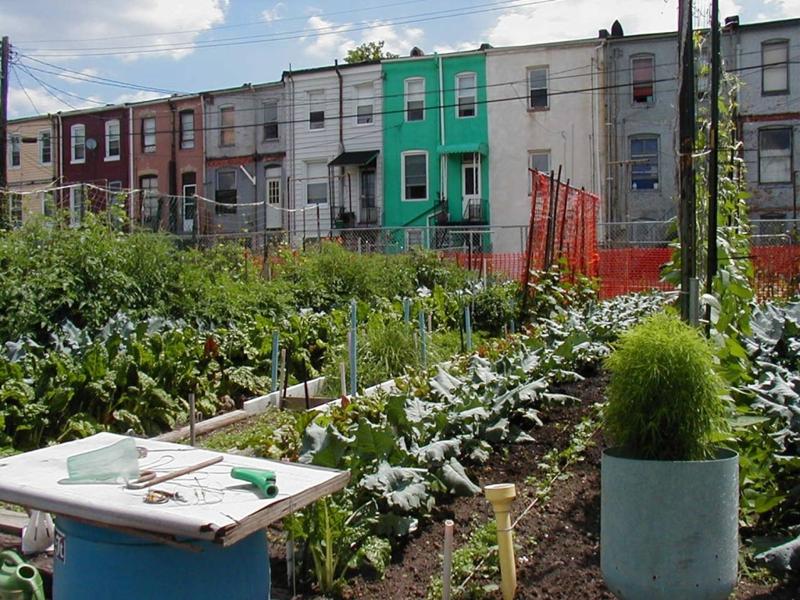

Supplement: S2 Data — Images used in our study. (ZIP) [file pone.0114572.s002.zip › Stimuli/MDS600X800/MDS35.jpg]

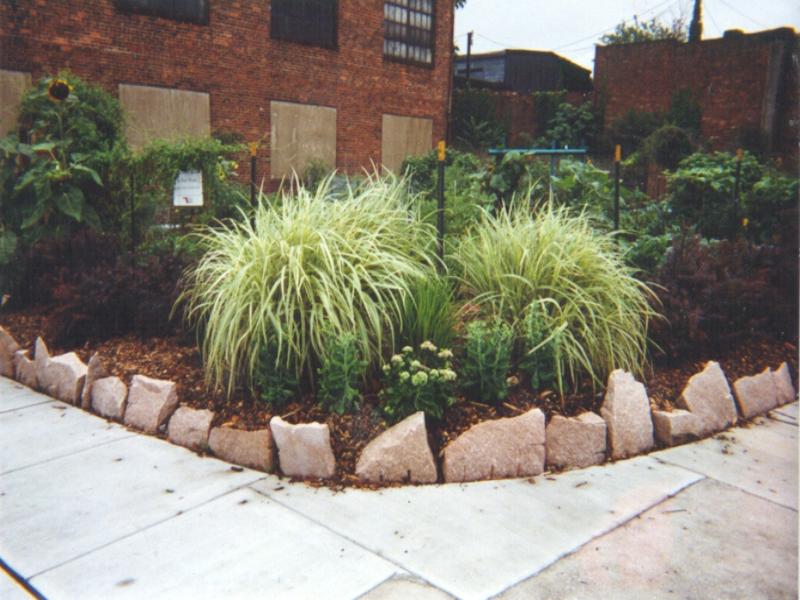

Supplement: S2 Data — Images used in our study. (ZIP) [file pone.0114572.s002.zip › Stimuli/MDS600X800/MDS36.jpg]

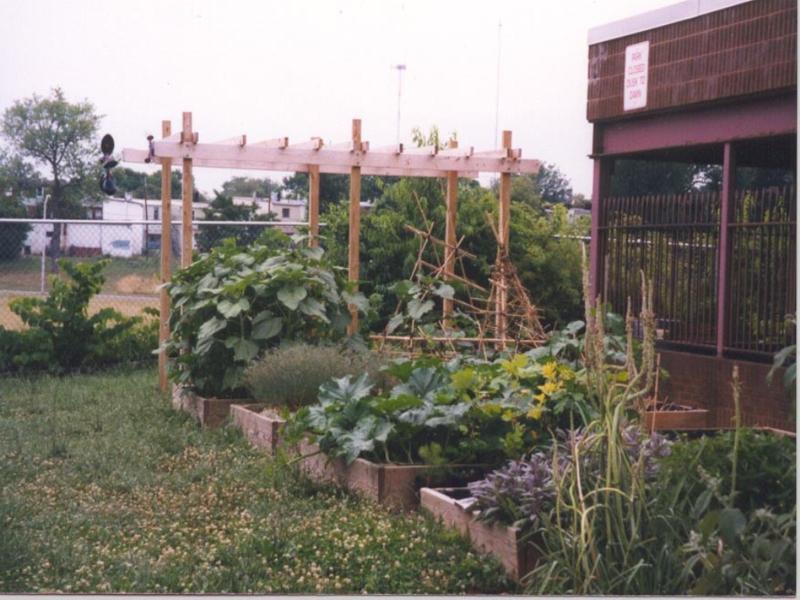

Supplement: S2 Data — Images used in our study. (ZIP) [file pone.0114572.s002.zip › Stimuli/MDS600X800/MDS37.jpg]

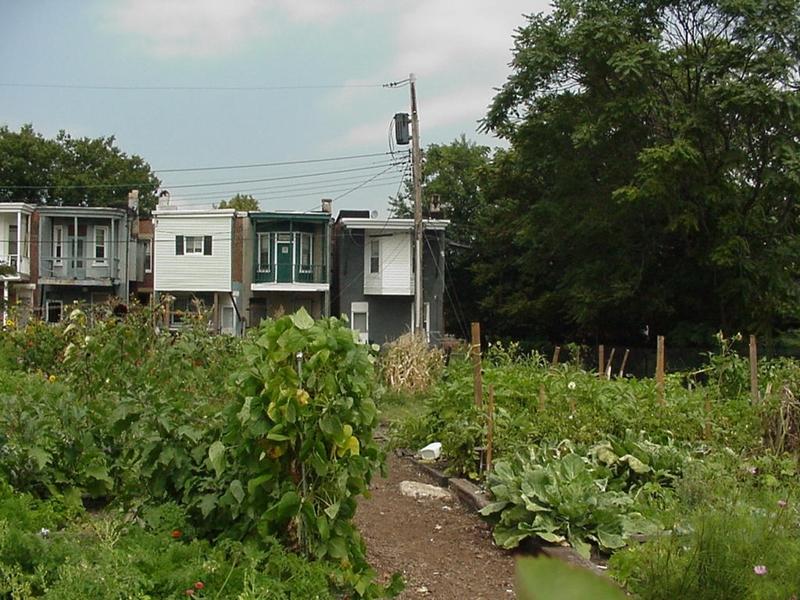

Supplement: S2 Data — Images used in our study. (ZIP) [file pone.0114572.s002.zip › Stimuli/MDS600X800/MDS38.jpg]

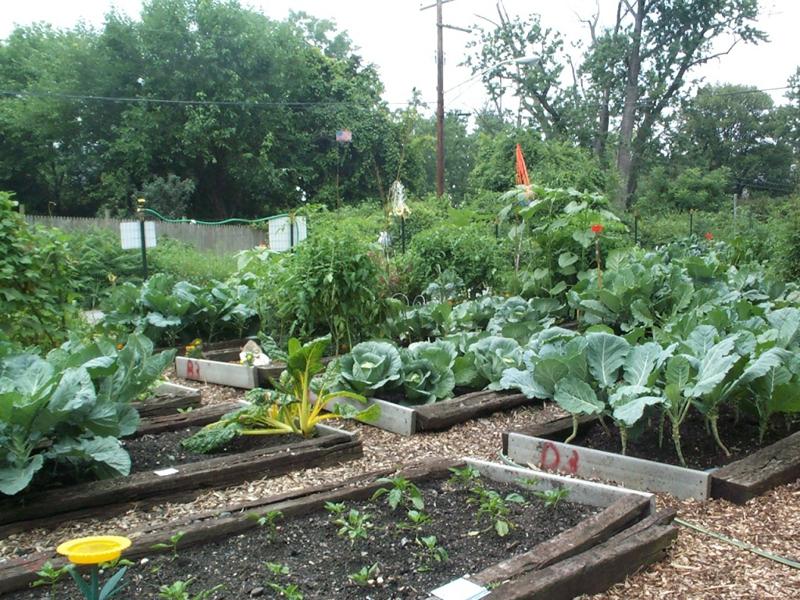

Supplement: S2 Data — Images used in our study. (ZIP) [file pone.0114572.s002.zip › Stimuli/MDS600X800/MDS39.jpg]

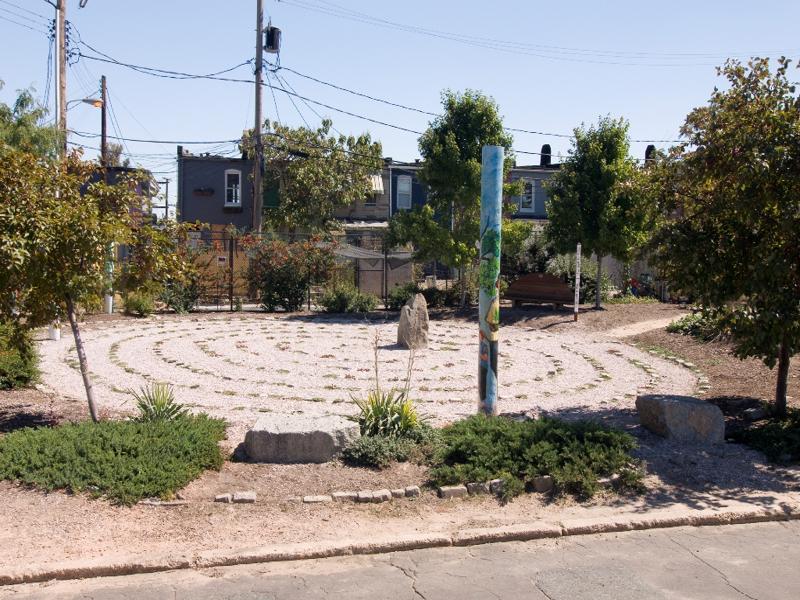

Supplement: S2 Data — Images used in our study. (ZIP) [file pone.0114572.s002.zip › Stimuli/MDS600X800/MDS4.jpg]

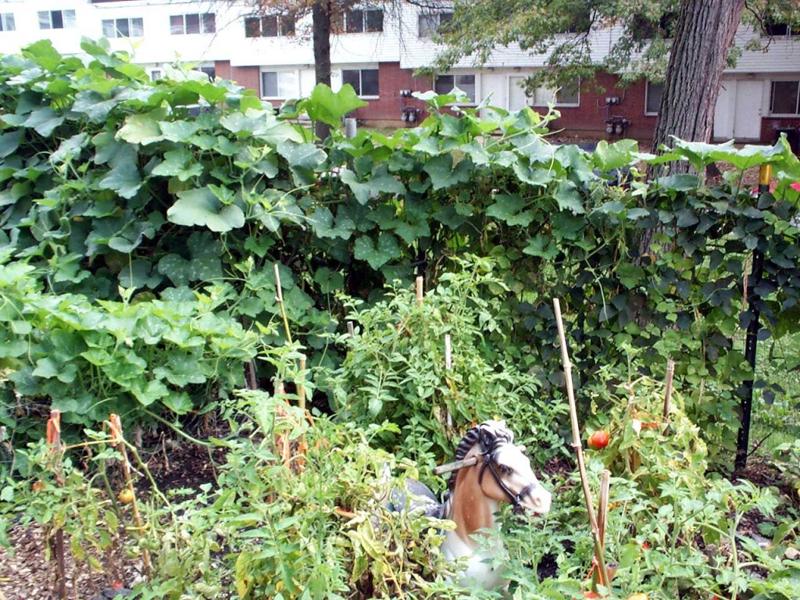

Supplement: S2 Data — Images used in our study. (ZIP) [file pone.0114572.s002.zip › Stimuli/MDS600X800/MDS40.jpg]

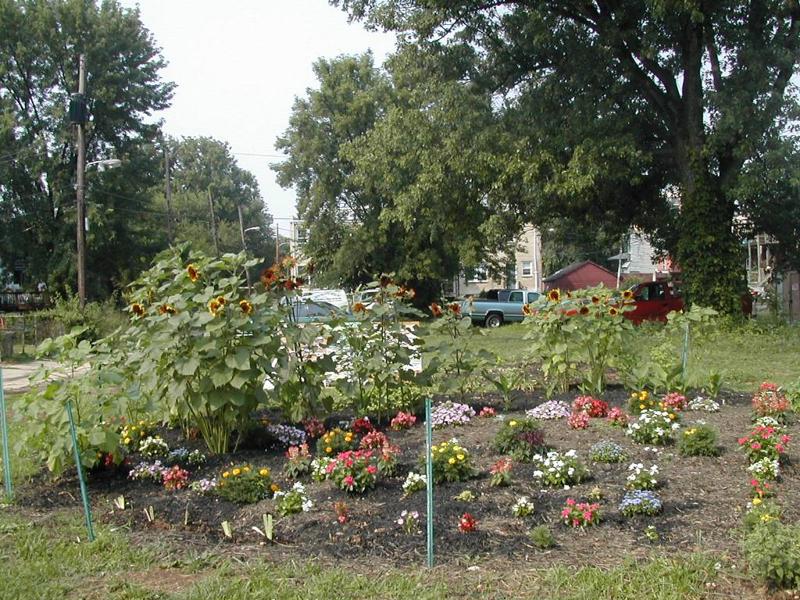

Supplement: S2 Data — Images used in our study. (ZIP) [file pone.0114572.s002.zip › Stimuli/MDS600X800/MDS41.jpg]

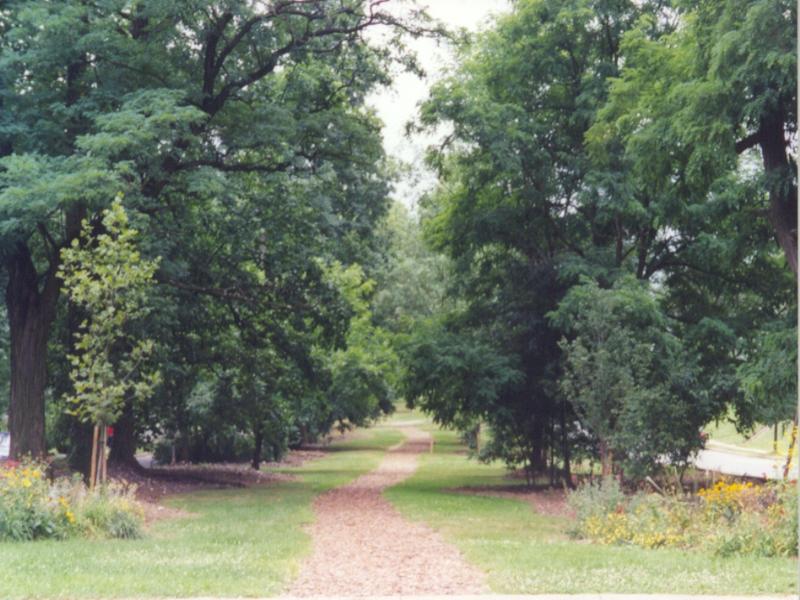

Supplement: S2 Data — Images used in our study. (ZIP) [file pone.0114572.s002.zip › Stimuli/MDS600X800/MDS42.jpg]

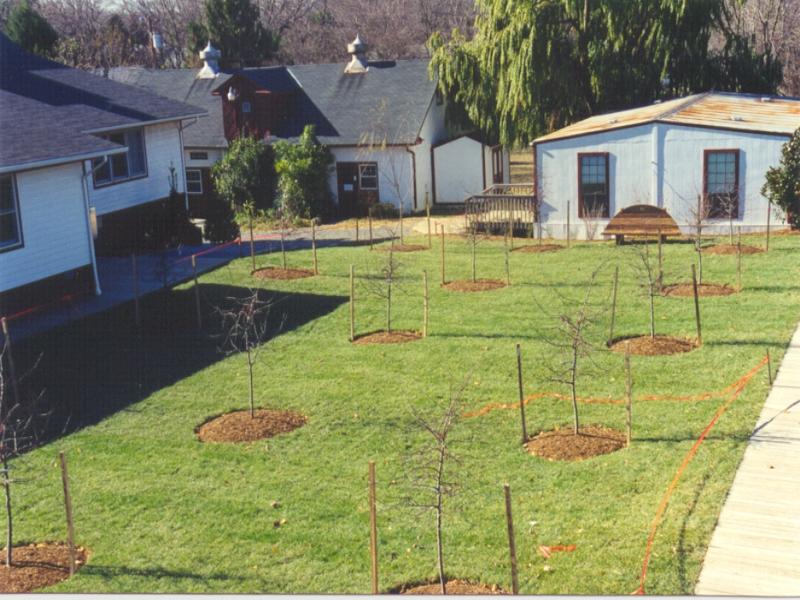

Supplement: S2 Data — Images used in our study. (ZIP) [file pone.0114572.s002.zip › Stimuli/MDS600X800/MDS43.jpg]

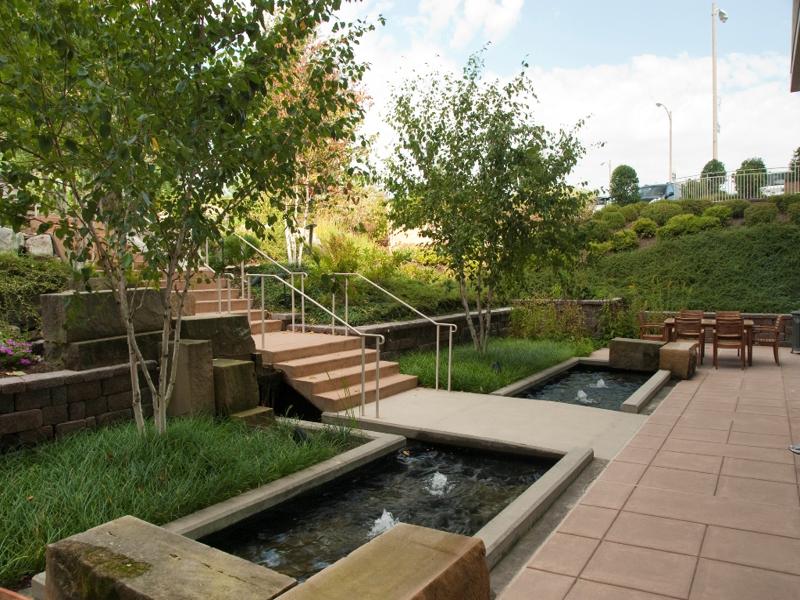

Supplement: S2 Data — Images used in our study. (ZIP) [file pone.0114572.s002.zip › Stimuli/MDS600X800/MDS44.jpg]

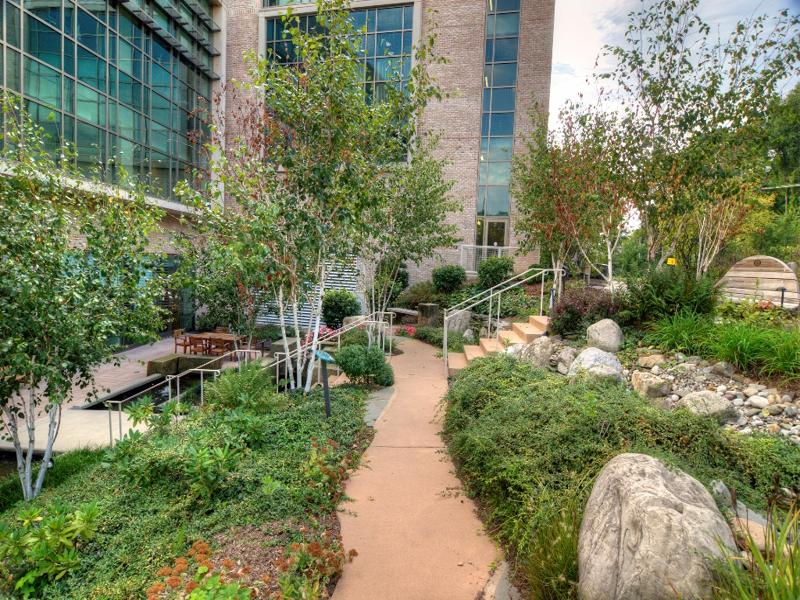

Supplement: S2 Data — Images used in our study. (ZIP) [file pone.0114572.s002.zip › Stimuli/MDS600X800/MDS45.jpg]

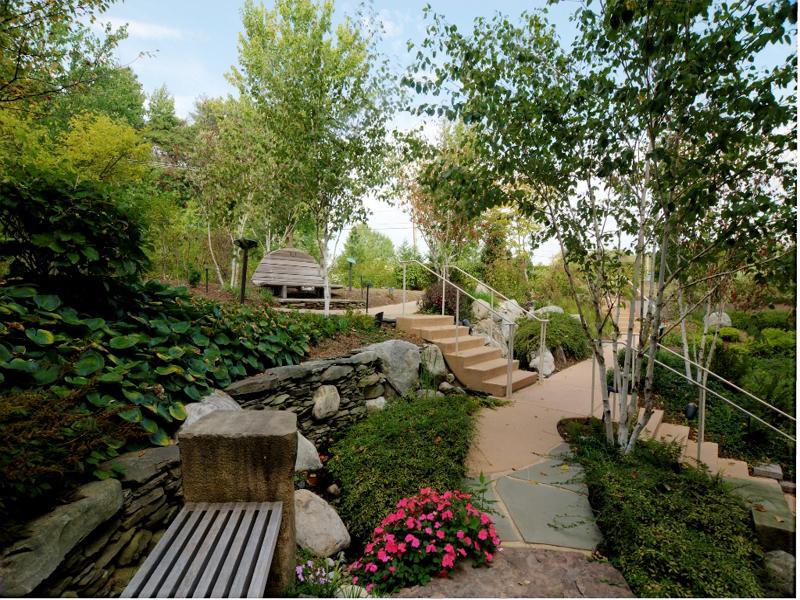

Supplement: S2 Data — Images used in our study. (ZIP) [file pone.0114572.s002.zip › Stimuli/MDS600X800/MDS46.jpg]

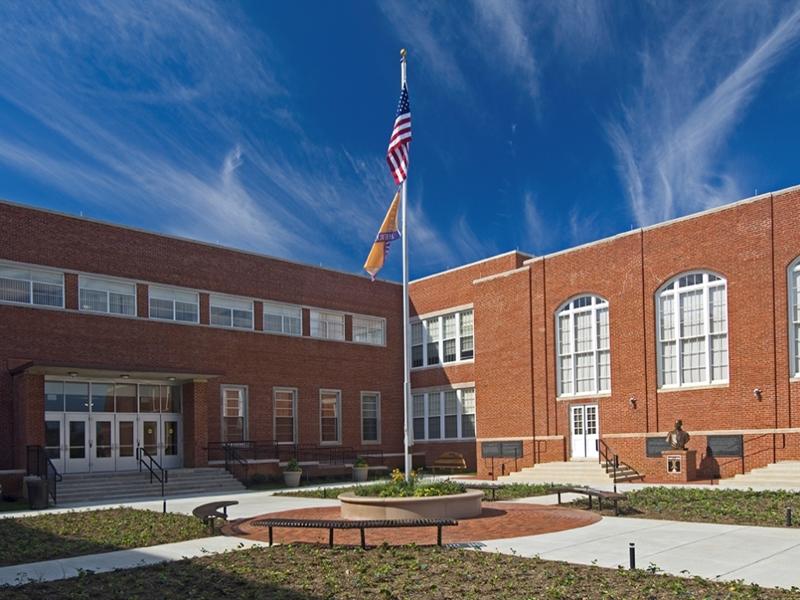

Supplement: S2 Data — Images used in our study. (ZIP) [file pone.0114572.s002.zip › Stimuli/MDS600X800/MDS47.jpg]

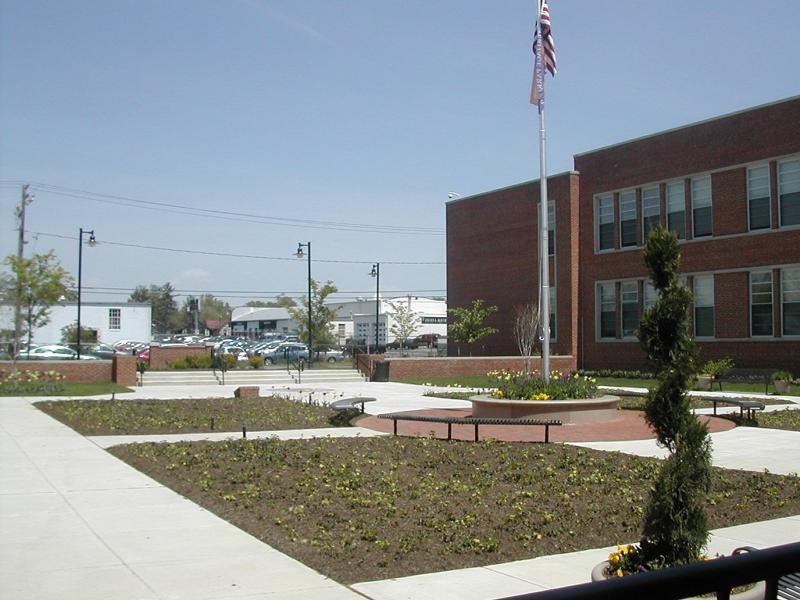

Supplement: S2 Data — Images used in our study. (ZIP) [file pone.0114572.s002.zip › Stimuli/MDS600X800/MDS48.jpg]

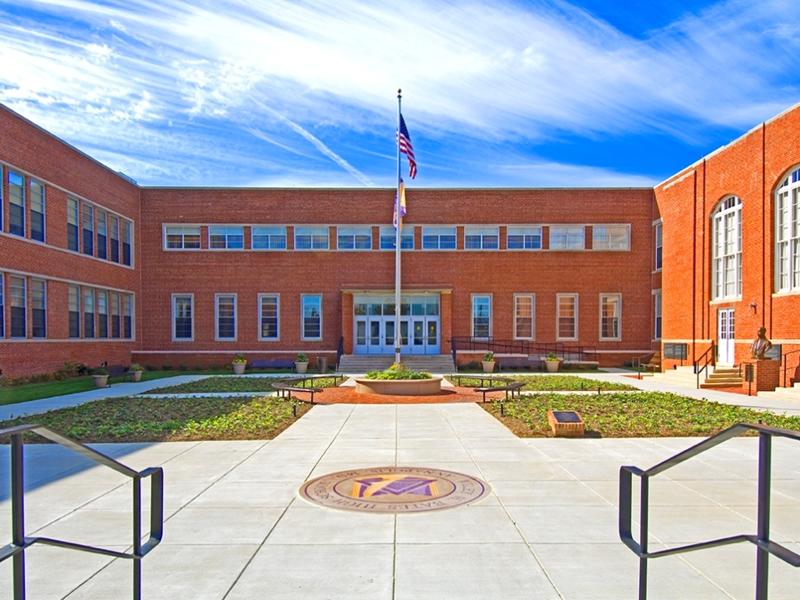

Supplement: S2 Data — Images used in our study. (ZIP) [file pone.0114572.s002.zip › Stimuli/MDS600X800/MDS49.jpg]

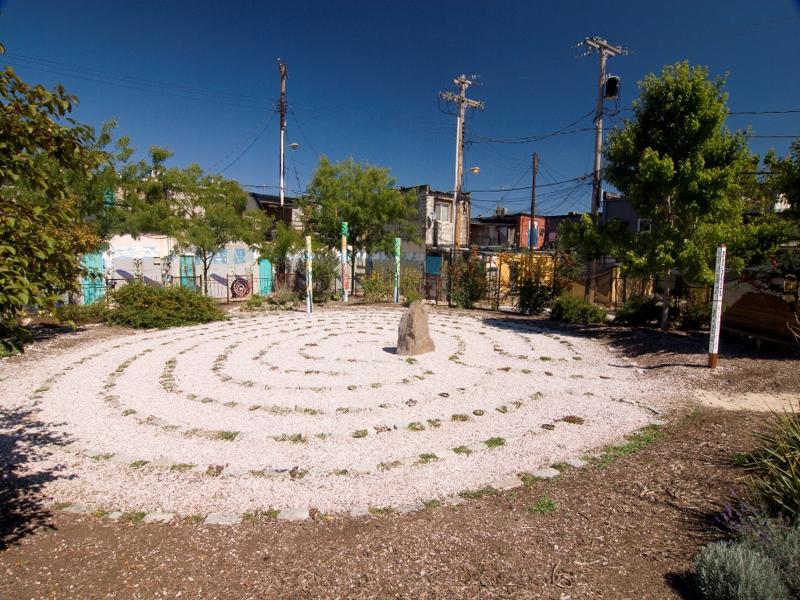

Supplement: S2 Data — Images used in our study. (ZIP) [file pone.0114572.s002.zip › Stimuli/MDS600X800/MDS5.jpg]

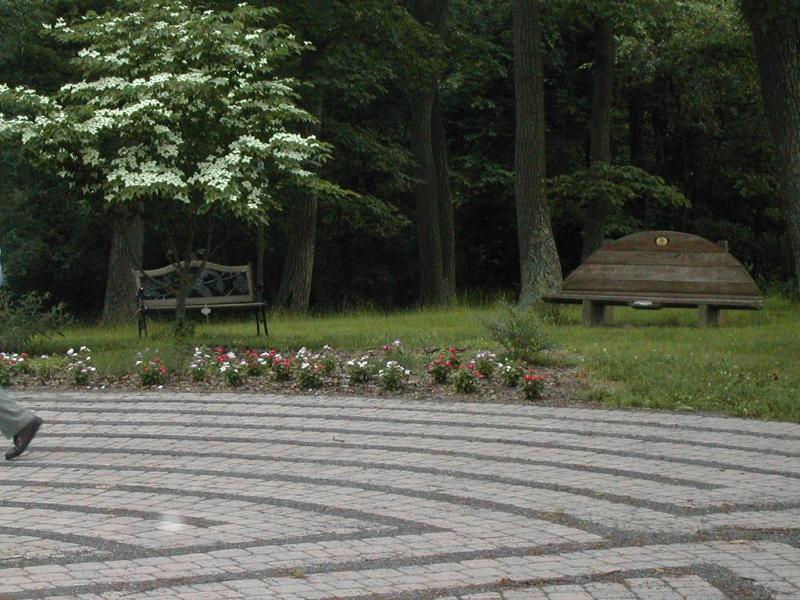

Supplement: S2 Data — Images used in our study. (ZIP) [file pone.0114572.s002.zip › Stimuli/MDS600X800/MDS50.jpg]

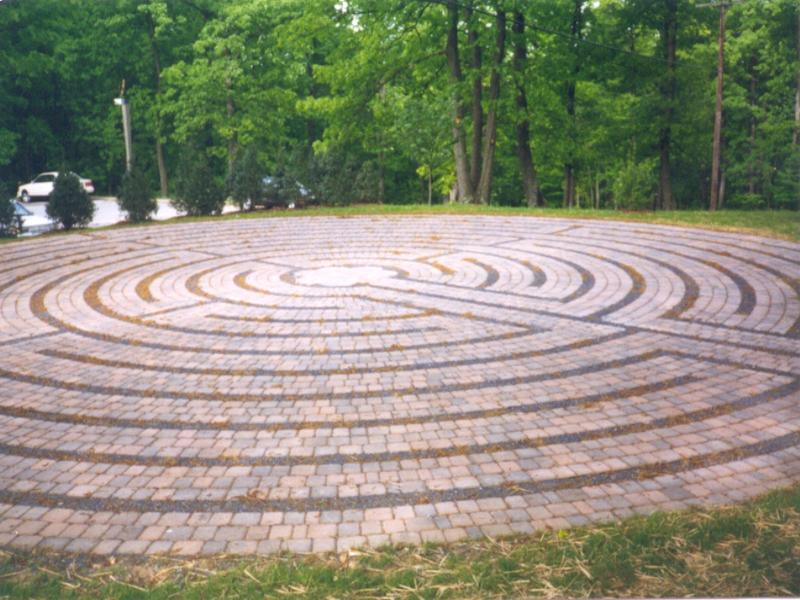

Supplement: S2 Data — Images used in our study. (ZIP) [file pone.0114572.s002.zip › Stimuli/MDS600X800/MDS51.jpg]

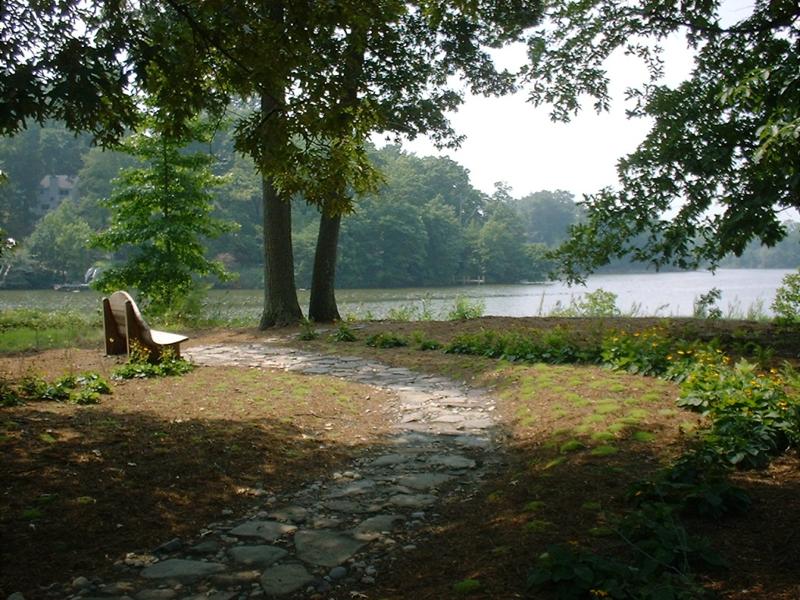

Supplement: S2 Data — Images used in our study. (ZIP) [file pone.0114572.s002.zip › Stimuli/MDS600X800/MDS52.jpg]

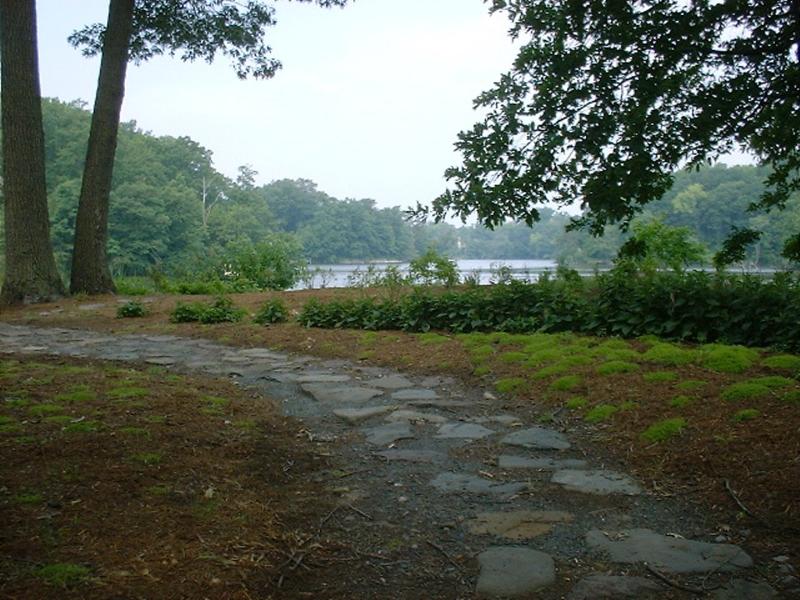

Supplement: S2 Data — Images used in our study. (ZIP) [file pone.0114572.s002.zip › Stimuli/MDS600X800/MDS53.jpg]

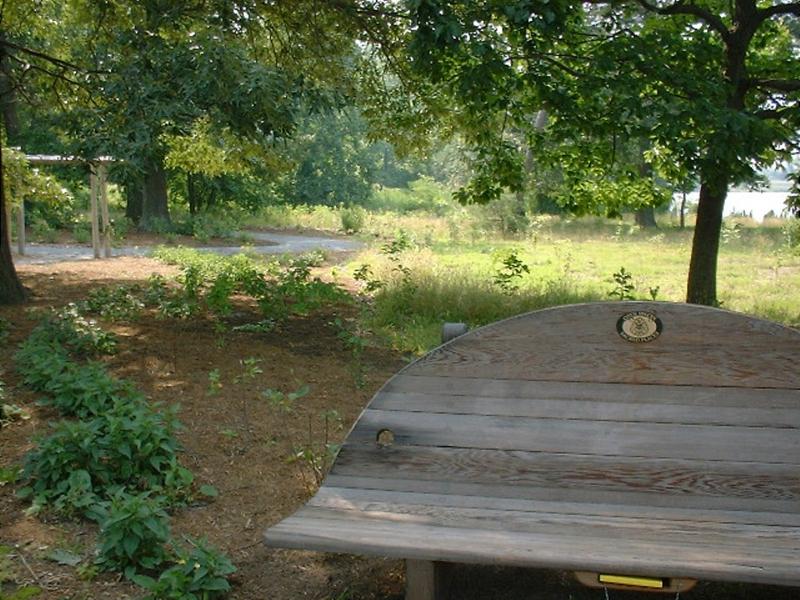

Supplement: S2 Data — Images used in our study. (ZIP) [file pone.0114572.s002.zip › Stimuli/MDS600X800/MDS54.jpg]

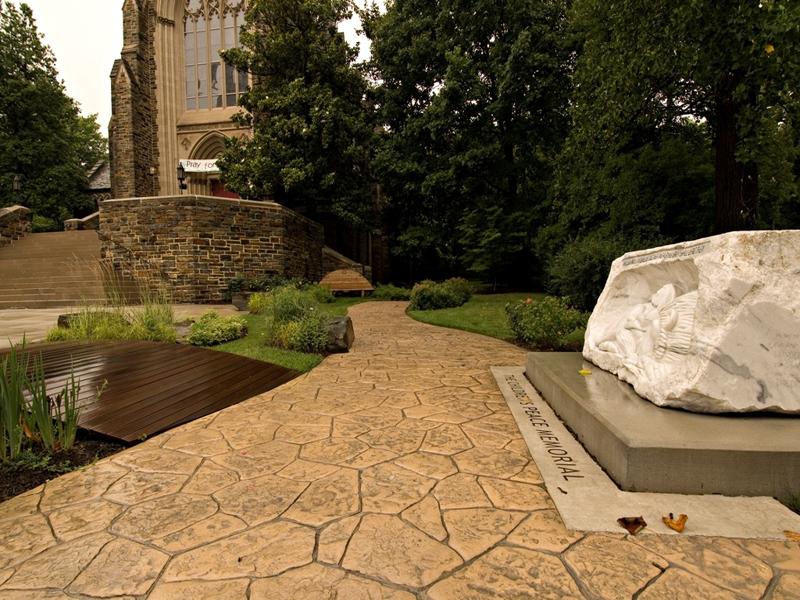

Supplement: S2 Data — Images used in our study. (ZIP) [file pone.0114572.s002.zip › Stimuli/MDS600X800/MDS55.jpg]

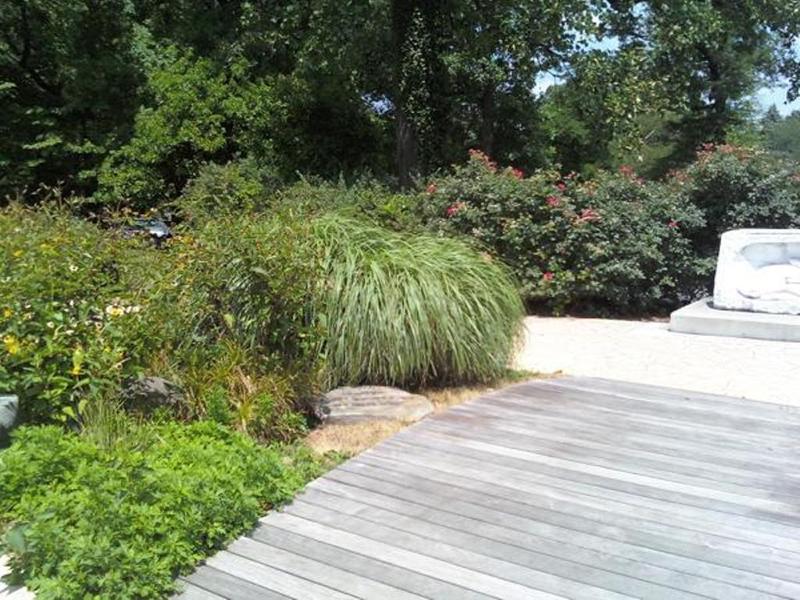

Supplement: S2 Data — Images used in our study. (ZIP) [file pone.0114572.s002.zip › Stimuli/MDS600X800/MDS56.jpg]

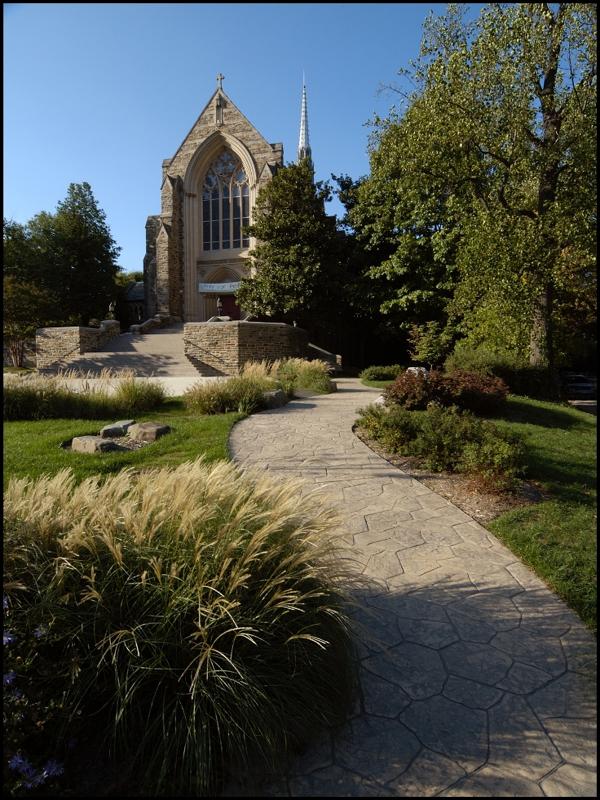

Supplement: S2 Data — Images used in our study. (ZIP) [file pone.0114572.s002.zip › Stimuli/MDS600X800/MDS57.jpg]

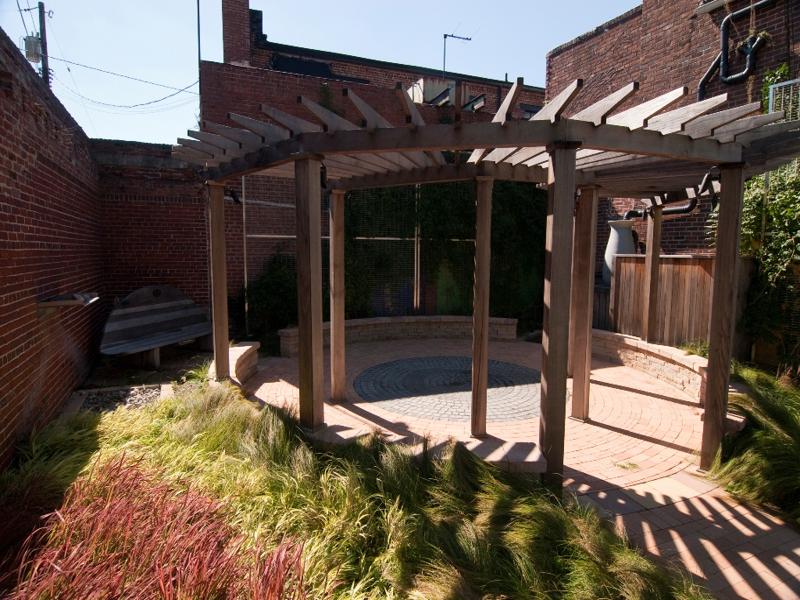

Supplement: S2 Data — Images used in our study. (ZIP) [file pone.0114572.s002.zip › Stimuli/MDS600X800/MDS58.jpg]

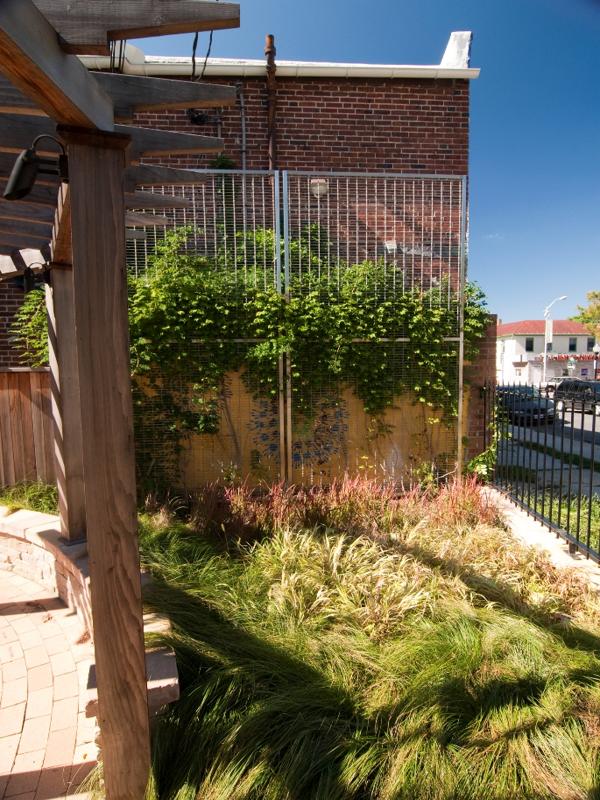

Supplement: S2 Data — Images used in our study. (ZIP) [file pone.0114572.s002.zip › Stimuli/MDS600X800/MDS59.jpg]

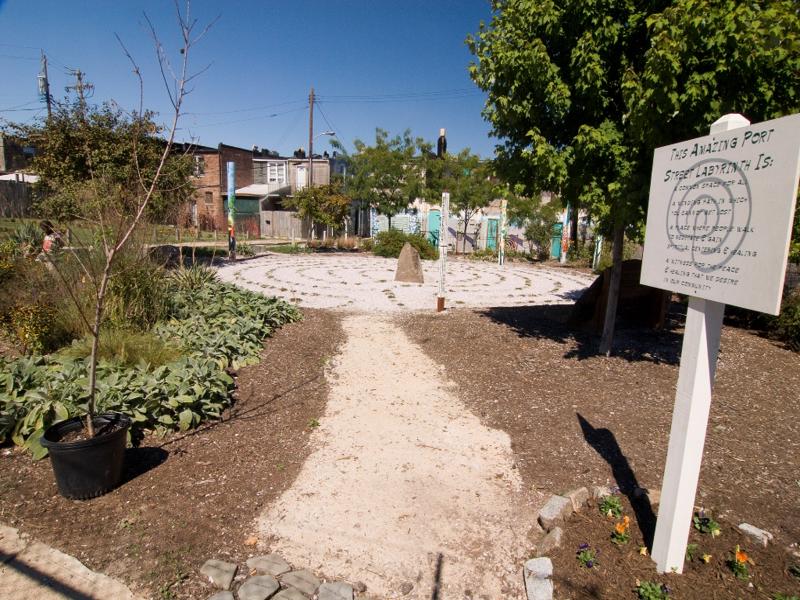

Supplement: S2 Data — Images used in our study. (ZIP) [file pone.0114572.s002.zip › Stimuli/MDS600X800/MDS6.jpg]

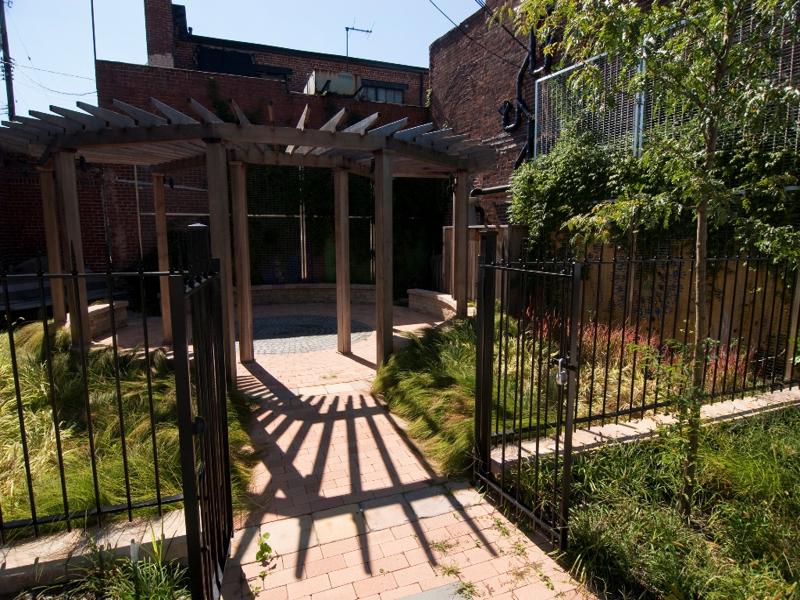

Supplement: S2 Data — Images used in our study. (ZIP) [file pone.0114572.s002.zip › Stimuli/MDS600X800/MDS60.jpg]

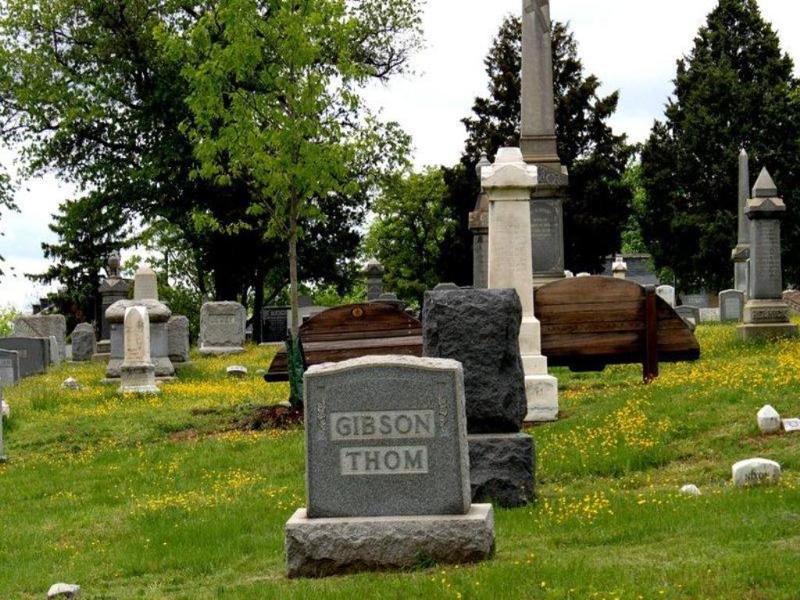

Supplement: S2 Data — Images used in our study. (ZIP) [file pone.0114572.s002.zip › Stimuli/MDS600X800/MDS61.jpg]

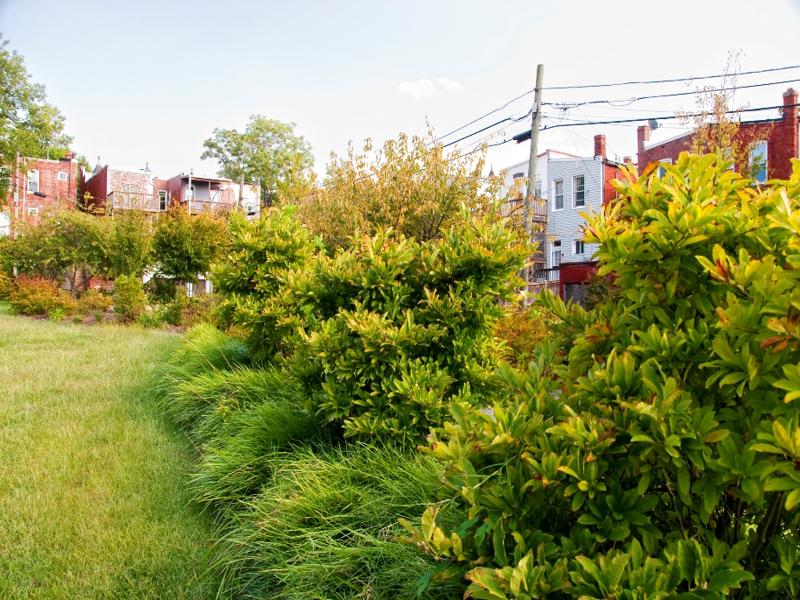

Supplement: S2 Data — Images used in our study. (ZIP) [file pone.0114572.s002.zip › Stimuli/MDS600X800/MDS62.jpg]

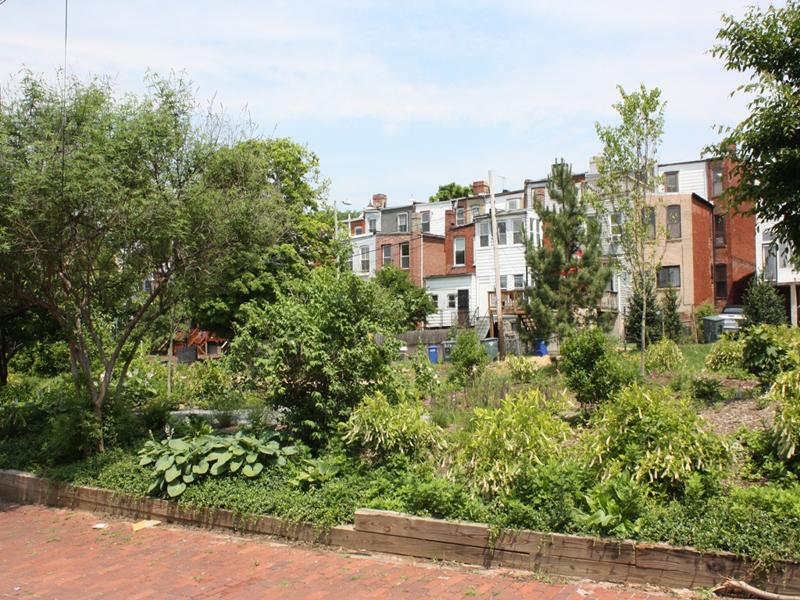

Supplement: S2 Data — Images used in our study. (ZIP) [file pone.0114572.s002.zip › Stimuli/MDS600X800/MDS63.jpg]

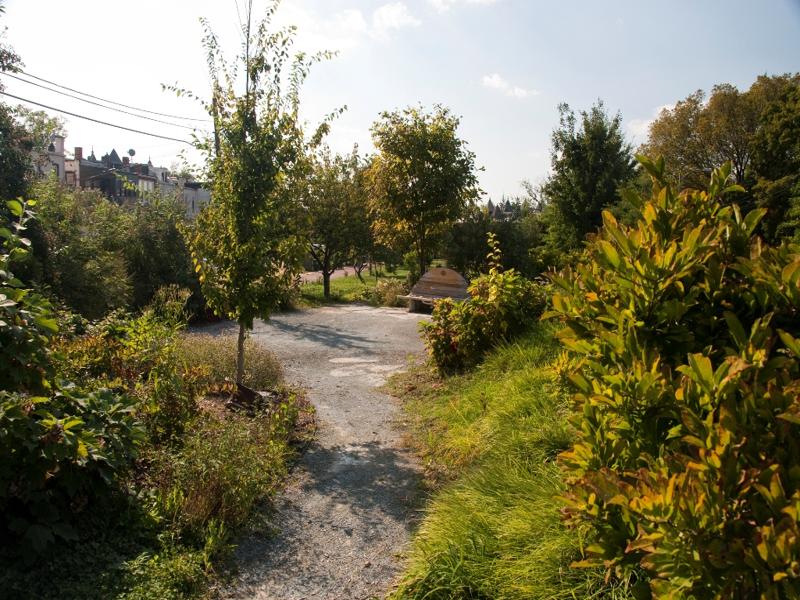

Supplement: S2 Data — Images used in our study. (ZIP) [file pone.0114572.s002.zip › Stimuli/MDS600X800/MDS64.jpg]

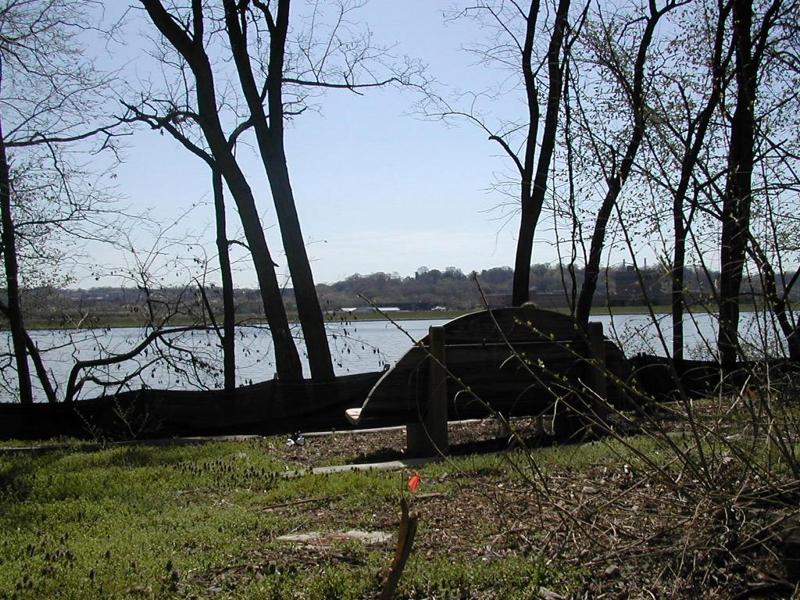

Supplement: S2 Data — Images used in our study. (ZIP) [file pone.0114572.s002.zip › Stimuli/MDS600X800/MDS65.jpg]

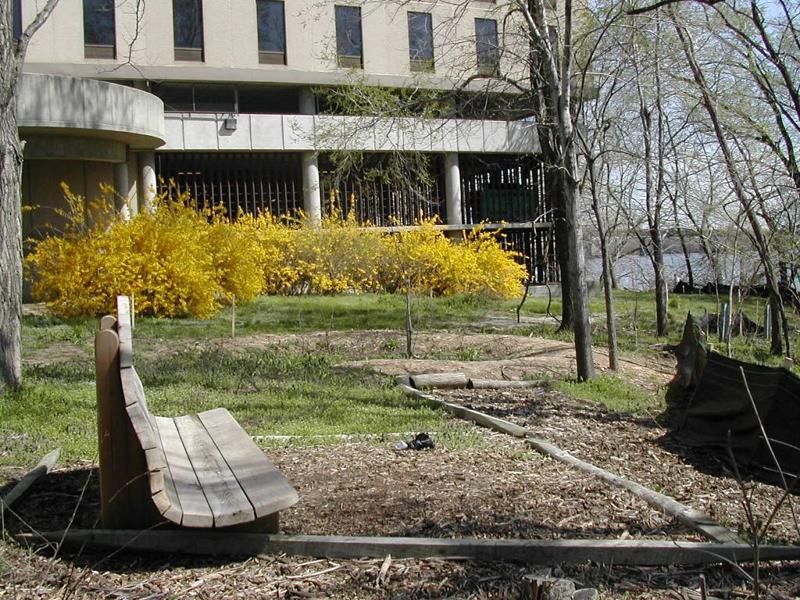

Supplement: S2 Data — Images used in our study. (ZIP) [file pone.0114572.s002.zip › Stimuli/MDS600X800/MDS66.jpg]

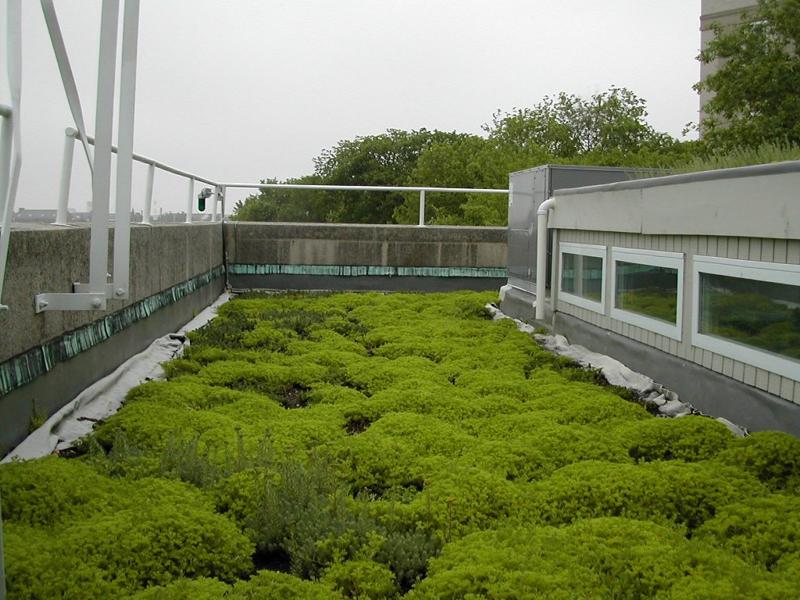

Supplement: S2 Data — Images used in our study. (ZIP) [file pone.0114572.s002.zip › Stimuli/MDS600X800/MDS67.jpg]

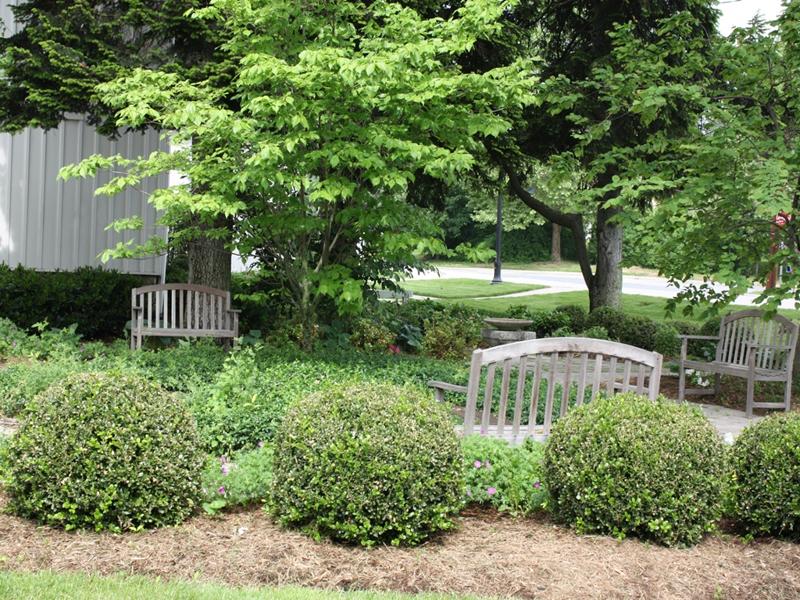

Supplement: S2 Data — Images used in our study. (ZIP) [file pone.0114572.s002.zip › Stimuli/MDS600X800/MDS68.jpg]

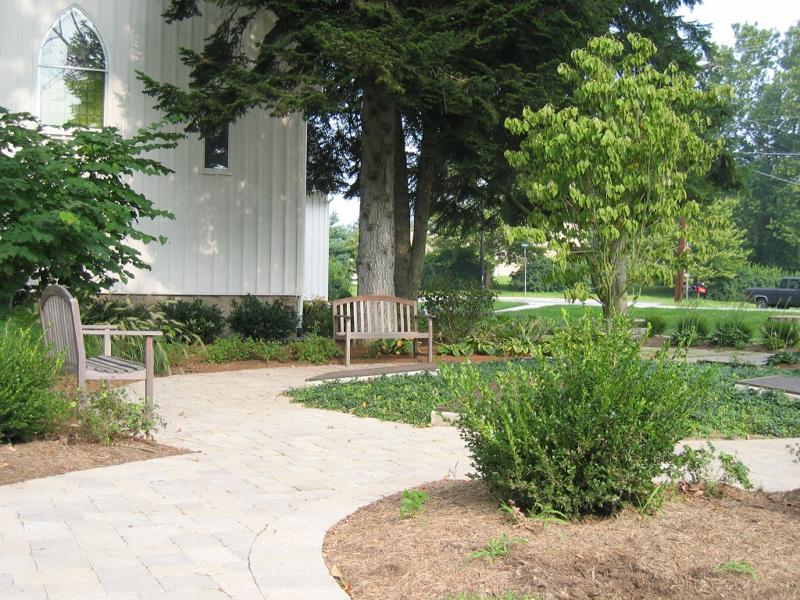

Supplement: S2 Data — Images used in our study. (ZIP) [file pone.0114572.s002.zip › Stimuli/MDS600X800/MDS69.jpg]

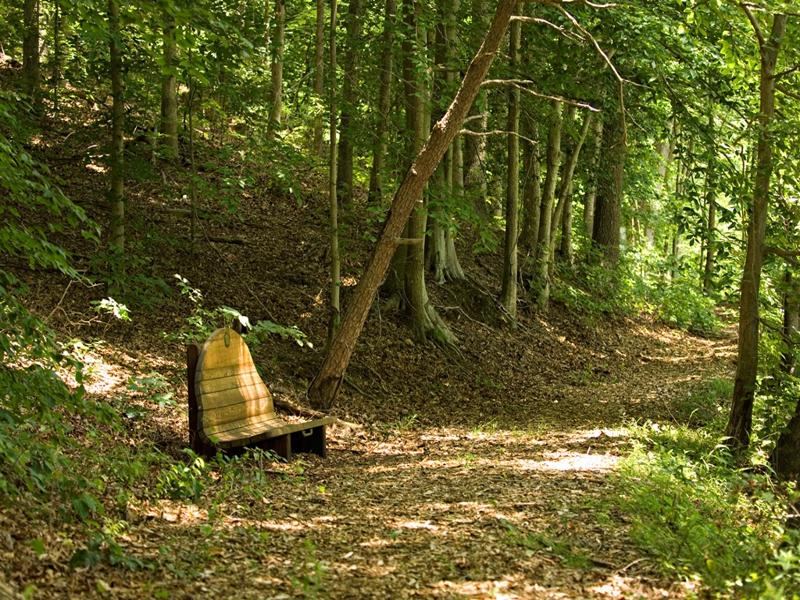

Supplement: S2 Data — Images used in our study. (ZIP) [file pone.0114572.s002.zip › Stimuli/MDS600X800/MDS7.jpg]

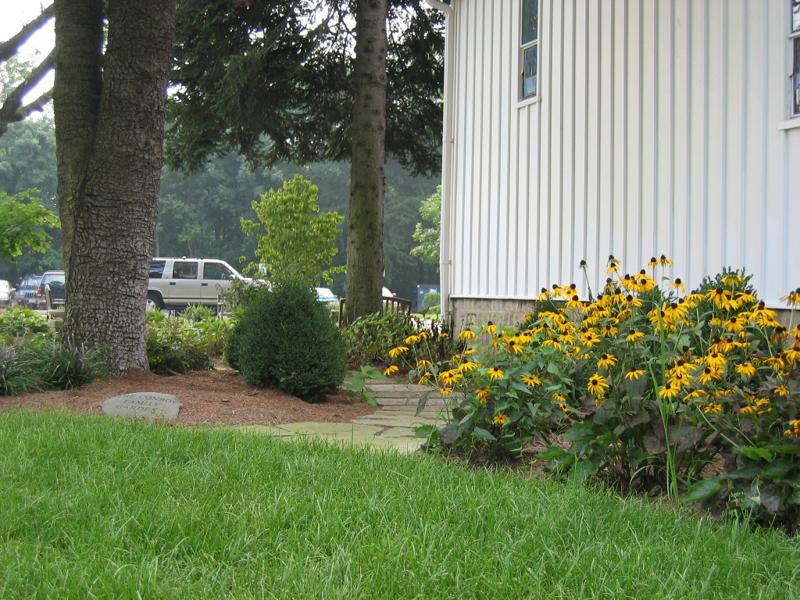

Supplement: S2 Data — Images used in our study. (ZIP) [file pone.0114572.s002.zip › Stimuli/MDS600X800/MDS70.jpg]

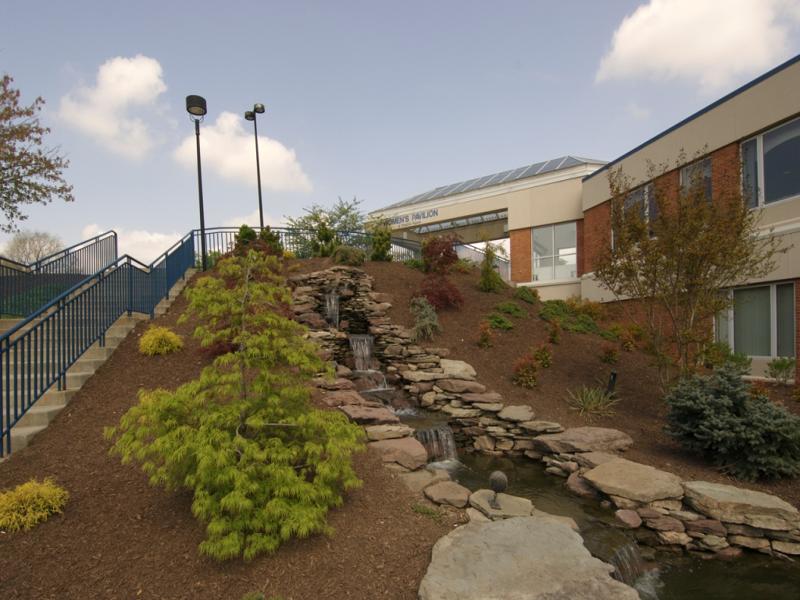

Supplement: S2 Data — Images used in our study. (ZIP) [file pone.0114572.s002.zip › Stimuli/MDS600X800/MDS71.jpg]

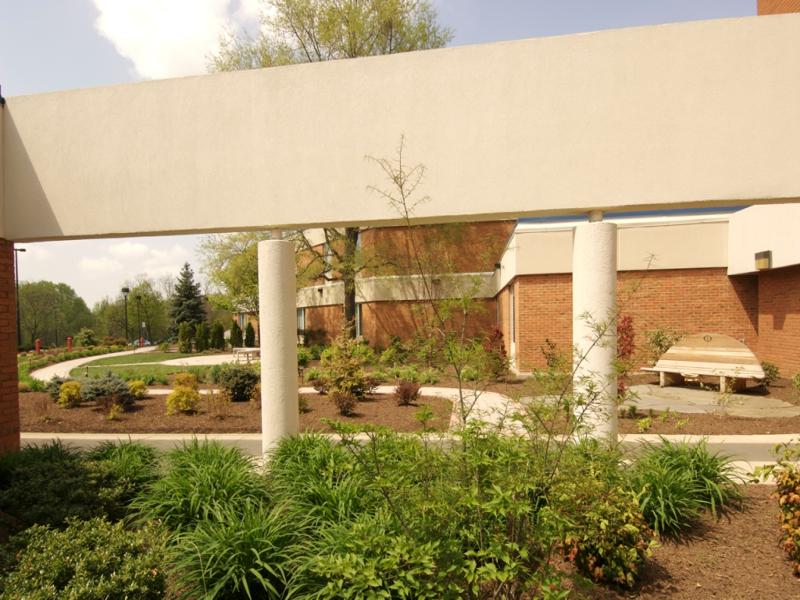

Supplement: S2 Data — Images used in our study. (ZIP) [file pone.0114572.s002.zip › Stimuli/MDS600X800/MDS72.jpg]

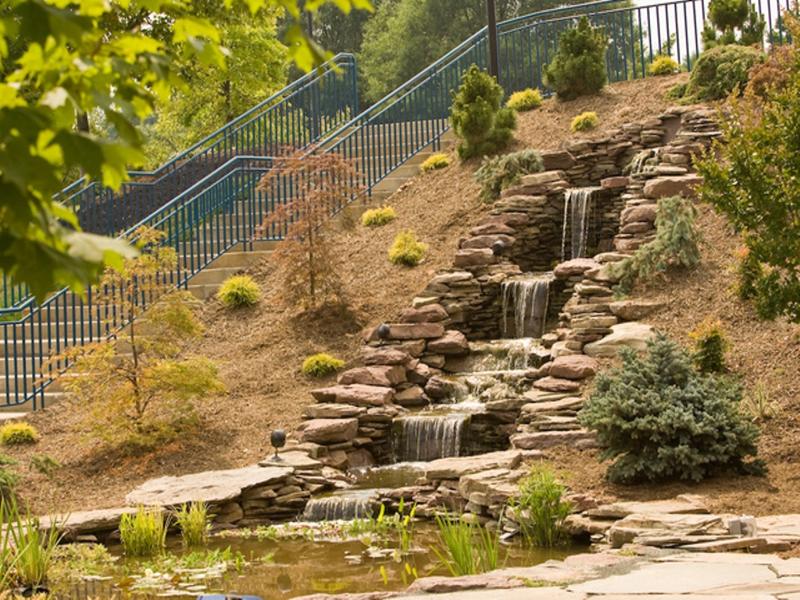

Supplement: S2 Data — Images used in our study. (ZIP) [file pone.0114572.s002.zip › Stimuli/MDS600X800/MDS73.jpg]

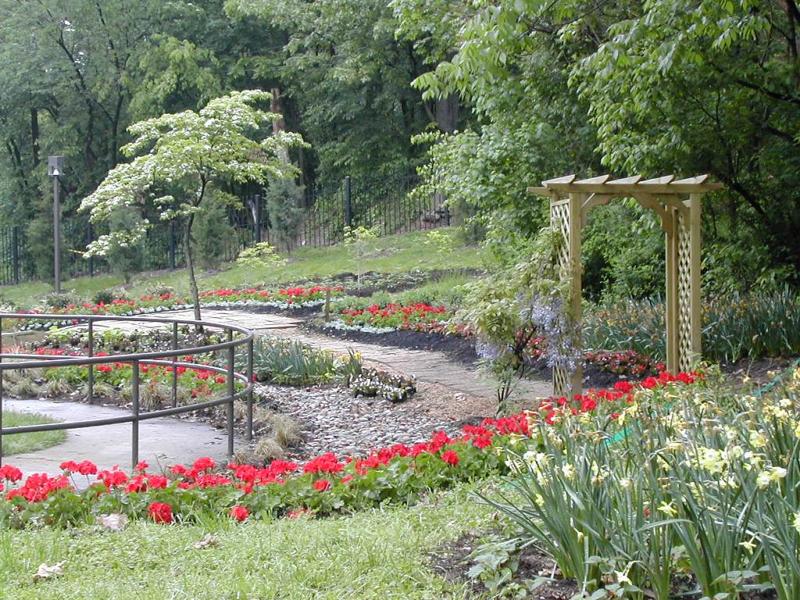

Supplement: S2 Data — Images used in our study. (ZIP) [file pone.0114572.s002.zip › Stimuli/MDS600X800/MDS74.jpg]

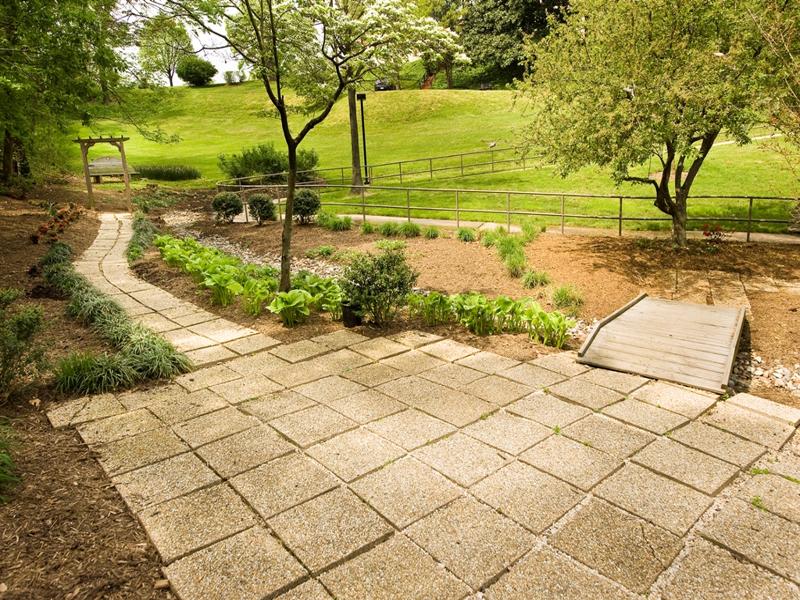

Supplement: S2 Data — Images used in our study. (ZIP) [file pone.0114572.s002.zip › Stimuli/MDS600X800/MDS75.jpg]

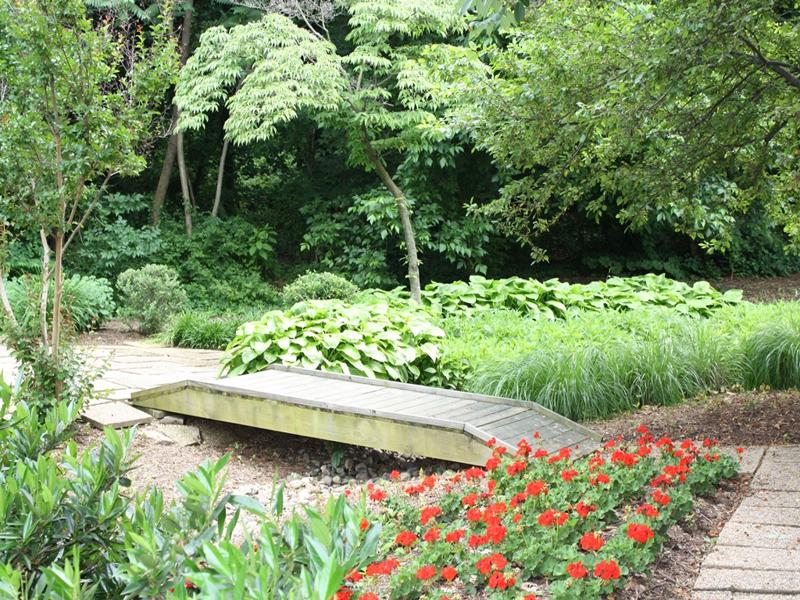

Supplement: S2 Data — Images used in our study. (ZIP) [file pone.0114572.s002.zip › Stimuli/MDS600X800/MDS76.jpg]

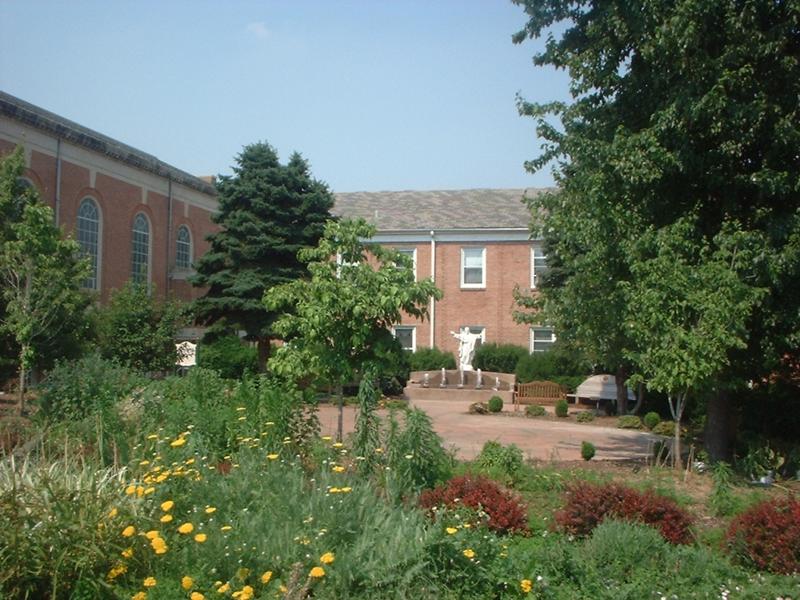

Supplement: S2 Data — Images used in our study. (ZIP) [file pone.0114572.s002.zip › Stimuli/MDS600X800/MDS77.jpg]

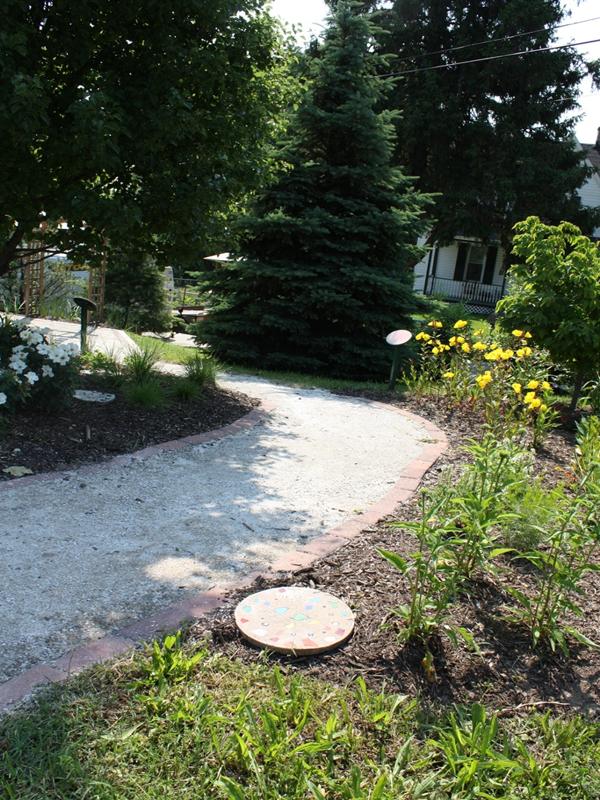

Supplement: S2 Data — Images used in our study. (ZIP) [file pone.0114572.s002.zip › Stimuli/MDS600X800/MDS78.jpg]

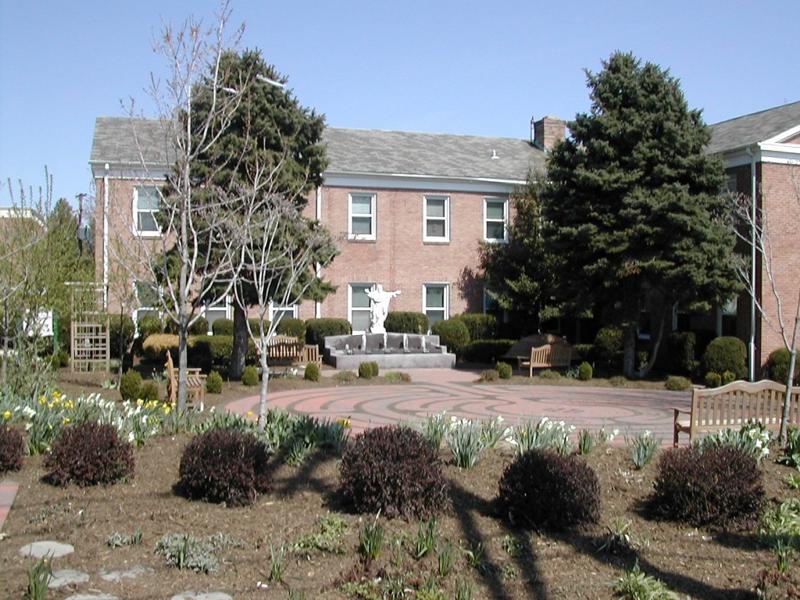

Supplement: S2 Data — Images used in our study. (ZIP) [file pone.0114572.s002.zip › Stimuli/MDS600X800/MDS79.jpg]

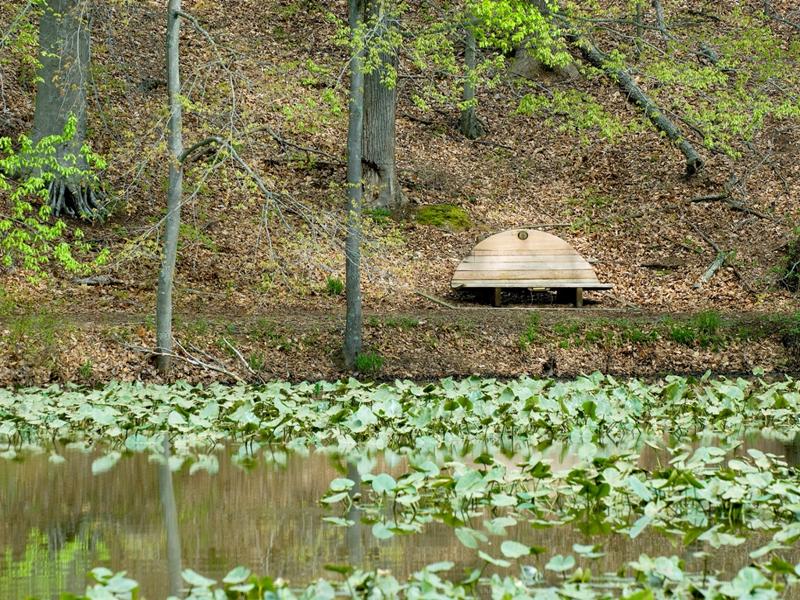

Supplement: S2 Data — Images used in our study. (ZIP) [file pone.0114572.s002.zip › Stimuli/MDS600X800/MDS8.jpg]

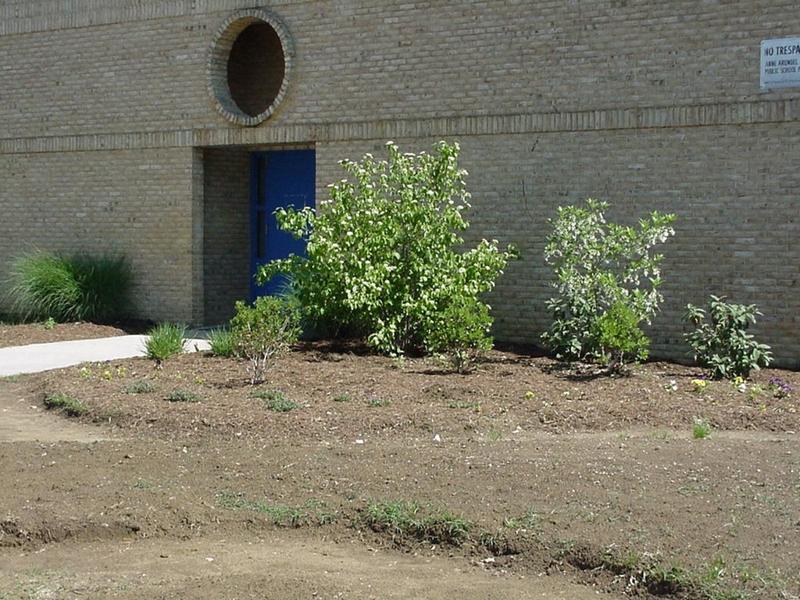

Supplement: S2 Data — Images used in our study. (ZIP) [file pone.0114572.s002.zip › Stimuli/MDS600X800/MDS80.jpg]

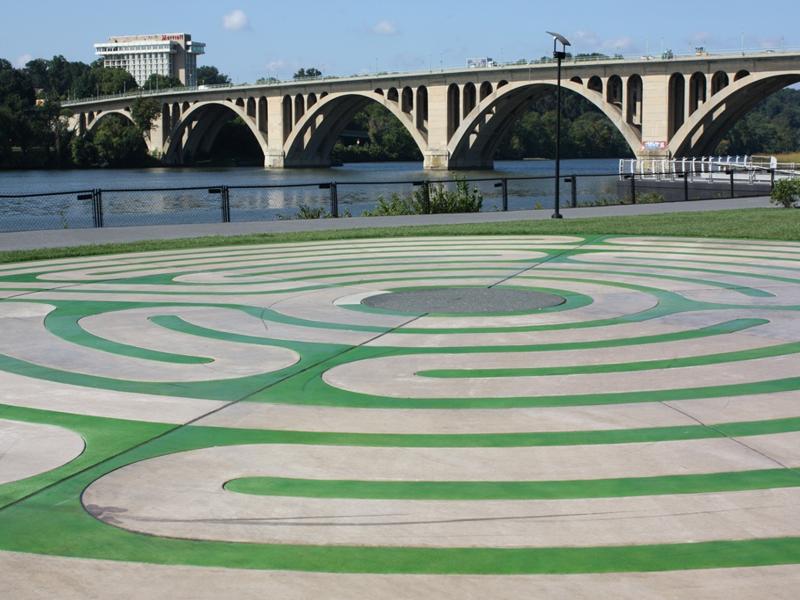

Supplement: S2 Data — Images used in our study. (ZIP) [file pone.0114572.s002.zip › Stimuli/MDS600X800/MDS81.jpg]

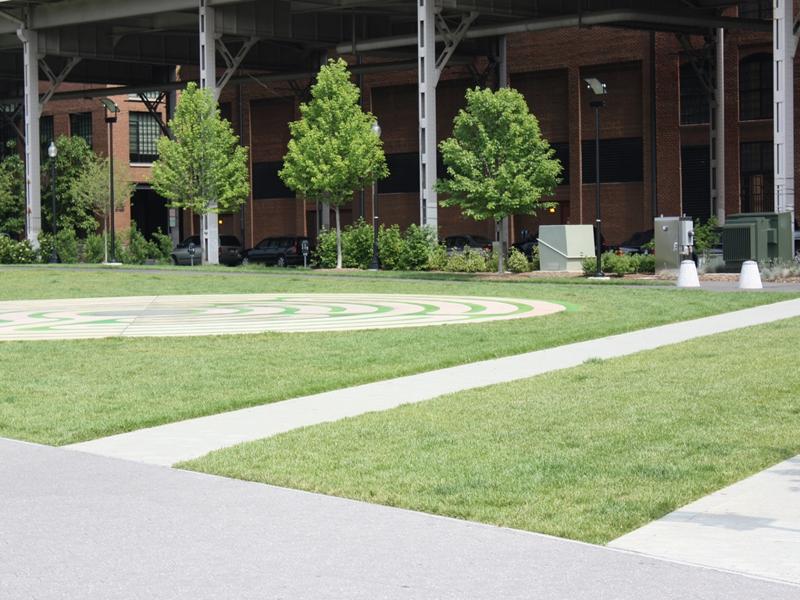

Supplement: S2 Data — Images used in our study. (ZIP) [file pone.0114572.s002.zip › Stimuli/MDS600X800/MDS82.jpg]

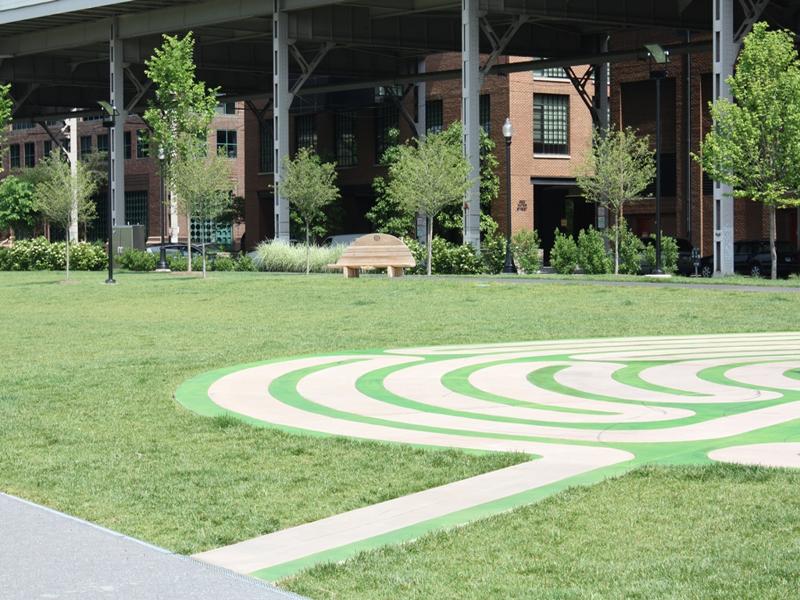

Supplement: S2 Data — Images used in our study. (ZIP) [file pone.0114572.s002.zip › Stimuli/MDS600X800/MDS83.jpg]

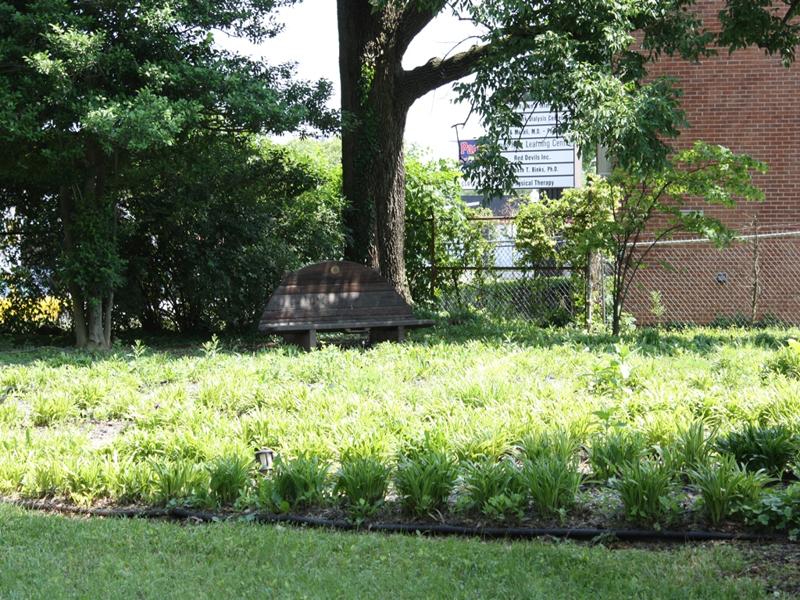

Supplement: S2 Data — Images used in our study. (ZIP) [file pone.0114572.s002.zip › Stimuli/MDS600X800/MDS84.jpg]

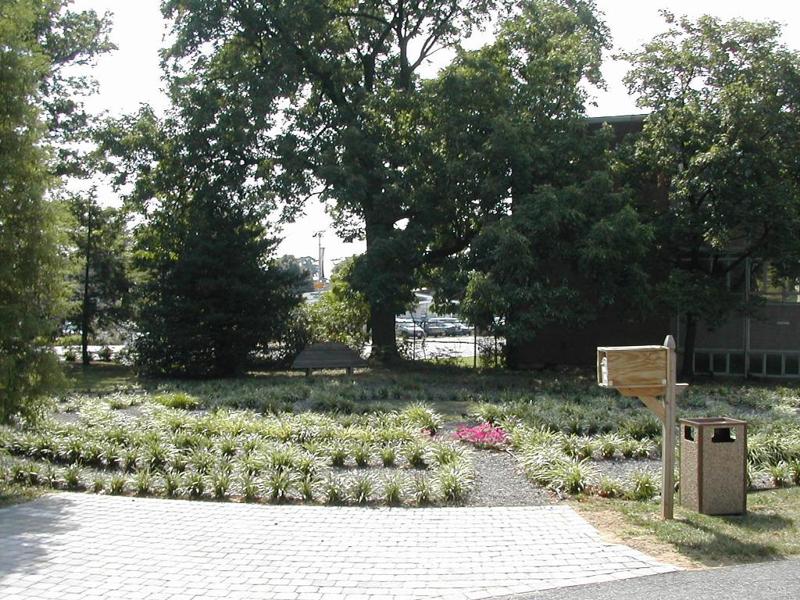

Supplement: S2 Data — Images used in our study. (ZIP) [file pone.0114572.s002.zip › Stimuli/MDS600X800/MDS85.jpg]

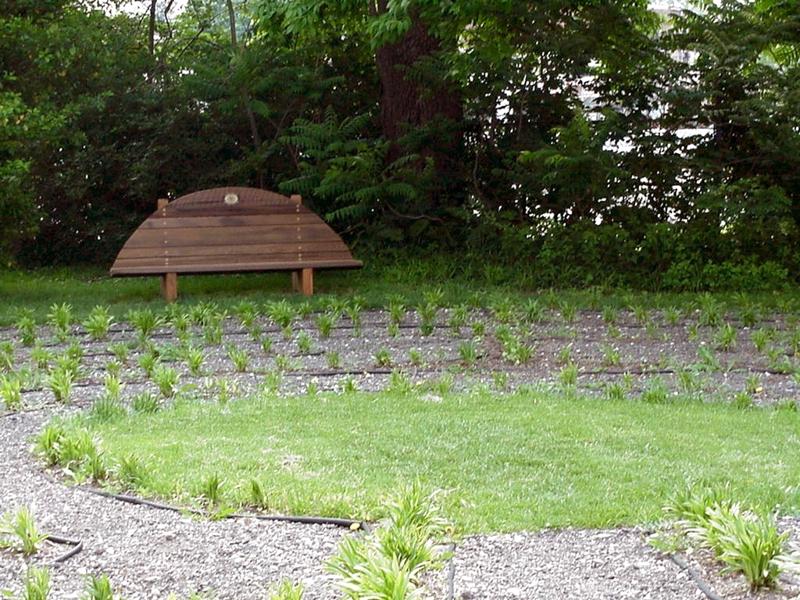

Supplement: S2 Data — Images used in our study. (ZIP) [file pone.0114572.s002.zip › Stimuli/MDS600X800/MDS86.jpg]

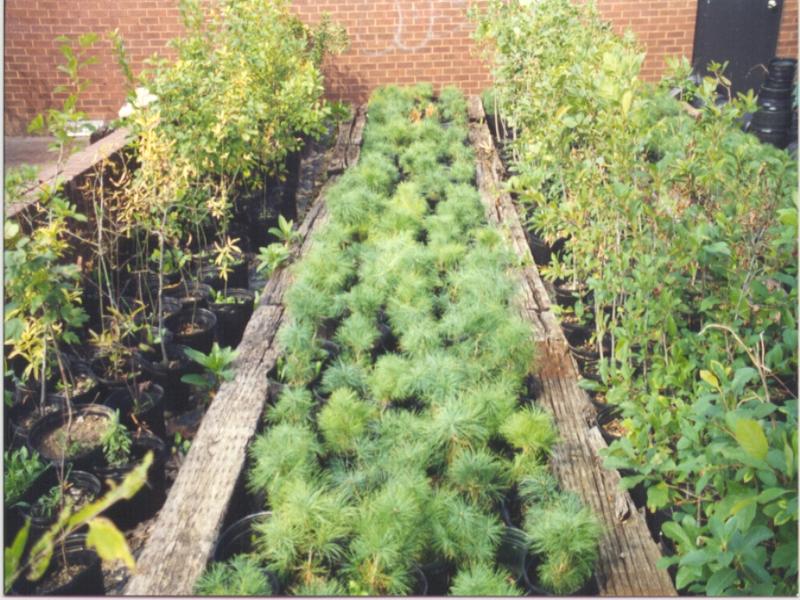

Supplement: S2 Data — Images used in our study. (ZIP) [file pone.0114572.s002.zip › Stimuli/MDS600X800/MDS87.jpg]

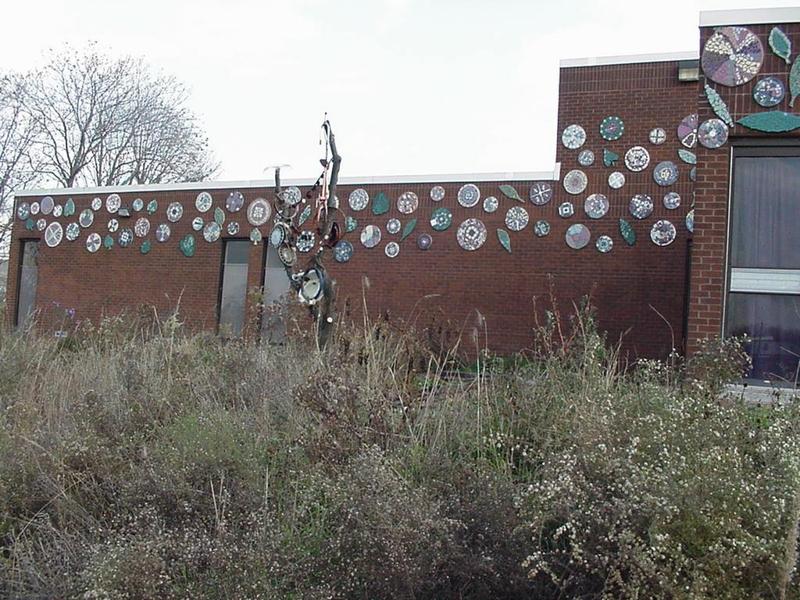

Supplement: S2 Data — Images used in our study. (ZIP) [file pone.0114572.s002.zip › Stimuli/MDS600X800/MDS88.jpg]

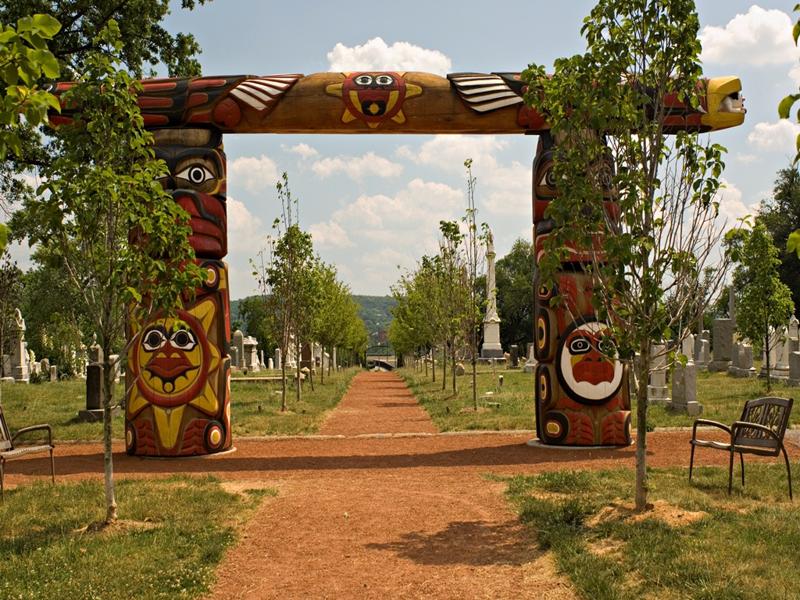

Supplement: S2 Data — Images used in our study. (ZIP) [file pone.0114572.s002.zip › Stimuli/MDS600X800/MDS89.jpg]

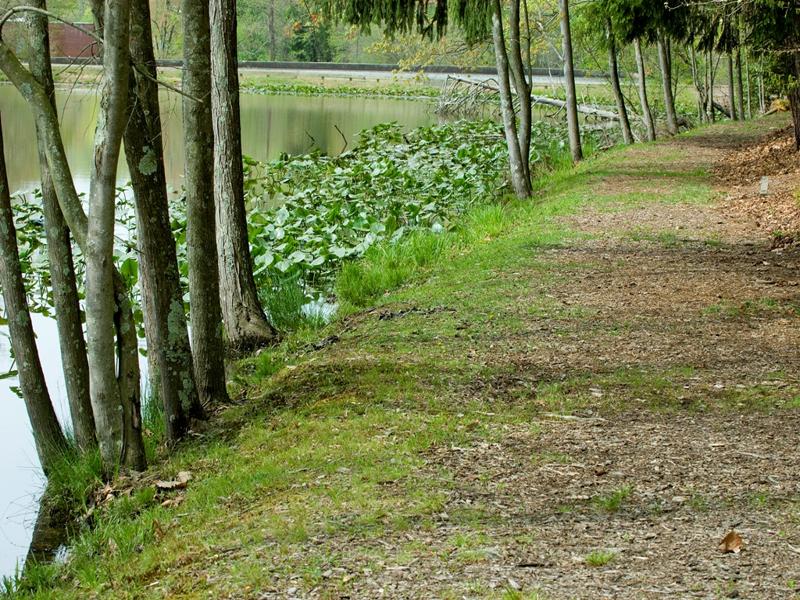

Supplement: S2 Data — Images used in our study. (ZIP) [file pone.0114572.s002.zip › Stimuli/MDS600X800/MDS9.jpg]

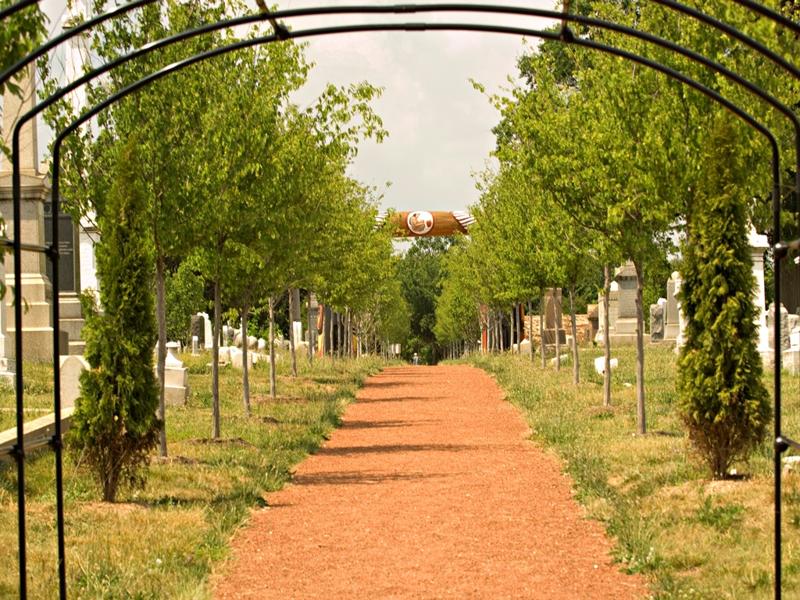

Supplement: S2 Data — Images used in our study. (ZIP) [file pone.0114572.s002.zip › Stimuli/MDS600X800/MDS90.jpg]

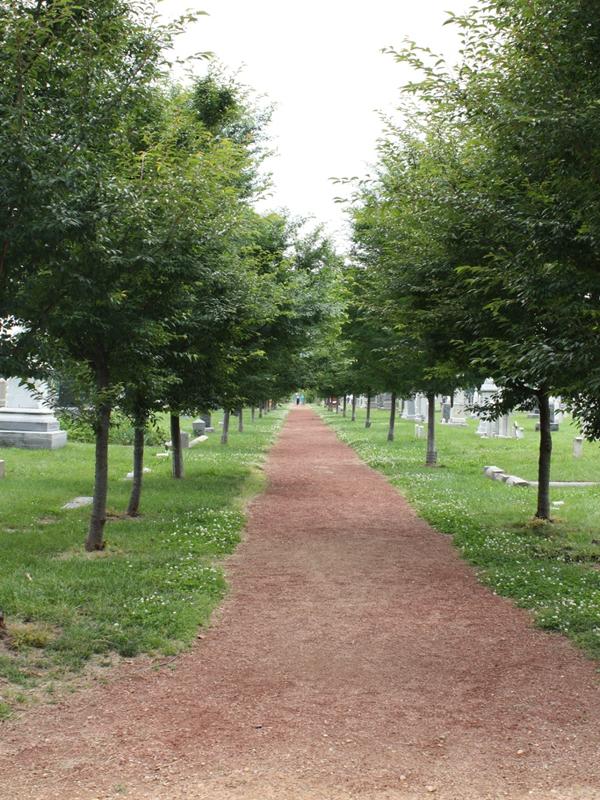

Supplement: S2 Data — Images used in our study. (ZIP) [file pone.0114572.s002.zip › Stimuli/MDS600X800/MDS91.jpg]

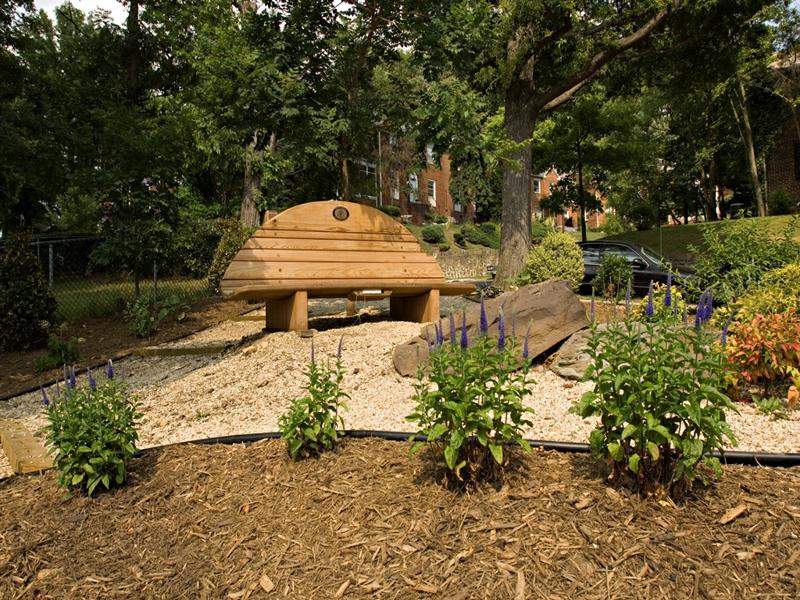

Supplement: S2 Data — Images used in our study. (ZIP) [file pone.0114572.s002.zip › Stimuli/MDS600X800/MDS92.jpg]
