# Supplementary figures and images for: The Perception of Naturalness Correlates with Low-Level Visual Features of Environmental Scenes (part 1 of 2)
Source: PLoS One. 2014 Dec 22;9(12):e114572. doi: 10.1371/journal.pone.0114572 (PMC4273965; doi:10.1371/journal.pone.0114572)

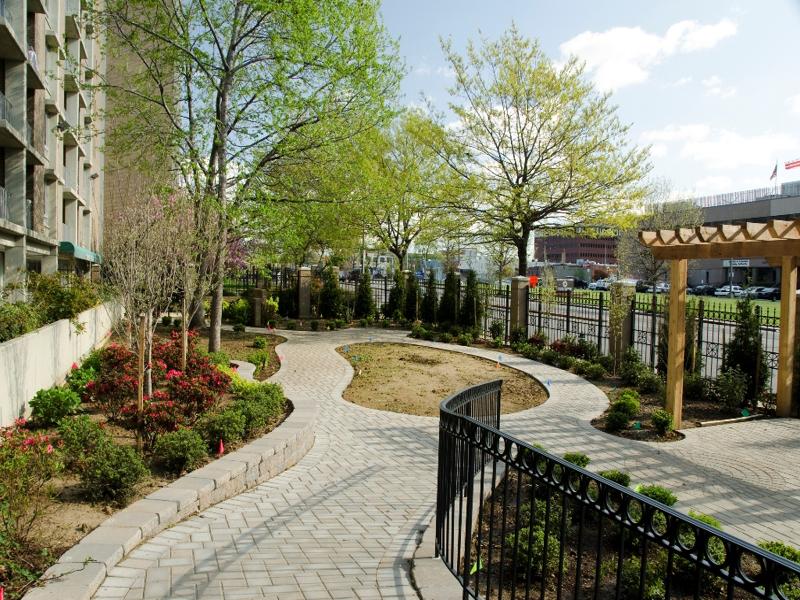

Supplement: S2 Data — Images used in our study. (ZIP) [file pone.0114572.s002.zip › Stimuli/MDS600X800/MDS1.jpg]

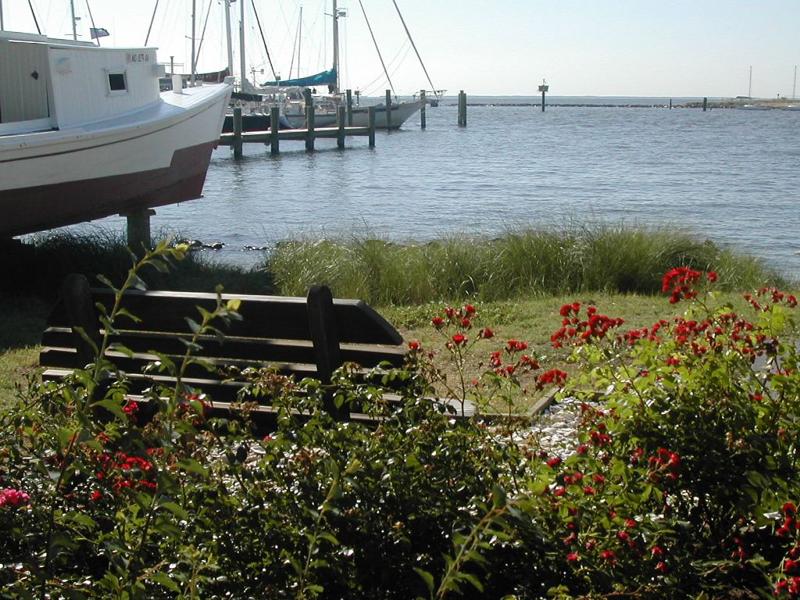

Supplement: S2 Data — Images used in our study. (ZIP) [file pone.0114572.s002.zip › Stimuli/MDS600X800/MDS10.jpg]

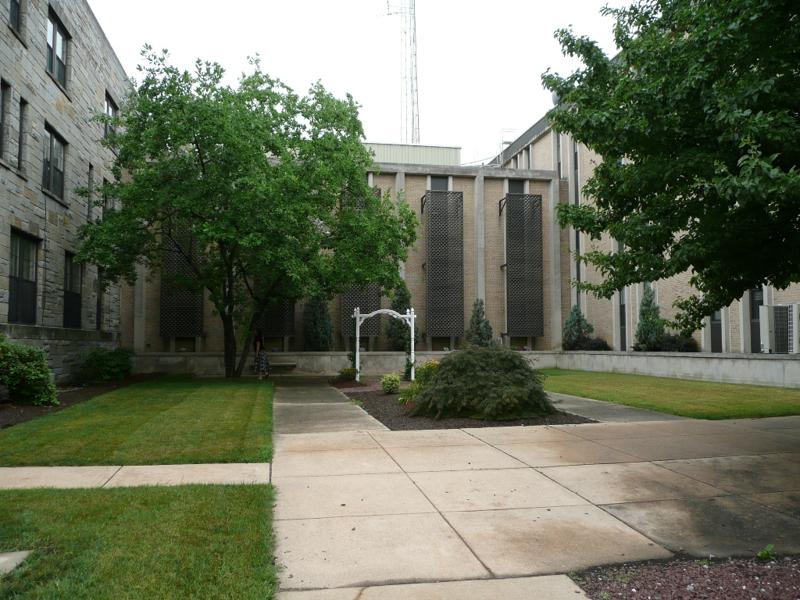

Supplement: S2 Data — Images used in our study. (ZIP) [file pone.0114572.s002.zip › Stimuli/MDS600X800/MDS100.jpg]

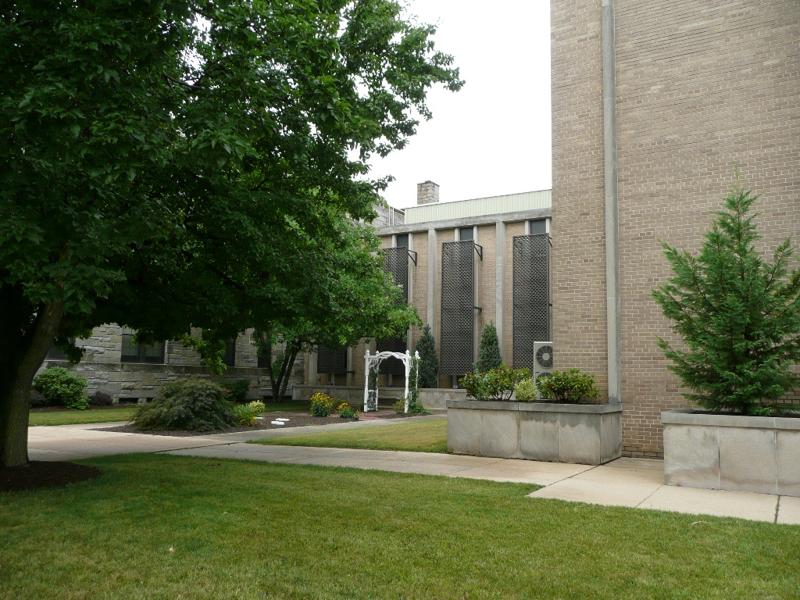

Supplement: S2 Data — Images used in our study. (ZIP) [file pone.0114572.s002.zip › Stimuli/MDS600X800/MDS101.jpg]

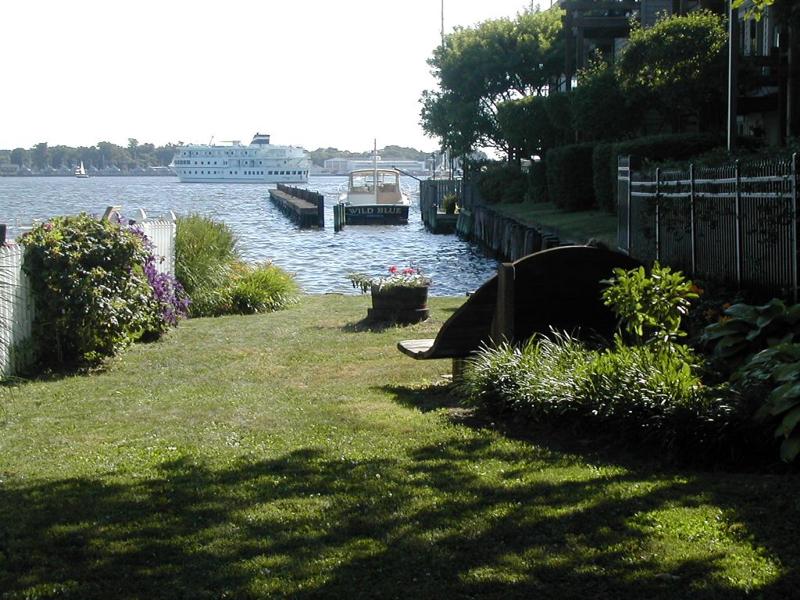

Supplement: S2 Data — Images used in our study. (ZIP) [file pone.0114572.s002.zip › Stimuli/MDS600X800/MDS102.jpg]

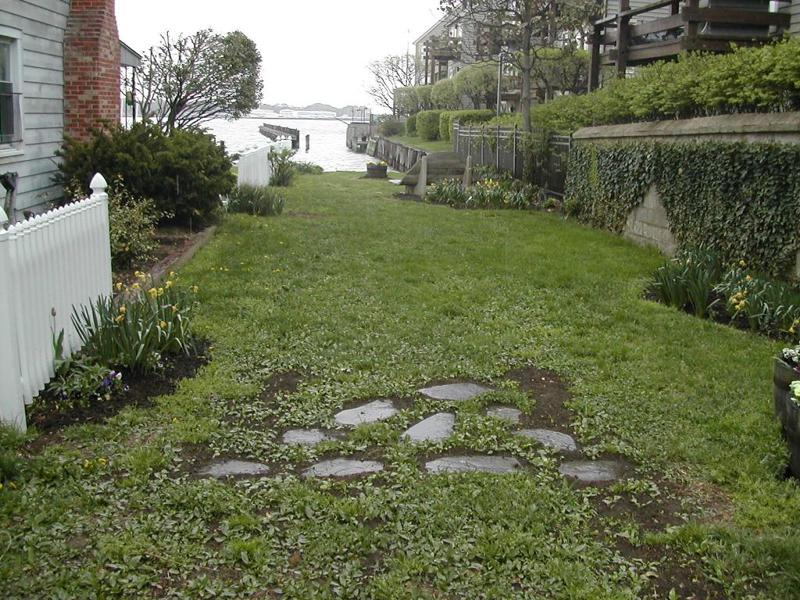

Supplement: S2 Data — Images used in our study. (ZIP) [file pone.0114572.s002.zip › Stimuli/MDS600X800/MDS103.jpg]

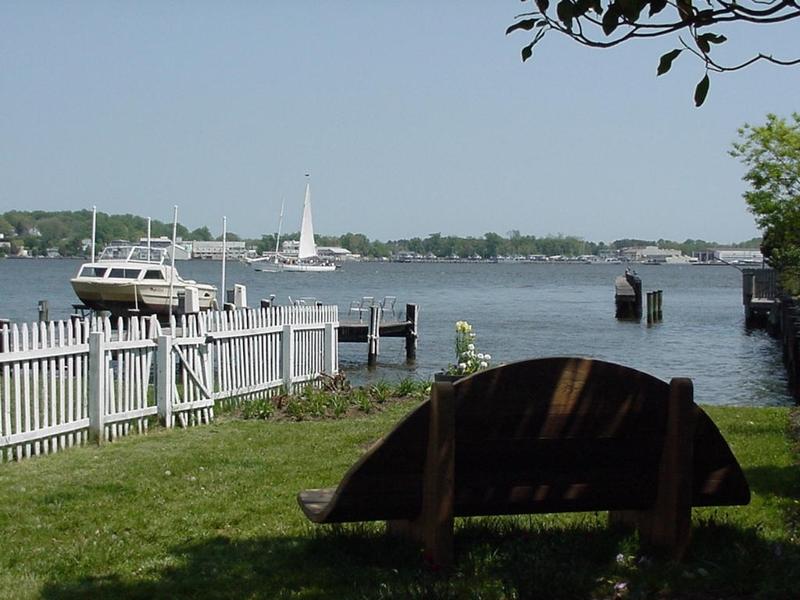

Supplement: S2 Data — Images used in our study. (ZIP) [file pone.0114572.s002.zip › Stimuli/MDS600X800/MDS104.jpg]

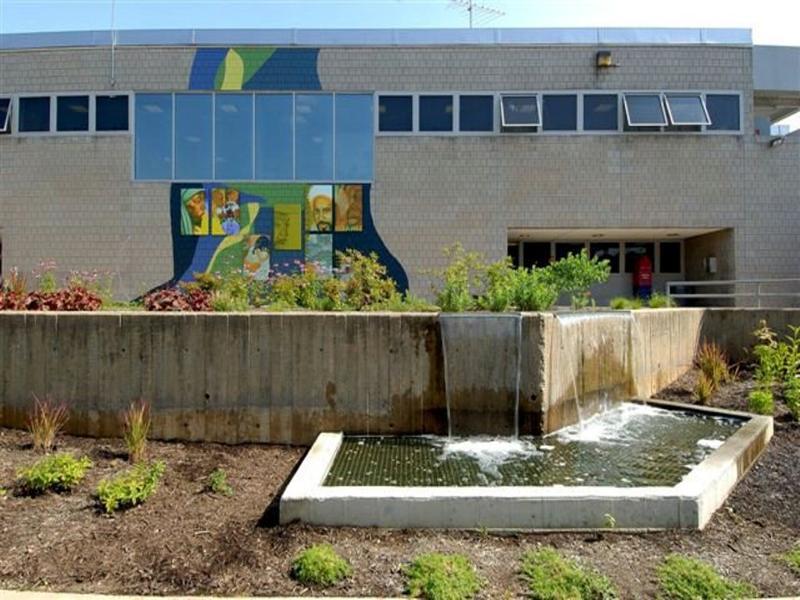

Supplement: S2 Data — Images used in our study. (ZIP) [file pone.0114572.s002.zip › Stimuli/MDS600X800/MDS105.jpg]

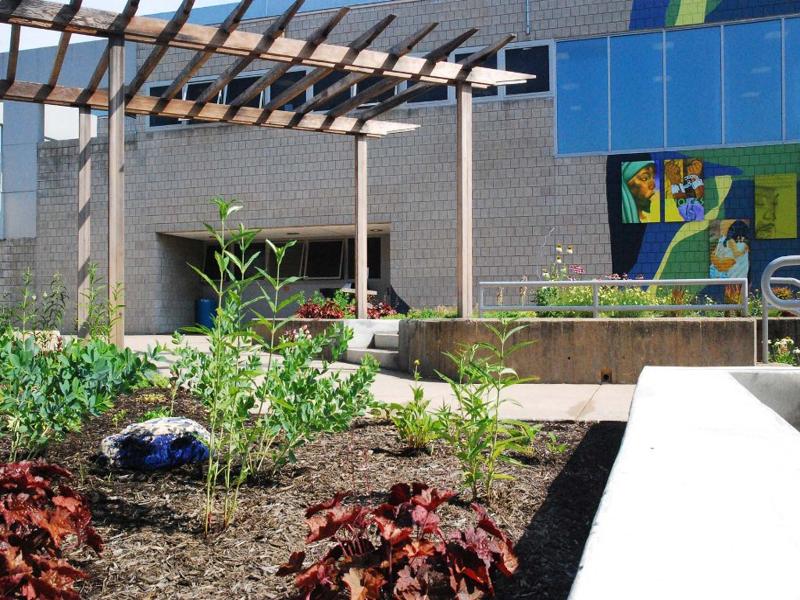

Supplement: S2 Data — Images used in our study. (ZIP) [file pone.0114572.s002.zip › Stimuli/MDS600X800/MDS106.jpg]

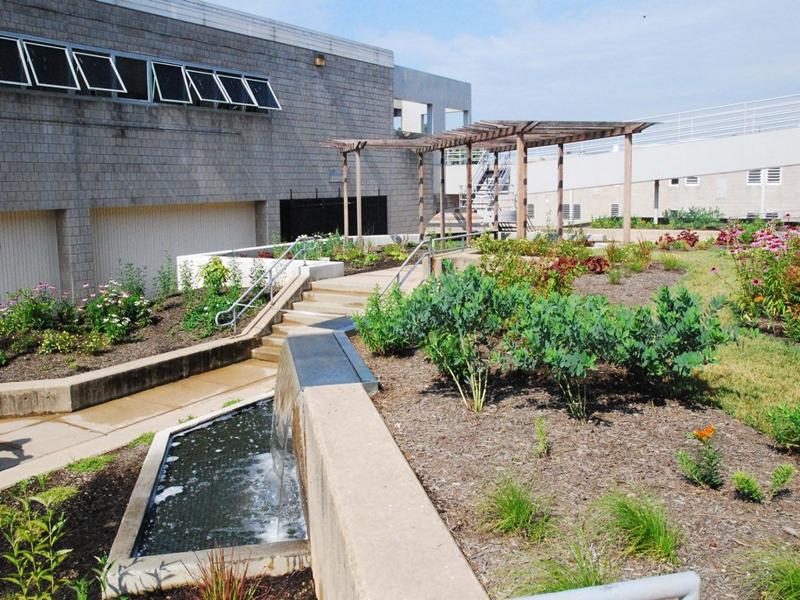

Supplement: S2 Data — Images used in our study. (ZIP) [file pone.0114572.s002.zip › Stimuli/MDS600X800/MDS107.jpg]

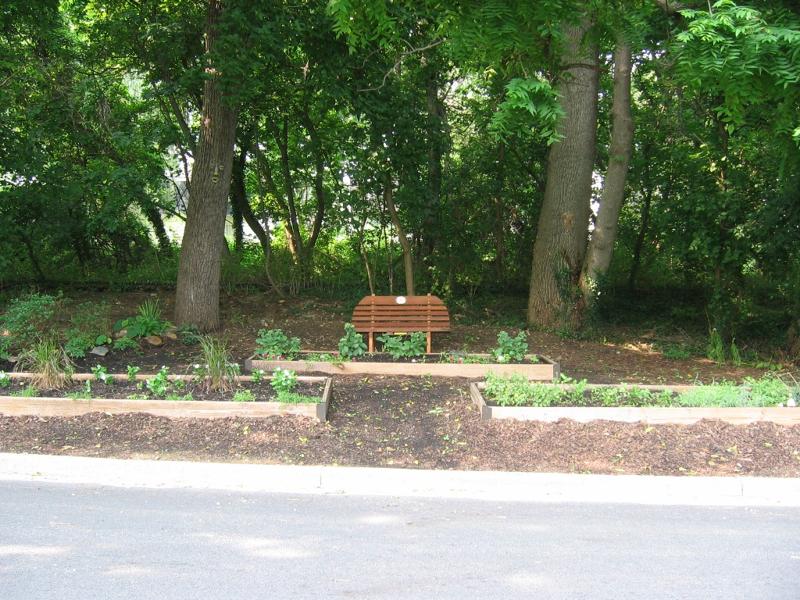

Supplement: S2 Data — Images used in our study. (ZIP) [file pone.0114572.s002.zip › Stimuli/MDS600X800/MDS108.jpg]

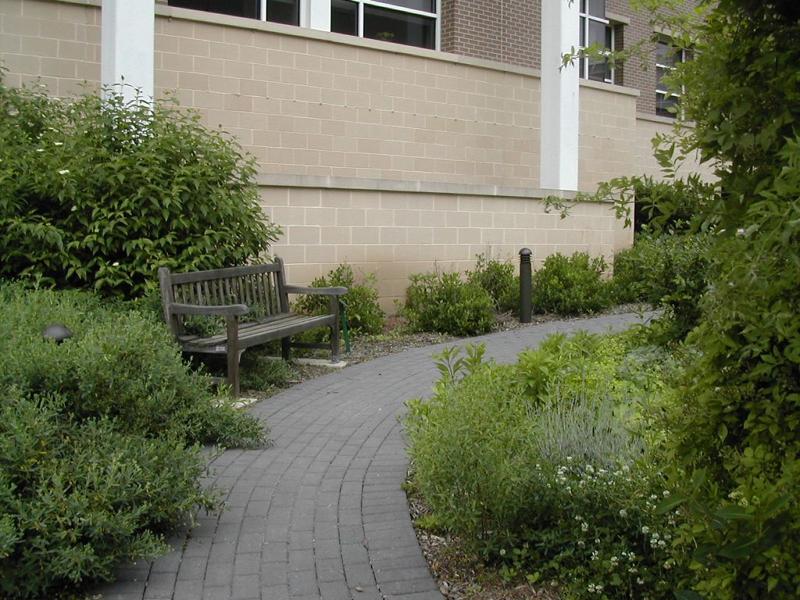

Supplement: S2 Data — Images used in our study. (ZIP) [file pone.0114572.s002.zip › Stimuli/MDS600X800/MDS109.jpg]

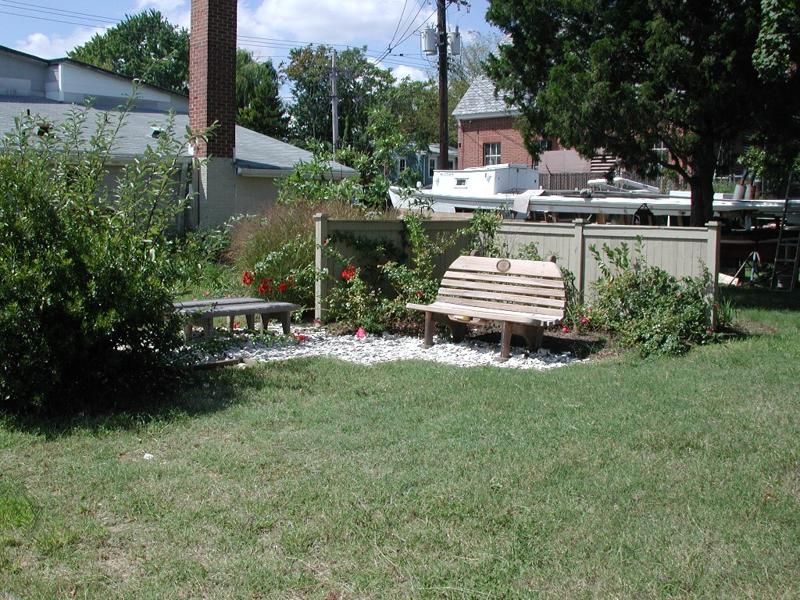

Supplement: S2 Data — Images used in our study. (ZIP) [file pone.0114572.s002.zip › Stimuli/MDS600X800/MDS11.jpg]

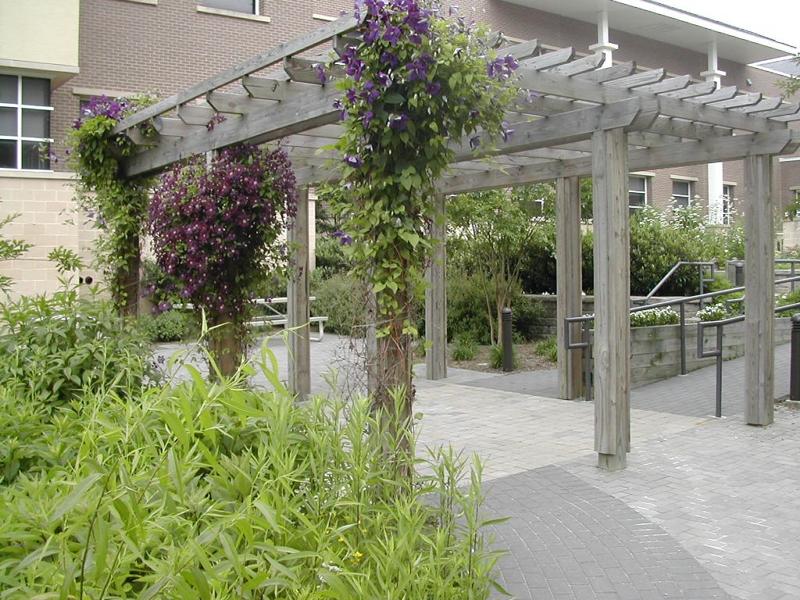

Supplement: S2 Data — Images used in our study. (ZIP) [file pone.0114572.s002.zip › Stimuli/MDS600X800/MDS110.jpg]

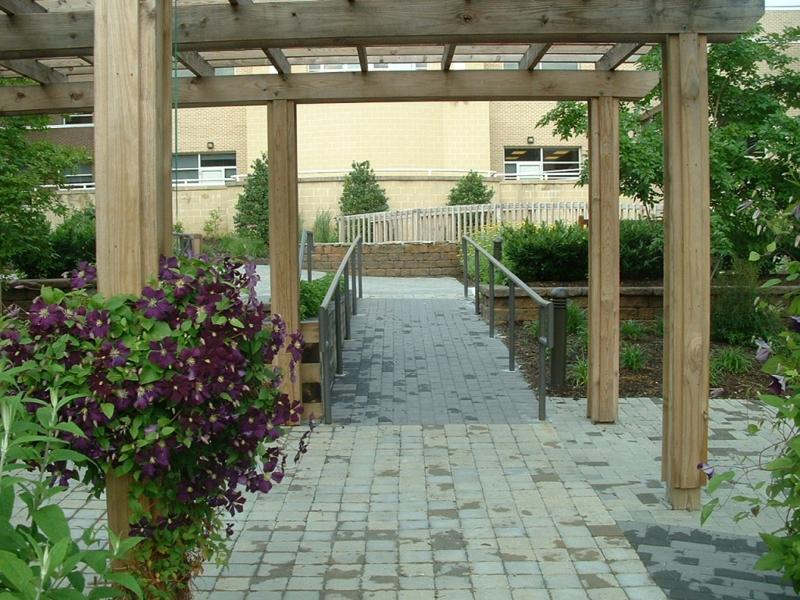

Supplement: S2 Data — Images used in our study. (ZIP) [file pone.0114572.s002.zip › Stimuli/MDS600X800/MDS111.jpg]

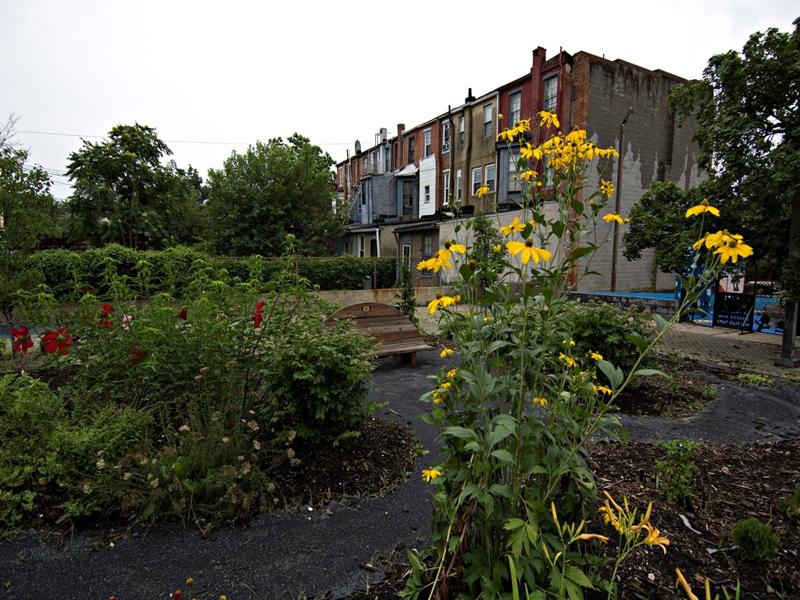

Supplement: S2 Data — Images used in our study. (ZIP) [file pone.0114572.s002.zip › Stimuli/MDS600X800/MDS112.jpg]

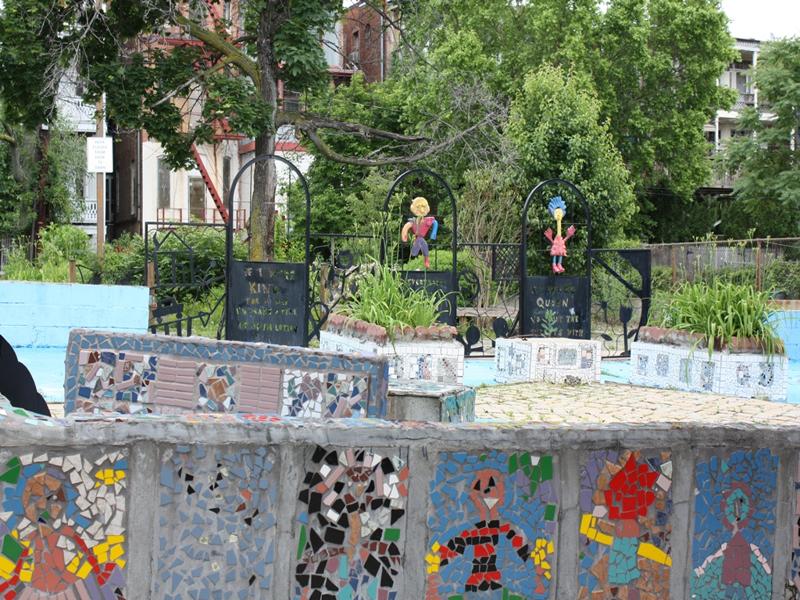

Supplement: S2 Data — Images used in our study. (ZIP) [file pone.0114572.s002.zip › Stimuli/MDS600X800/MDS113.jpg]

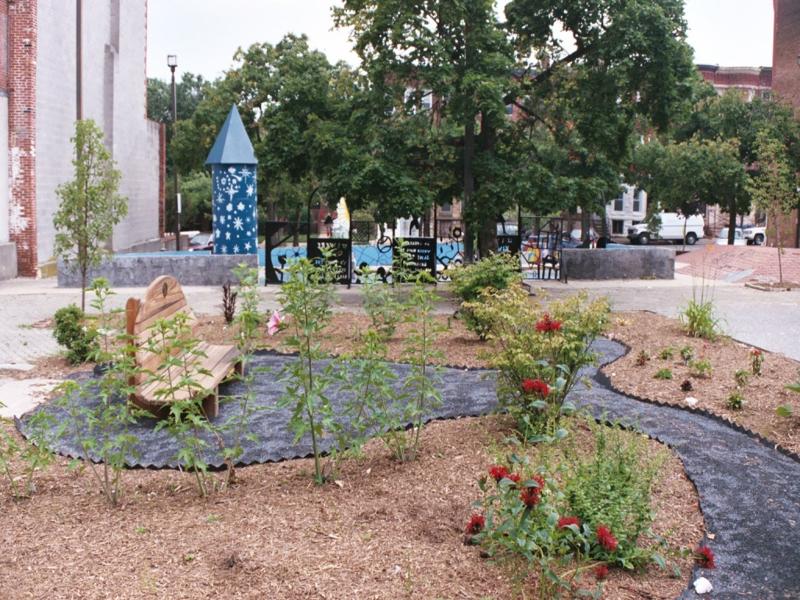

Supplement: S2 Data — Images used in our study. (ZIP) [file pone.0114572.s002.zip › Stimuli/MDS600X800/MDS114.jpg]

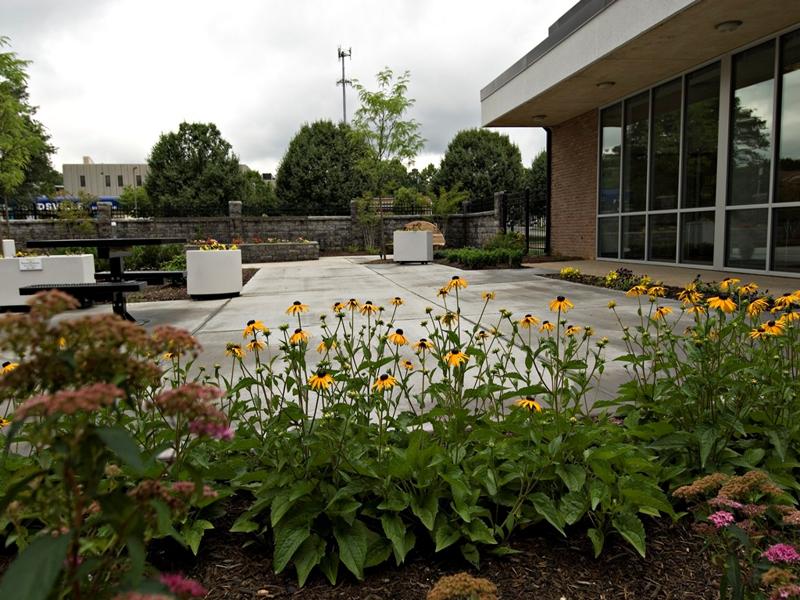

Supplement: S2 Data — Images used in our study. (ZIP) [file pone.0114572.s002.zip › Stimuli/MDS600X800/MDS115.jpg]

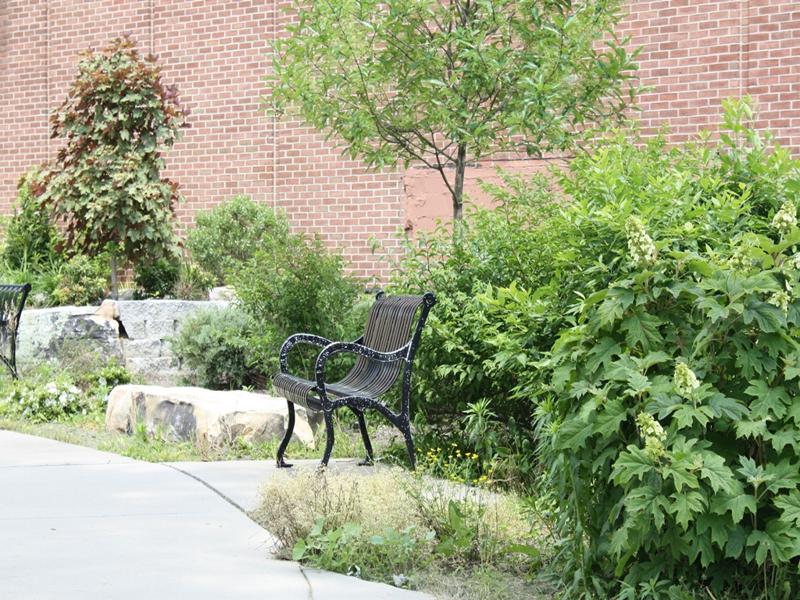

Supplement: S2 Data — Images used in our study. (ZIP) [file pone.0114572.s002.zip › Stimuli/MDS600X800/MDS116.jpg]

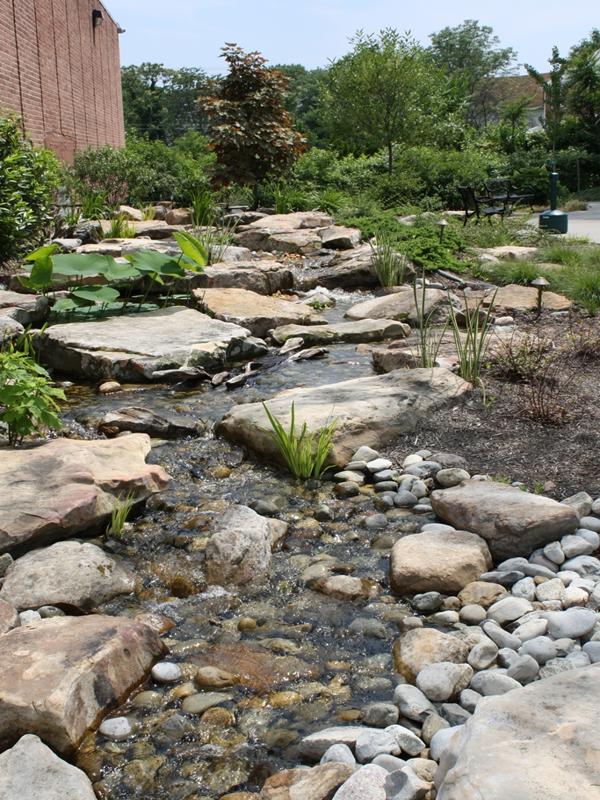

Supplement: S2 Data — Images used in our study. (ZIP) [file pone.0114572.s002.zip › Stimuli/MDS600X800/MDS117.jpg]

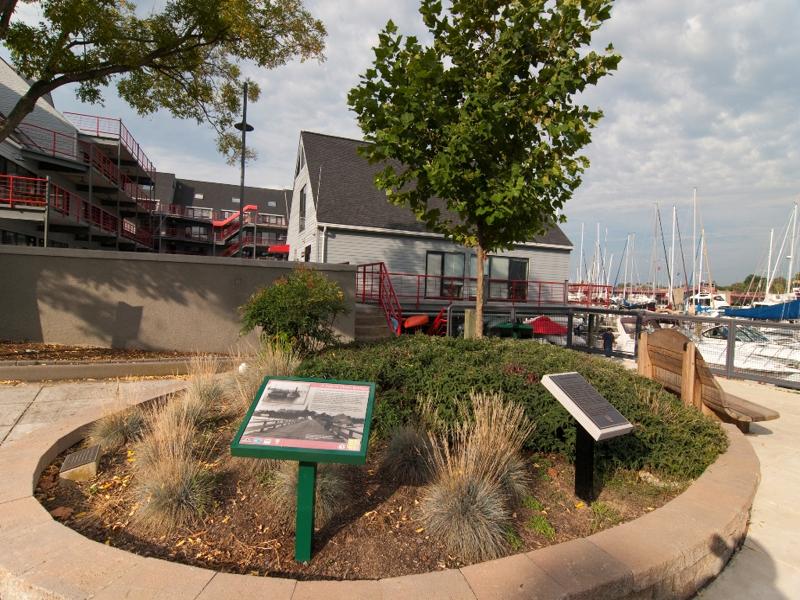

Supplement: S2 Data — Images used in our study. (ZIP) [file pone.0114572.s002.zip › Stimuli/MDS600X800/MDS118.jpg]

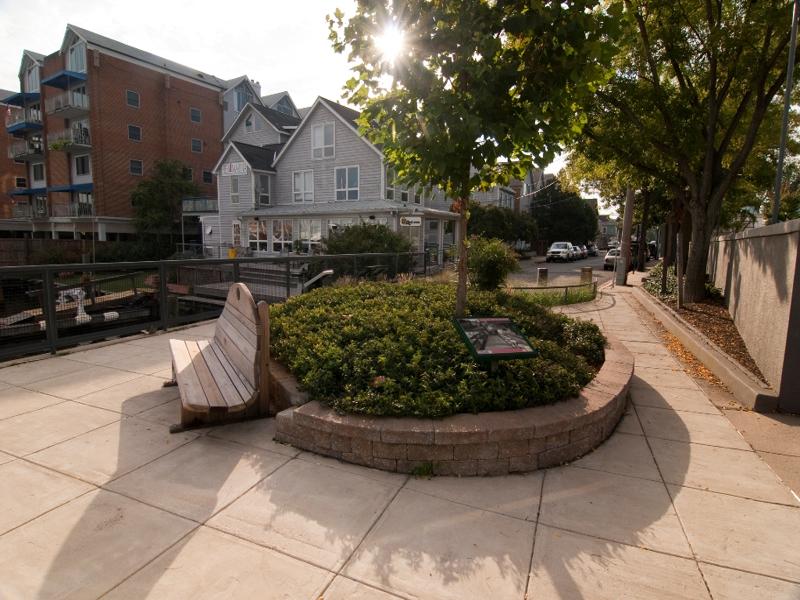

Supplement: S2 Data — Images used in our study. (ZIP) [file pone.0114572.s002.zip › Stimuli/MDS600X800/MDS119.jpg]

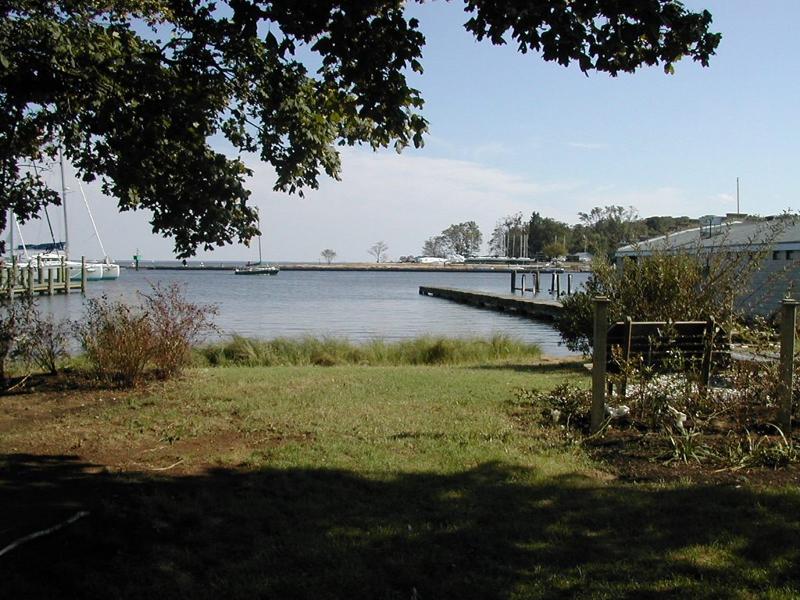

Supplement: S2 Data — Images used in our study. (ZIP) [file pone.0114572.s002.zip › Stimuli/MDS600X800/MDS12.jpg]

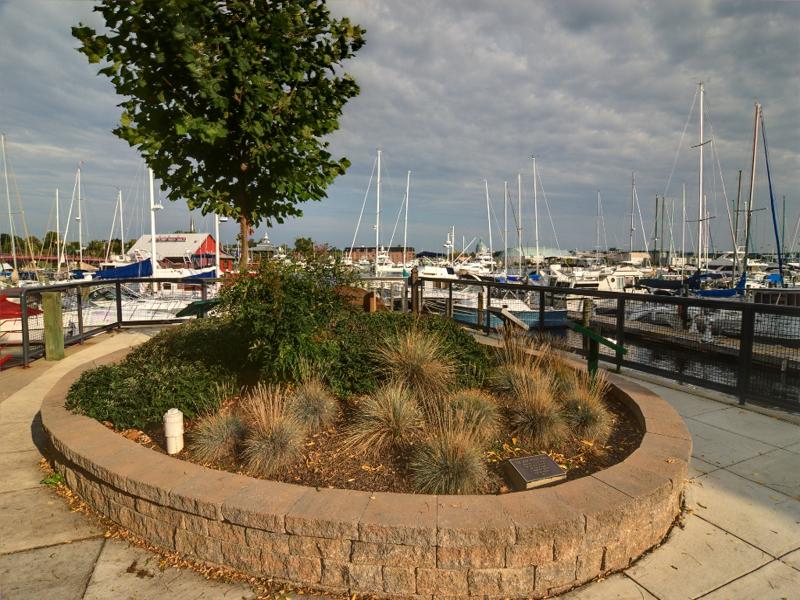

Supplement: S2 Data — Images used in our study. (ZIP) [file pone.0114572.s002.zip › Stimuli/MDS600X800/MDS120.jpg]

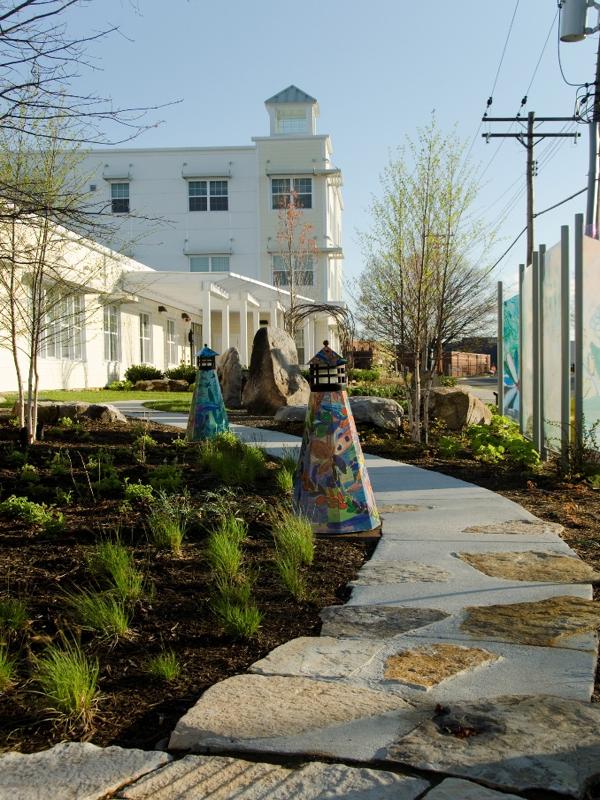

Supplement: S2 Data — Images used in our study. (ZIP) [file pone.0114572.s002.zip › Stimuli/MDS600X800/MDS121.jpg]

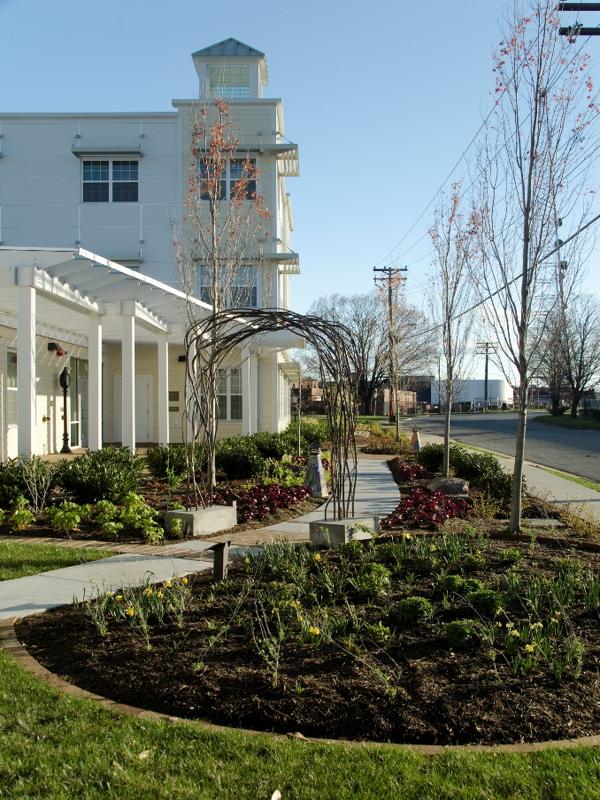

Supplement: S2 Data — Images used in our study. (ZIP) [file pone.0114572.s002.zip › Stimuli/MDS600X800/MDS122.jpg]

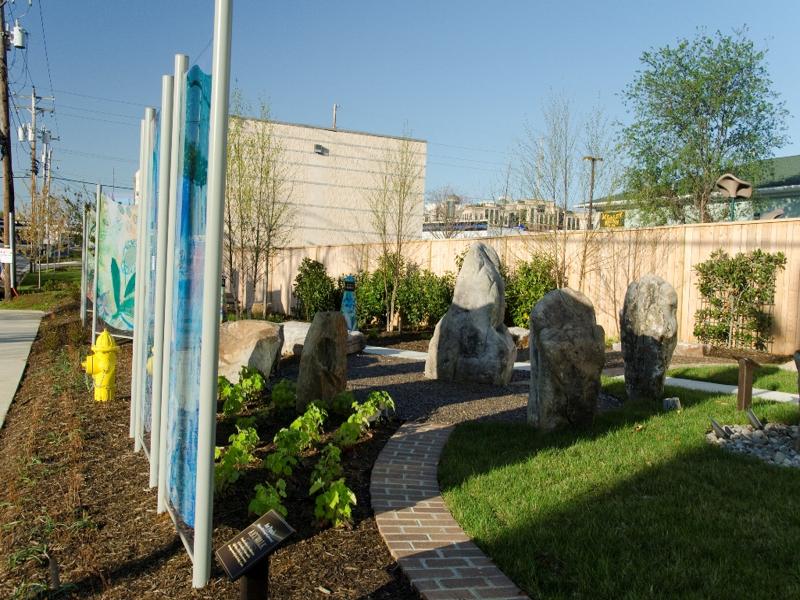

Supplement: S2 Data — Images used in our study. (ZIP) [file pone.0114572.s002.zip › Stimuli/MDS600X800/MDS123.jpg]

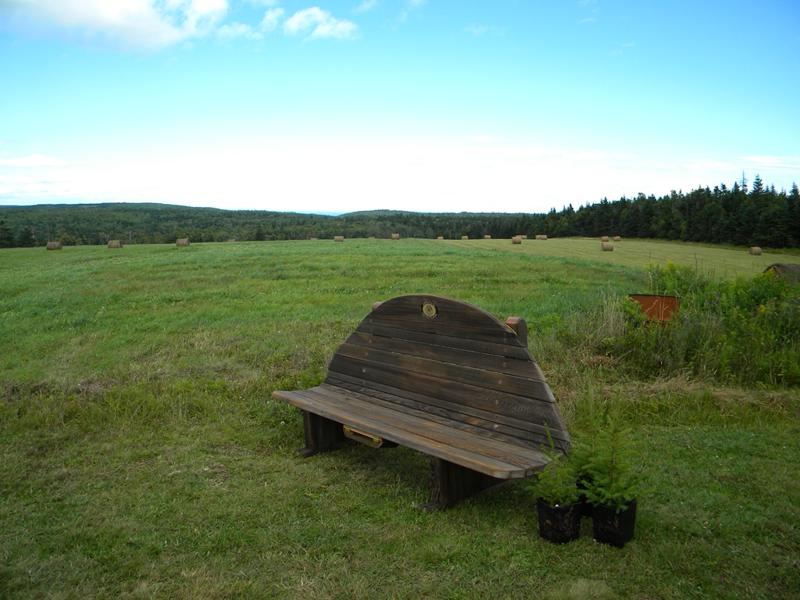

Supplement: S2 Data — Images used in our study. (ZIP) [file pone.0114572.s002.zip › Stimuli/MDS600X800/MDS124.jpg]

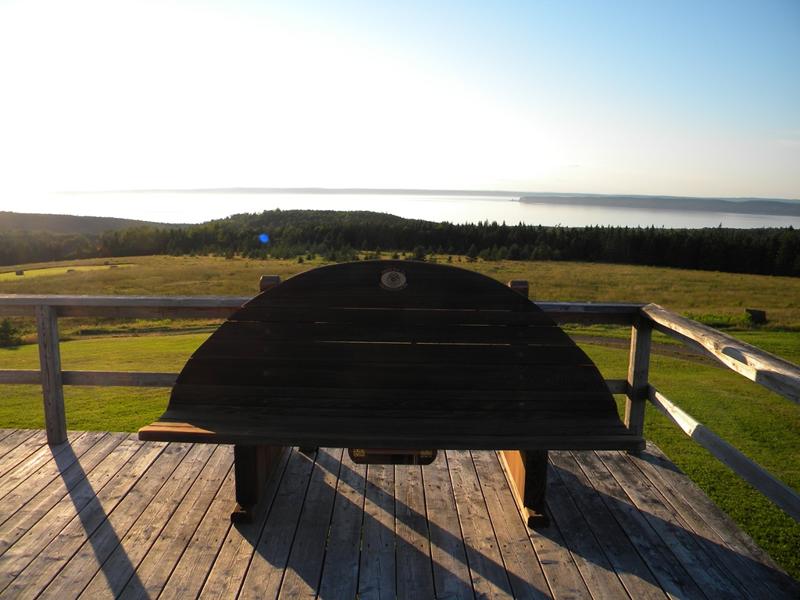

Supplement: S2 Data — Images used in our study. (ZIP) [file pone.0114572.s002.zip › Stimuli/MDS600X800/MDS125.jpg]

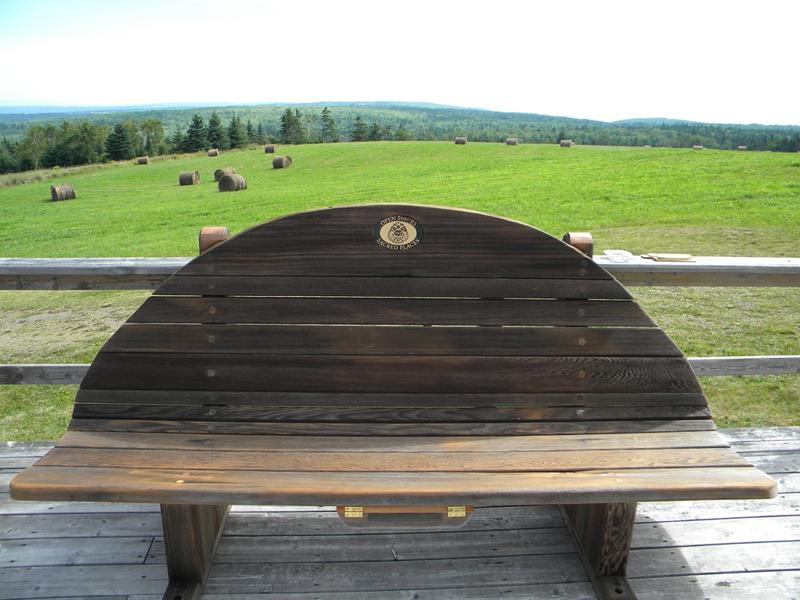

Supplement: S2 Data — Images used in our study. (ZIP) [file pone.0114572.s002.zip › Stimuli/MDS600X800/MDS126.jpg]

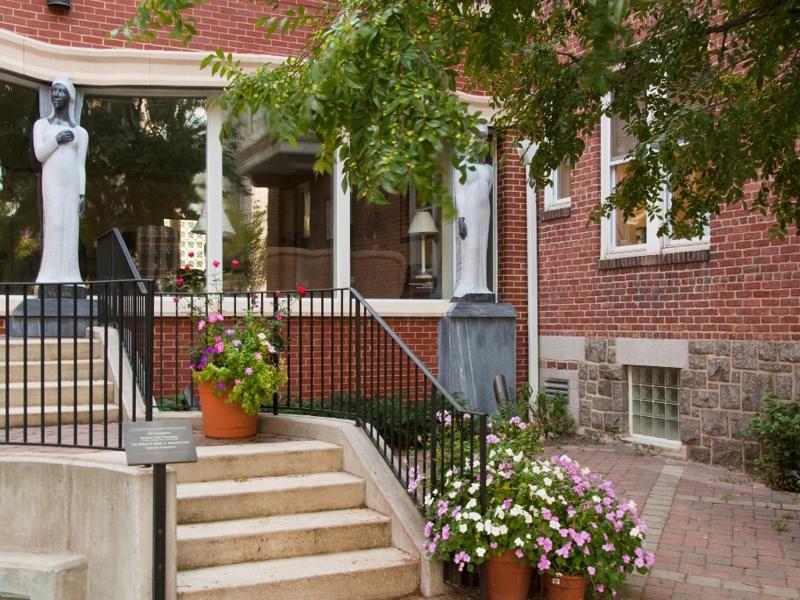

Supplement: S2 Data — Images used in our study. (ZIP) [file pone.0114572.s002.zip › Stimuli/MDS600X800/MDS127.jpg]

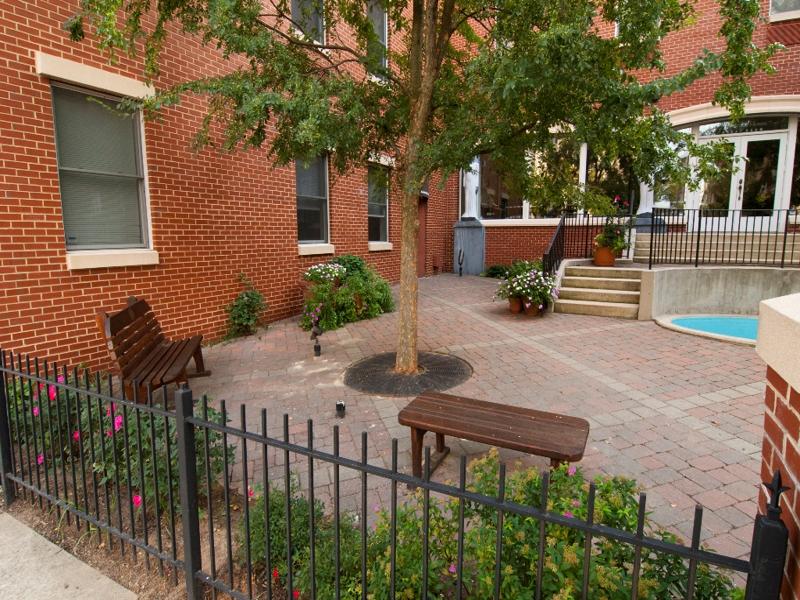

Supplement: S2 Data — Images used in our study. (ZIP) [file pone.0114572.s002.zip › Stimuli/MDS600X800/MDS128.jpg]

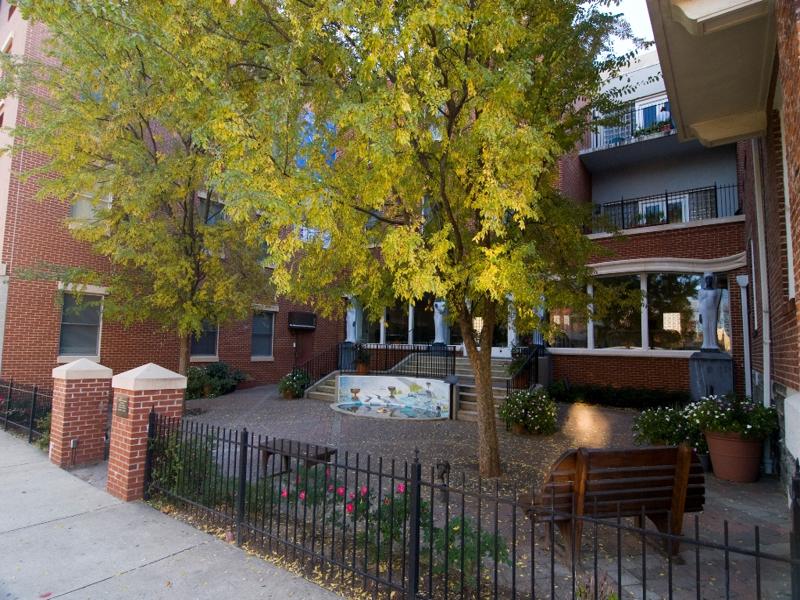

Supplement: S2 Data — Images used in our study. (ZIP) [file pone.0114572.s002.zip › Stimuli/MDS600X800/MDS129.jpg]

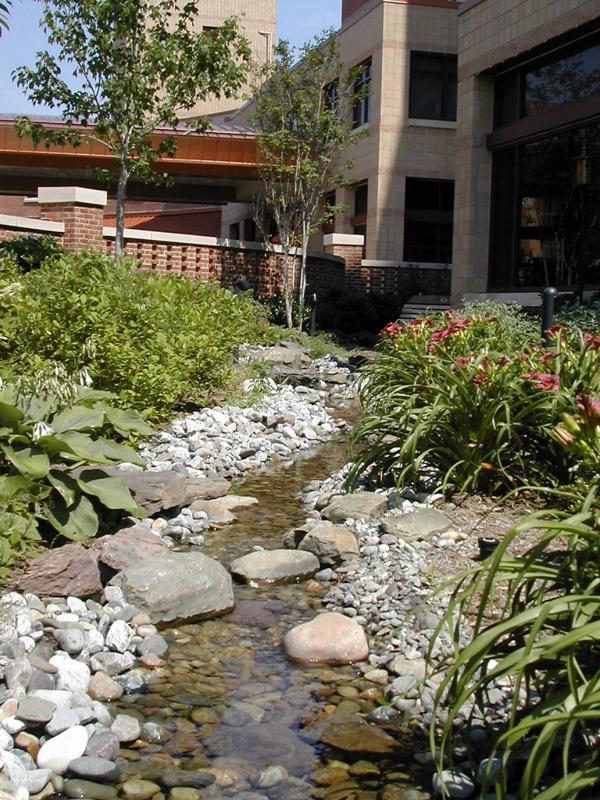

Supplement: S2 Data — Images used in our study. (ZIP) [file pone.0114572.s002.zip › Stimuli/MDS600X800/MDS13.jpg]

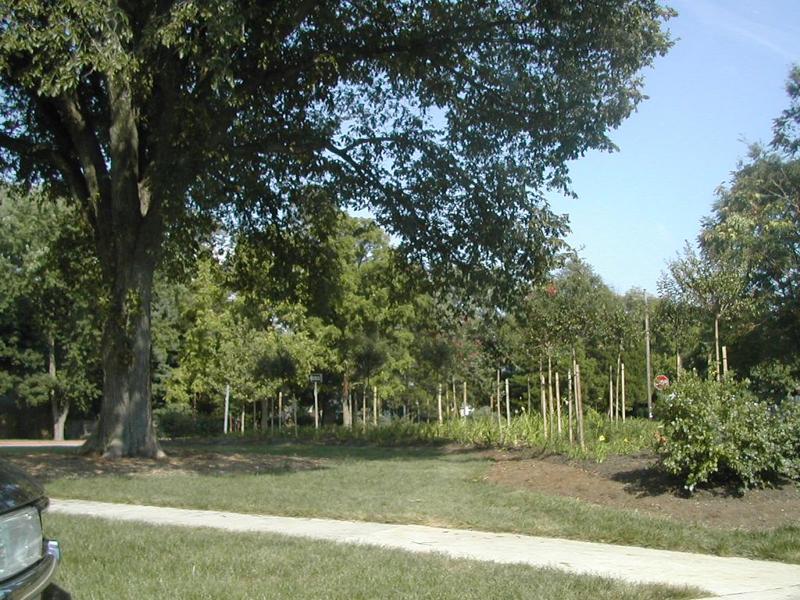

Supplement: S2 Data — Images used in our study. (ZIP) [file pone.0114572.s002.zip › Stimuli/MDS600X800/MDS130.jpg]

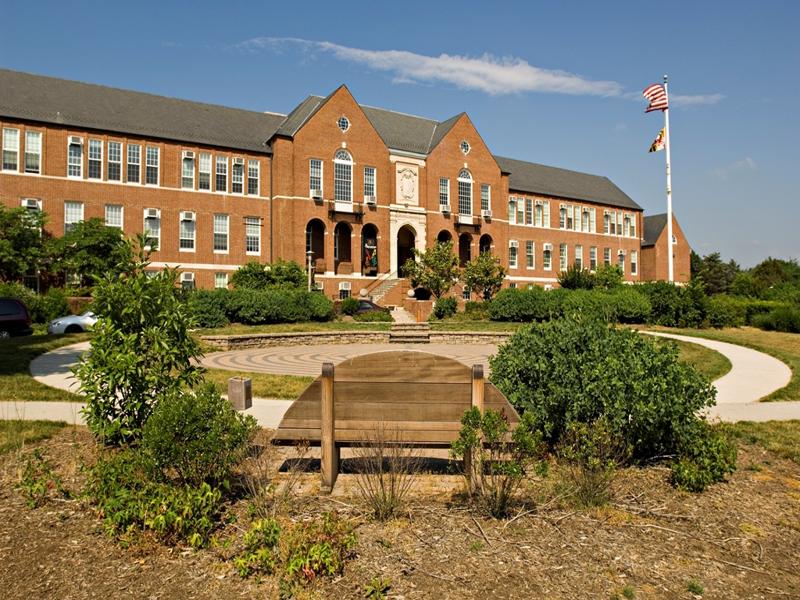

Supplement: S2 Data — Images used in our study. (ZIP) [file pone.0114572.s002.zip › Stimuli/MDS600X800/MDS131.jpg]

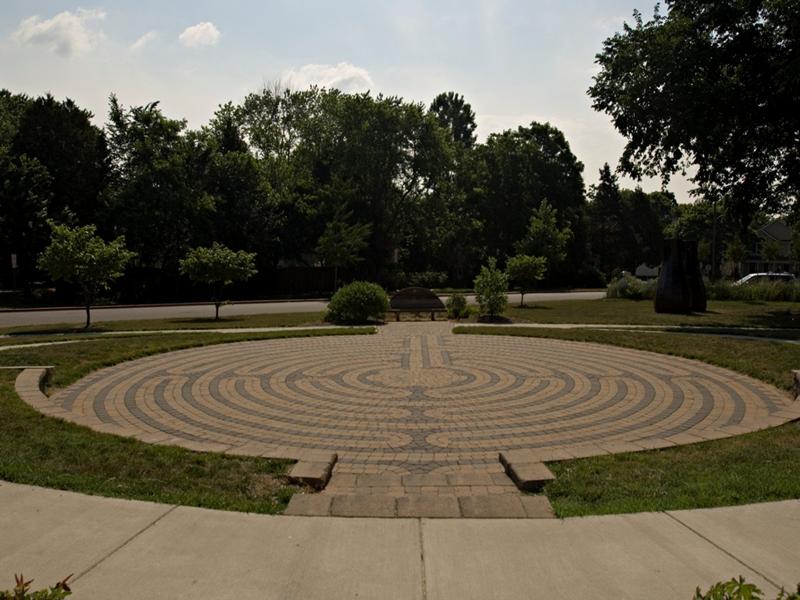

Supplement: S2 Data — Images used in our study. (ZIP) [file pone.0114572.s002.zip › Stimuli/MDS600X800/MDS132.jpg]

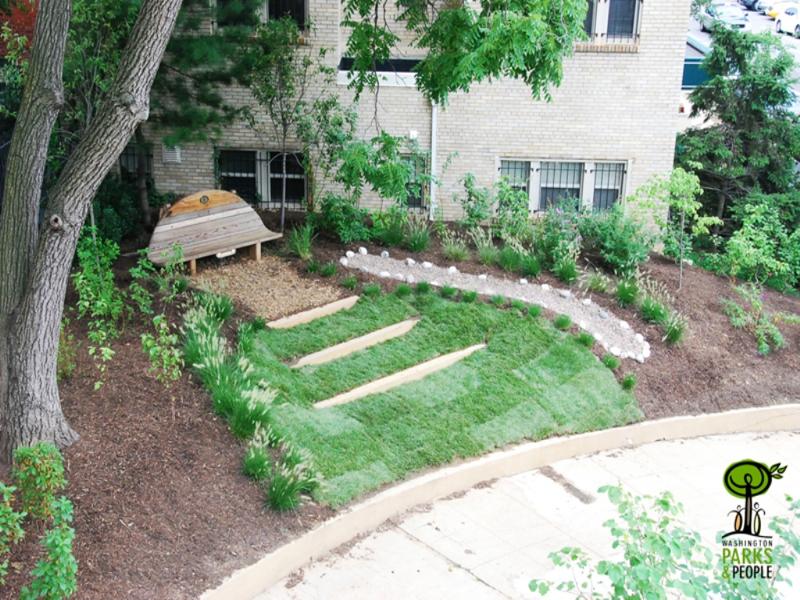

Supplement: S2 Data — Images used in our study. (ZIP) [file pone.0114572.s002.zip › Stimuli/MDS600X800/MDS133.jpg]

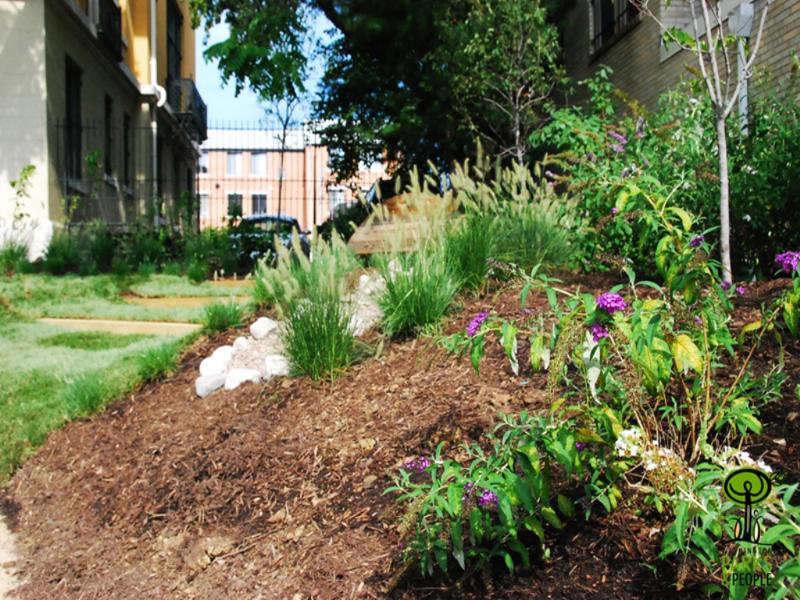

Supplement: S2 Data — Images used in our study. (ZIP) [file pone.0114572.s002.zip › Stimuli/MDS600X800/MDS134.jpg]

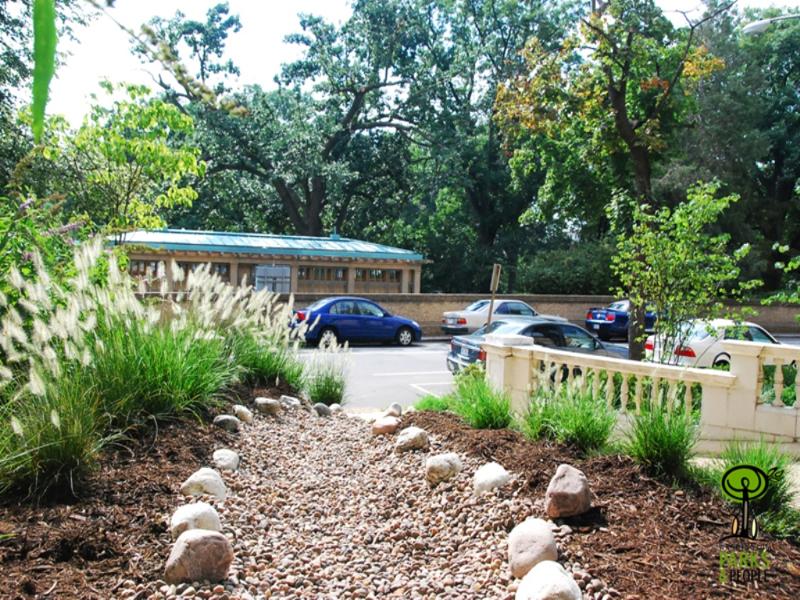

Supplement: S2 Data — Images used in our study. (ZIP) [file pone.0114572.s002.zip › Stimuli/MDS600X800/MDS135.jpg]

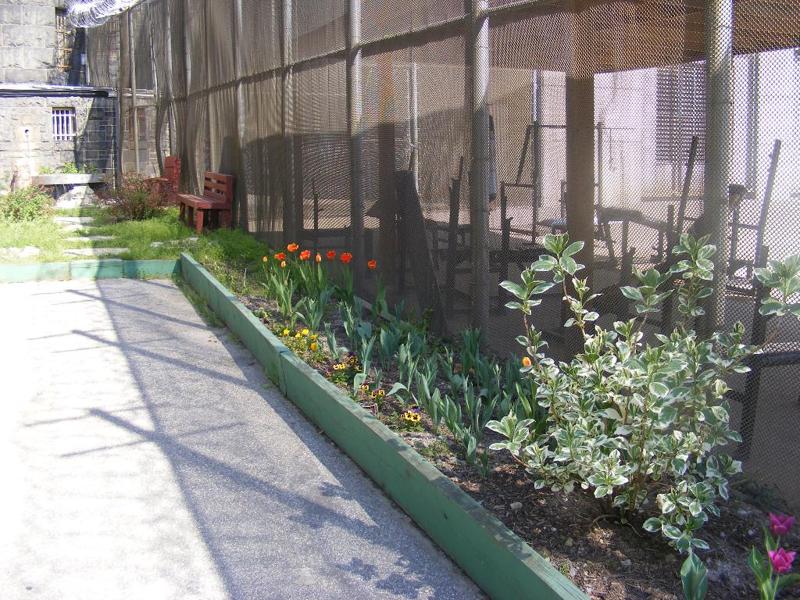

Supplement: S2 Data — Images used in our study. (ZIP) [file pone.0114572.s002.zip › Stimuli/MDS600X800/MDS136.jpg]

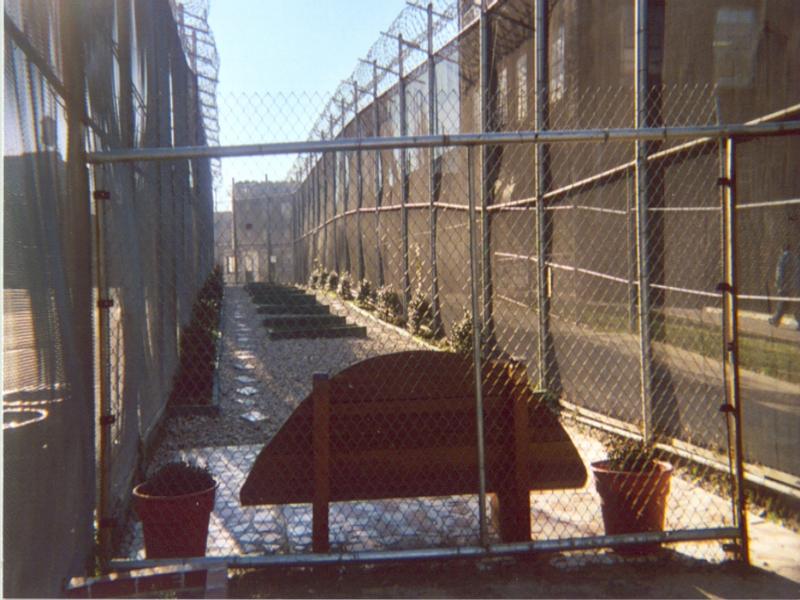

Supplement: S2 Data — Images used in our study. (ZIP) [file pone.0114572.s002.zip › Stimuli/MDS600X800/MDS137.jpg]

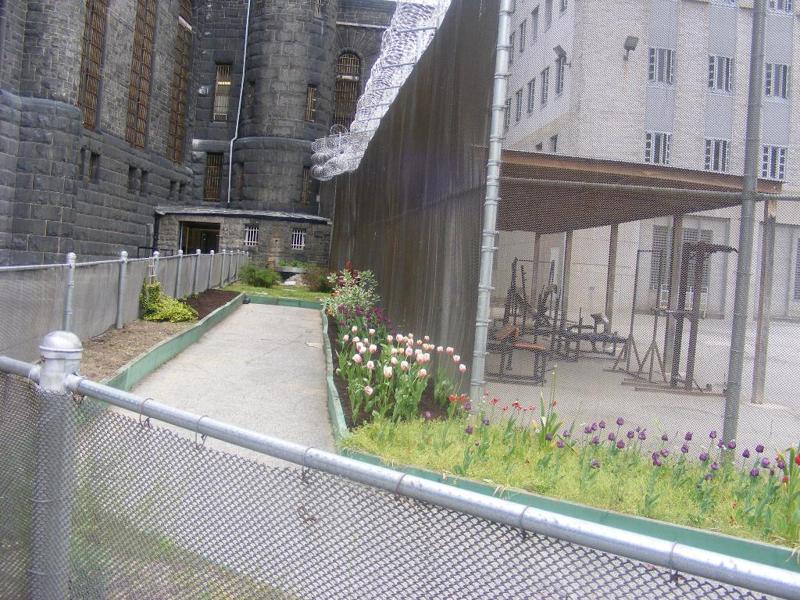

Supplement: S2 Data — Images used in our study. (ZIP) [file pone.0114572.s002.zip › Stimuli/MDS600X800/MDS138.jpg]

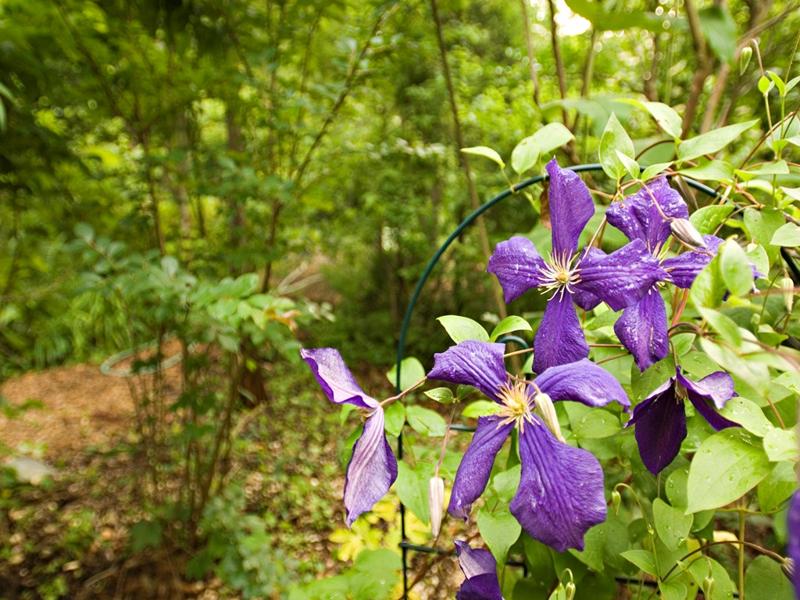

Supplement: S2 Data — Images used in our study. (ZIP) [file pone.0114572.s002.zip › Stimuli/MDS600X800/MDS139.jpg]

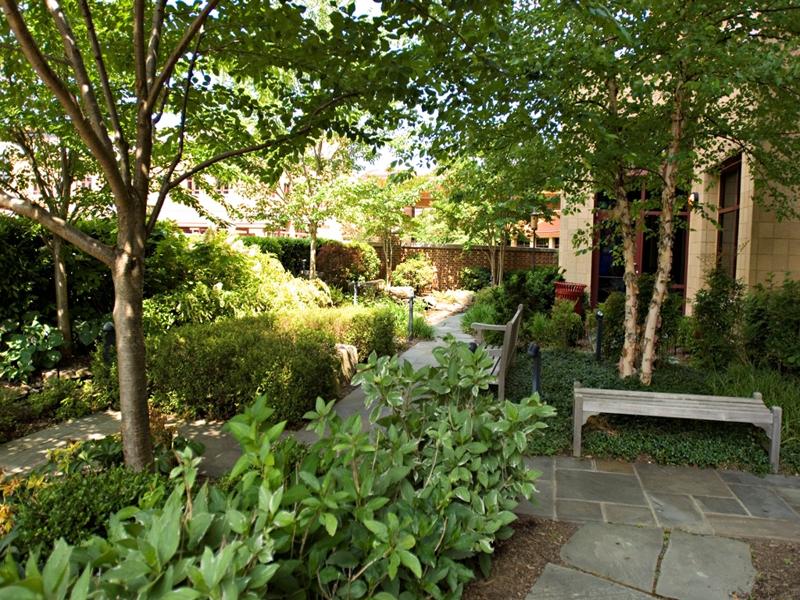

Supplement: S2 Data — Images used in our study. (ZIP) [file pone.0114572.s002.zip › Stimuli/MDS600X800/MDS14.jpg]

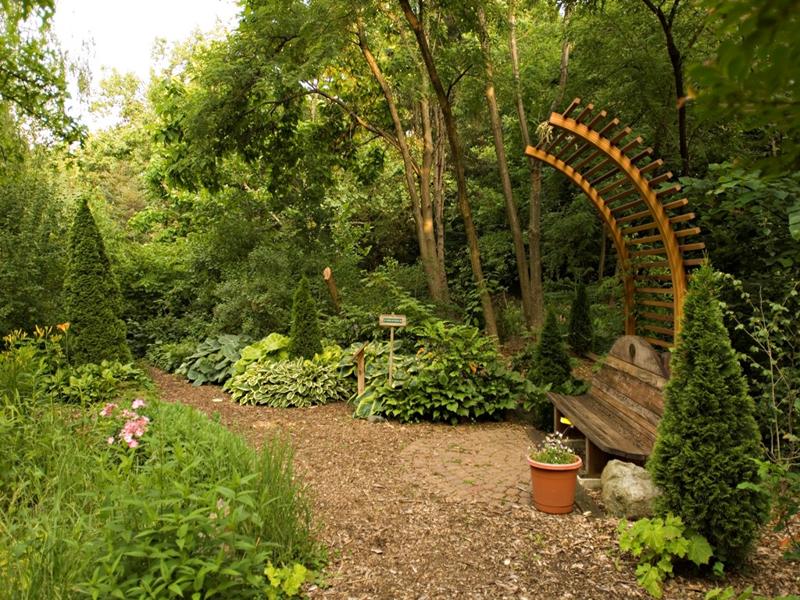

Supplement: S2 Data — Images used in our study. (ZIP) [file pone.0114572.s002.zip › Stimuli/MDS600X800/MDS140.jpg]

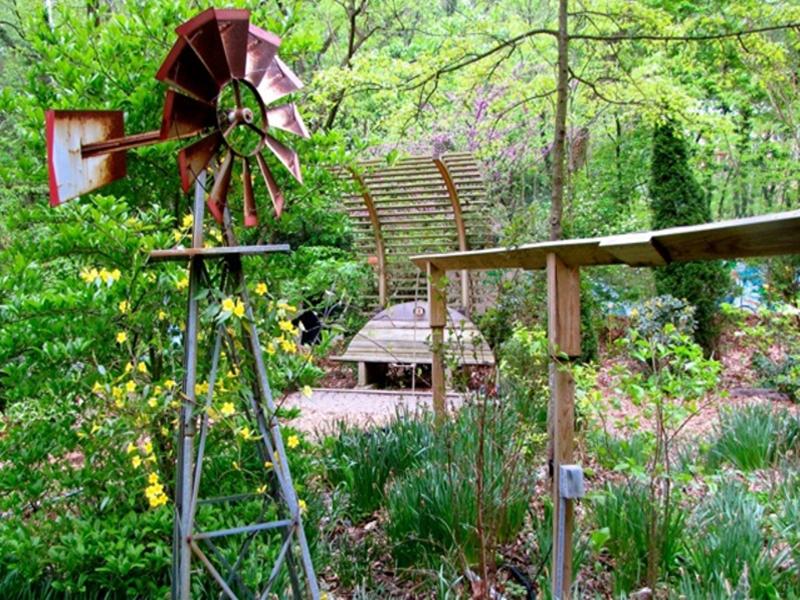

Supplement: S2 Data — Images used in our study. (ZIP) [file pone.0114572.s002.zip › Stimuli/MDS600X800/MDS141.jpg]

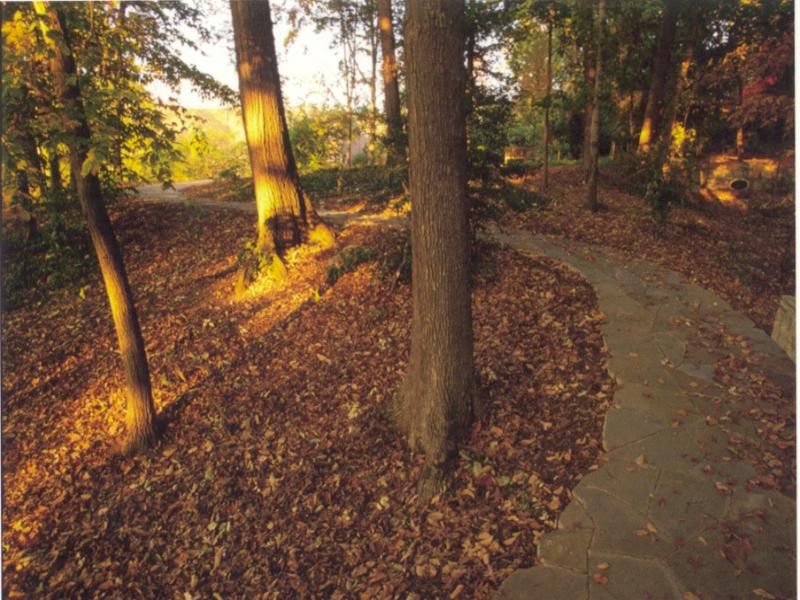

Supplement: S2 Data — Images used in our study. (ZIP) [file pone.0114572.s002.zip › Stimuli/MDS600X800/MDS142.jpg]

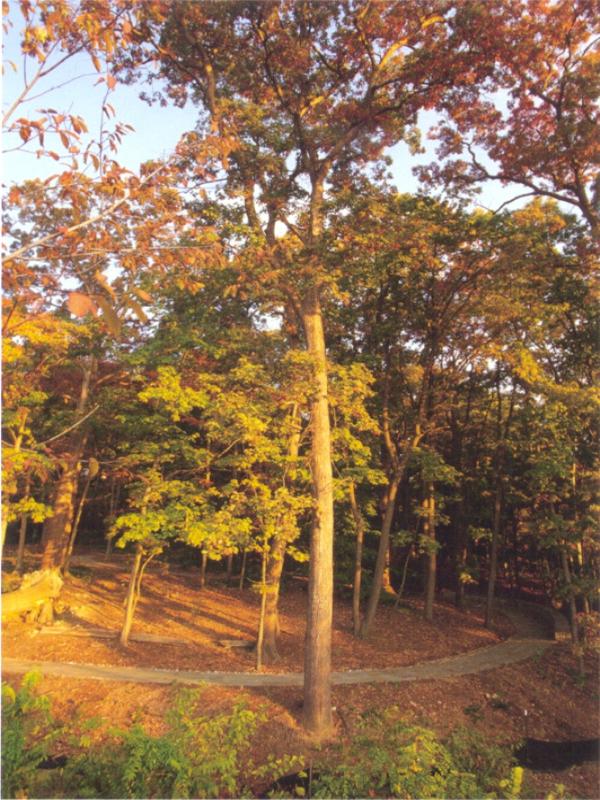

Supplement: S2 Data — Images used in our study. (ZIP) [file pone.0114572.s002.zip › Stimuli/MDS600X800/MDS143.jpg]

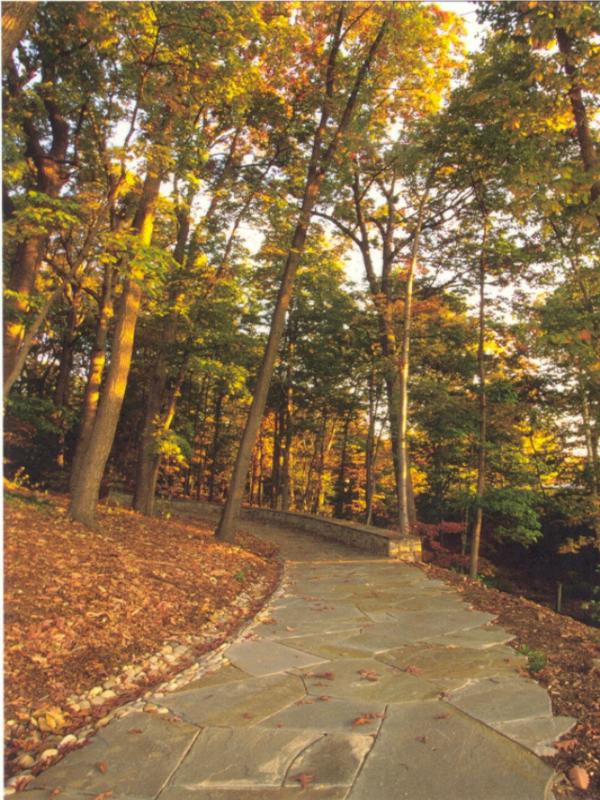

Supplement: S2 Data — Images used in our study. (ZIP) [file pone.0114572.s002.zip › Stimuli/MDS600X800/MDS144.jpg]

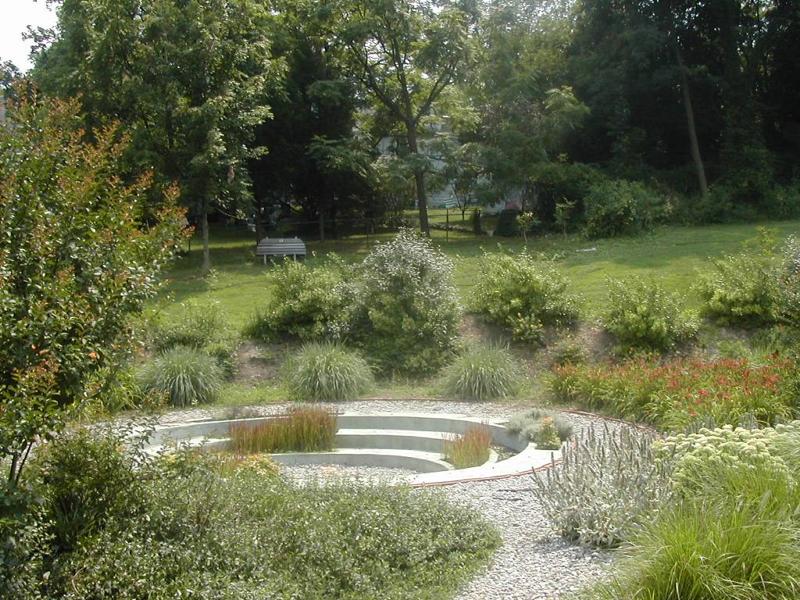

Supplement: S2 Data — Images used in our study. (ZIP) [file pone.0114572.s002.zip › Stimuli/MDS600X800/MDS145.jpg]

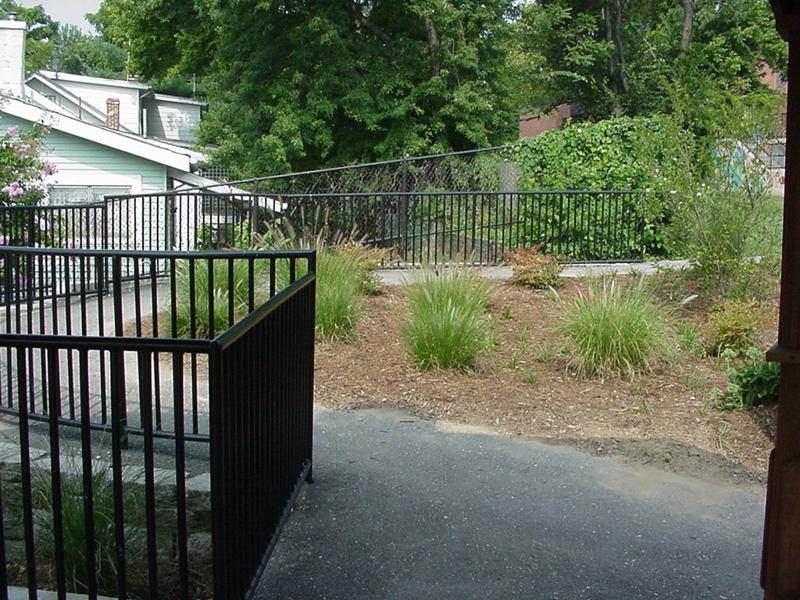

Supplement: S2 Data — Images used in our study. (ZIP) [file pone.0114572.s002.zip › Stimuli/MDS600X800/MDS146.jpg]

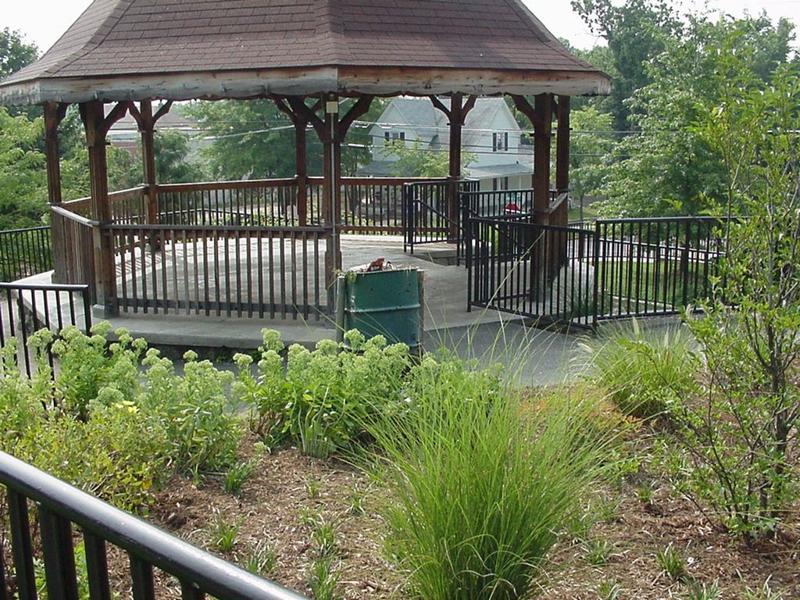

Supplement: S2 Data — Images used in our study. (ZIP) [file pone.0114572.s002.zip › Stimuli/MDS600X800/MDS147.jpg]

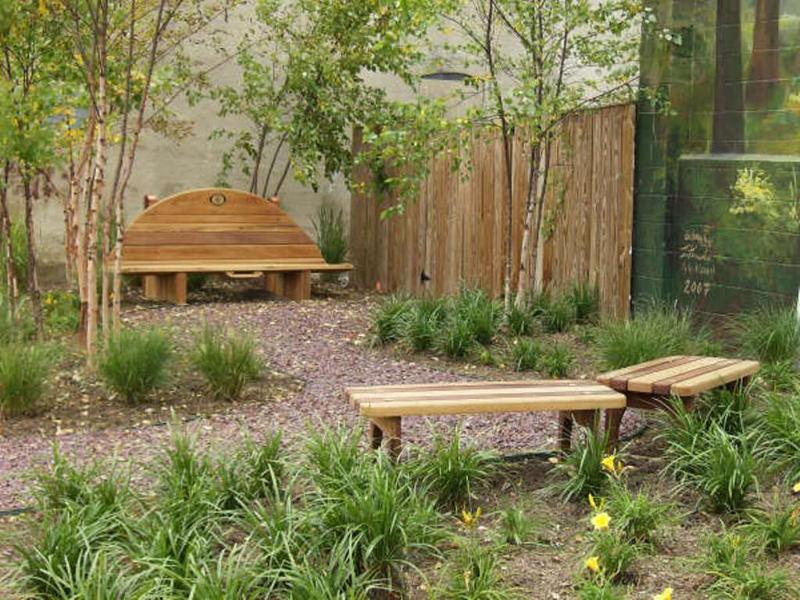

Supplement: S2 Data — Images used in our study. (ZIP) [file pone.0114572.s002.zip › Stimuli/MDS600X800/MDS148.jpg]

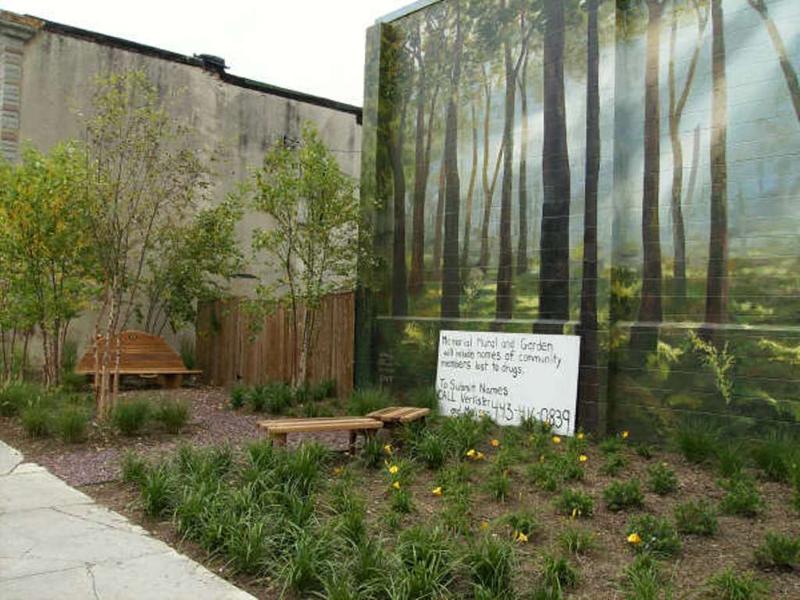

Supplement: S2 Data — Images used in our study. (ZIP) [file pone.0114572.s002.zip › Stimuli/MDS600X800/MDS149.jpg]

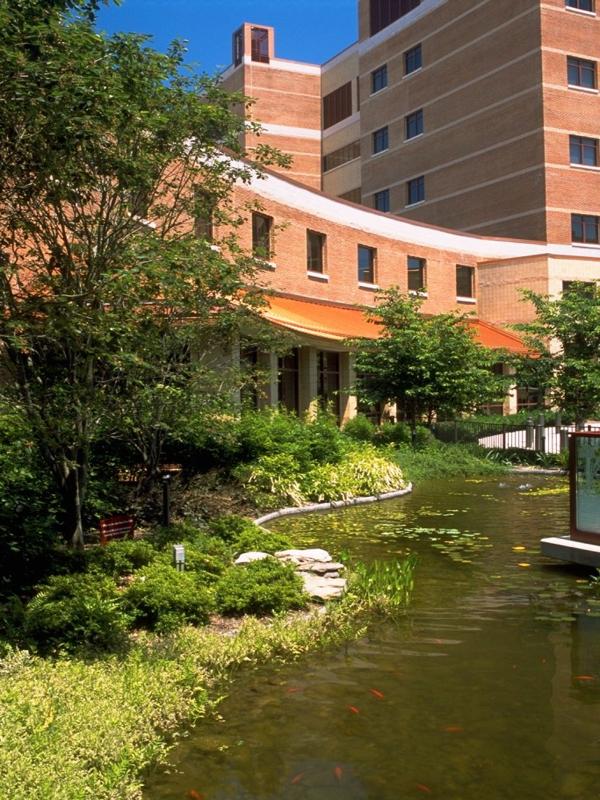

Supplement: S2 Data — Images used in our study. (ZIP) [file pone.0114572.s002.zip › Stimuli/MDS600X800/MDS15.jpg]

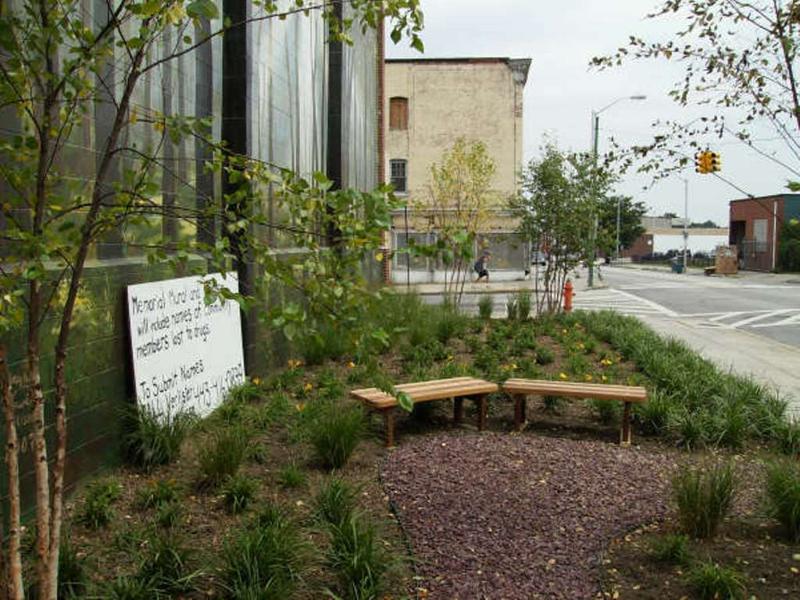

Supplement: S2 Data — Images used in our study. (ZIP) [file pone.0114572.s002.zip › Stimuli/MDS600X800/MDS150.jpg]

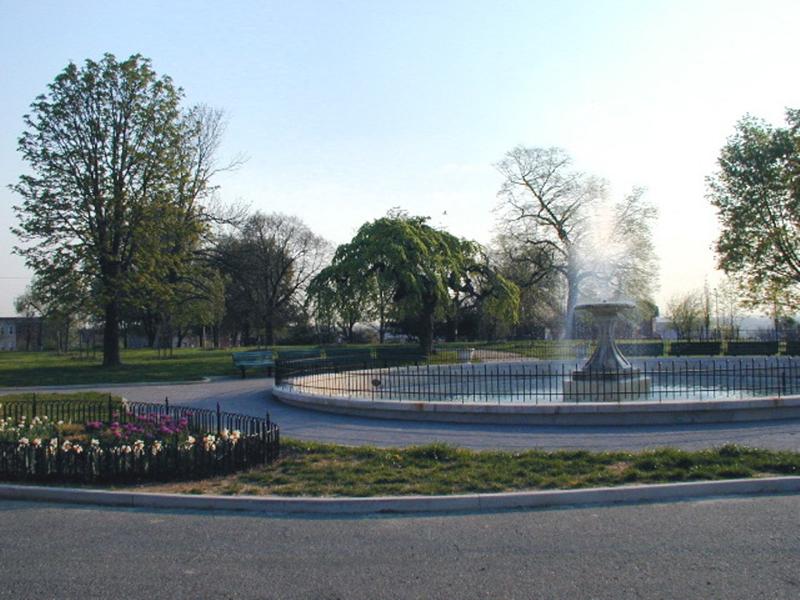

Supplement: S2 Data — Images used in our study. (ZIP) [file pone.0114572.s002.zip › Stimuli/MDS600X800/MDS151.jpg]

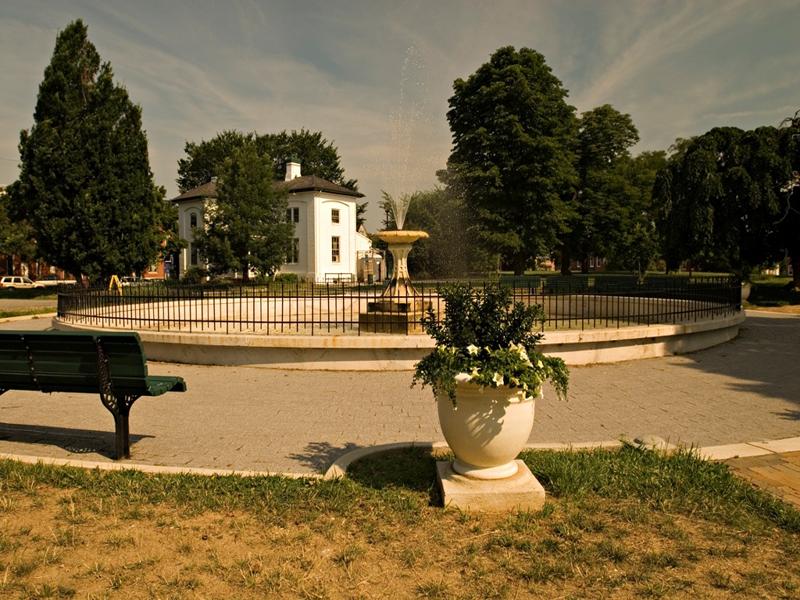

Supplement: S2 Data — Images used in our study. (ZIP) [file pone.0114572.s002.zip › Stimuli/MDS600X800/MDS152.jpg]

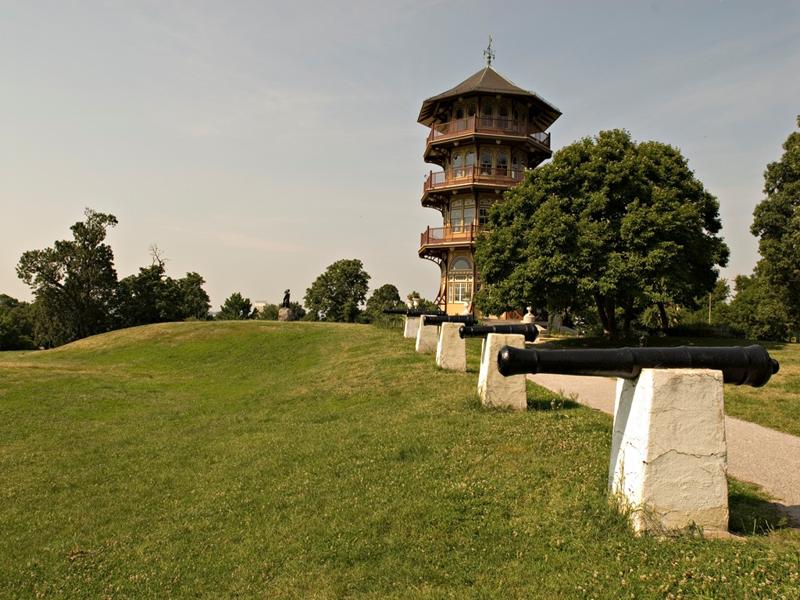

Supplement: S2 Data — Images used in our study. (ZIP) [file pone.0114572.s002.zip › Stimuli/MDS600X800/MDS153.jpg]

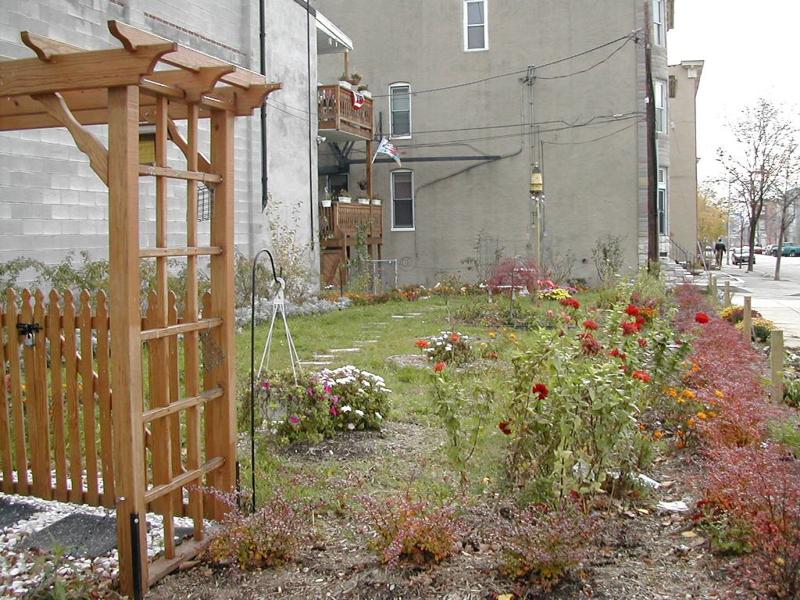

Supplement: S2 Data — Images used in our study. (ZIP) [file pone.0114572.s002.zip › Stimuli/MDS600X800/MDS154.jpg]

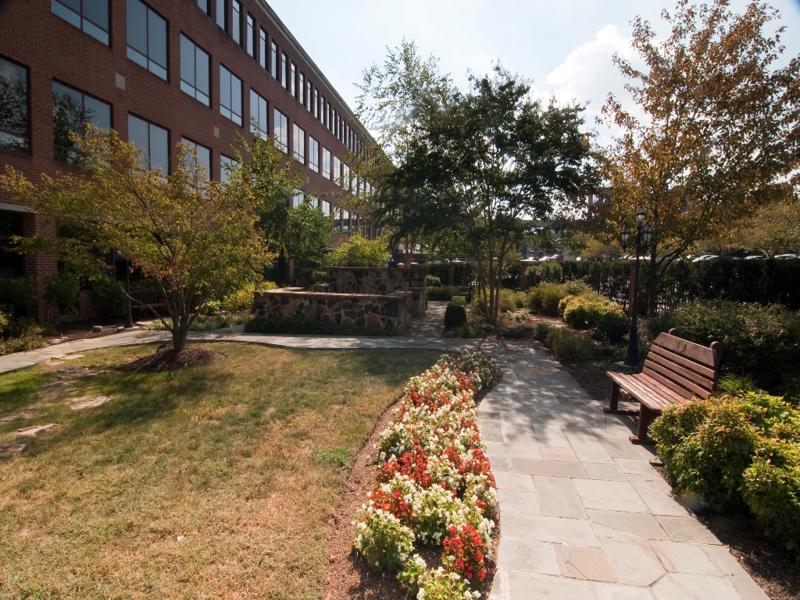

Supplement: S2 Data — Images used in our study. (ZIP) [file pone.0114572.s002.zip › Stimuli/MDS600X800/MDS155.jpg]

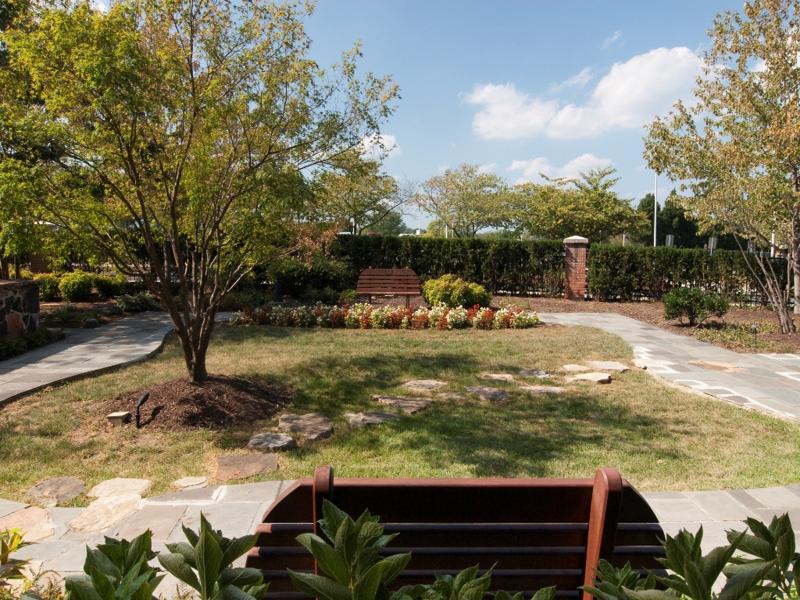

Supplement: S2 Data — Images used in our study. (ZIP) [file pone.0114572.s002.zip › Stimuli/MDS600X800/MDS156.jpg]

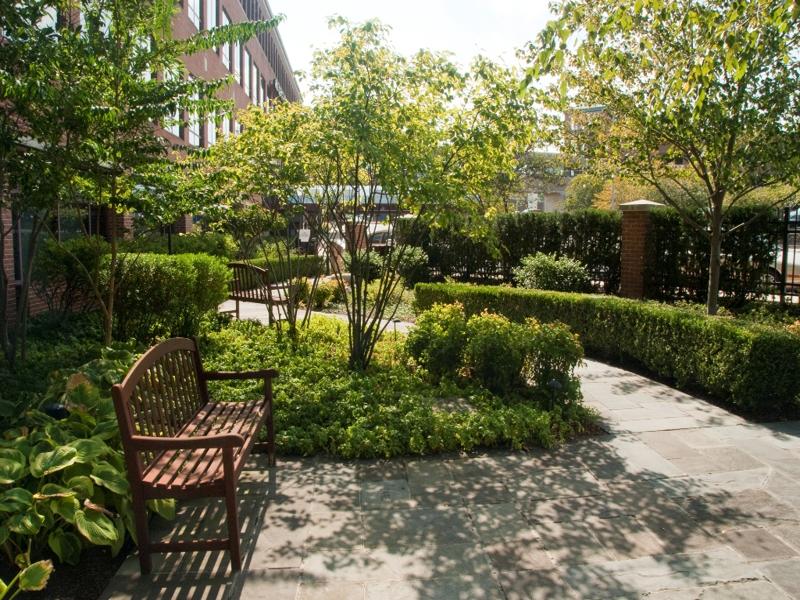

Supplement: S2 Data — Images used in our study. (ZIP) [file pone.0114572.s002.zip › Stimuli/MDS600X800/MDS157.jpg]

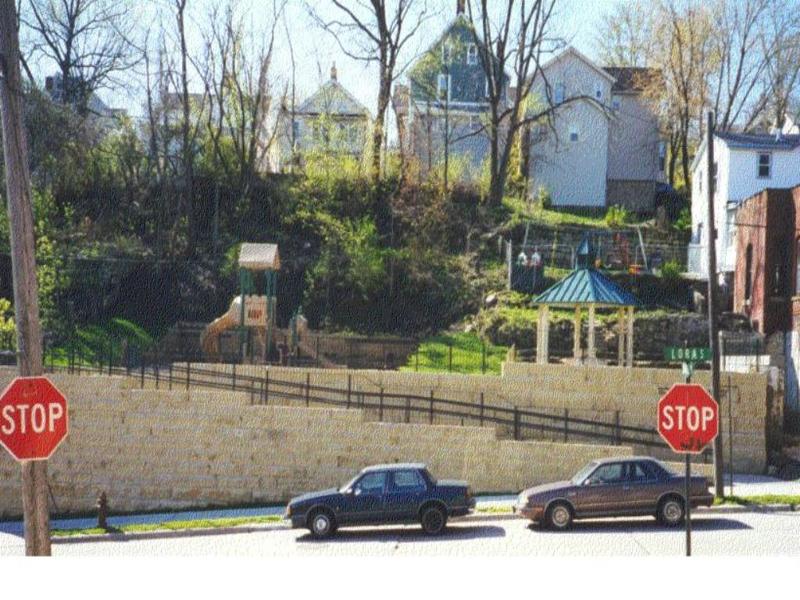

Supplement: S2 Data — Images used in our study. (ZIP) [file pone.0114572.s002.zip › Stimuli/MDS600X800/MDS158.jpg]

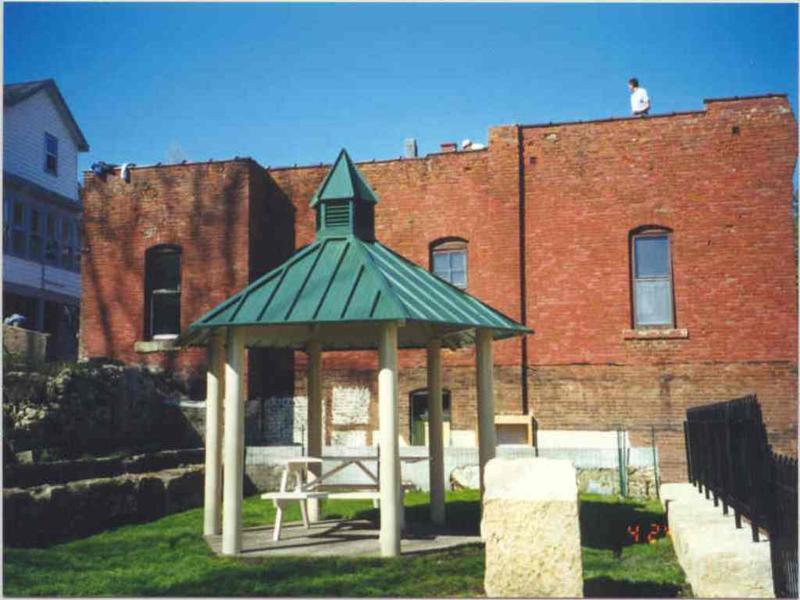

Supplement: S2 Data — Images used in our study. (ZIP) [file pone.0114572.s002.zip › Stimuli/MDS600X800/MDS159.jpg]

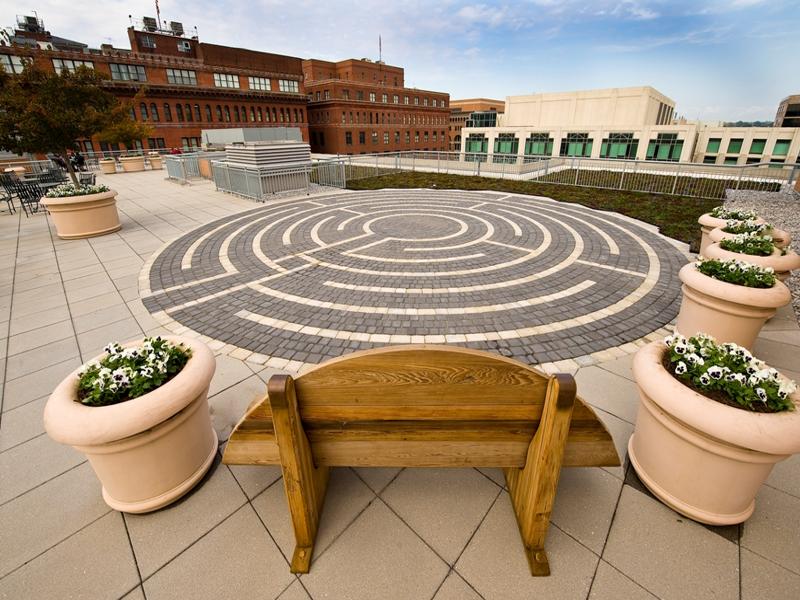

Supplement: S2 Data — Images used in our study. (ZIP) [file pone.0114572.s002.zip › Stimuli/MDS600X800/MDS16.jpg]

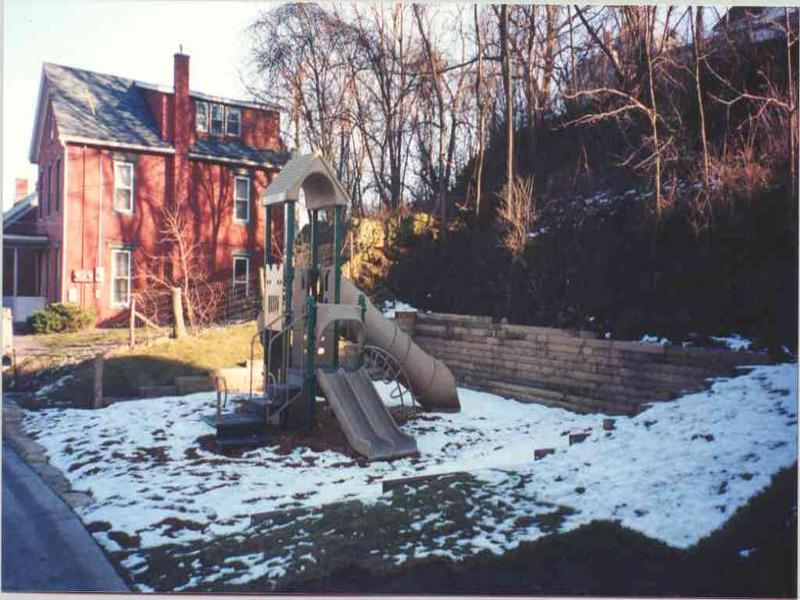

Supplement: S2 Data — Images used in our study. (ZIP) [file pone.0114572.s002.zip › Stimuli/MDS600X800/MDS160.jpg]

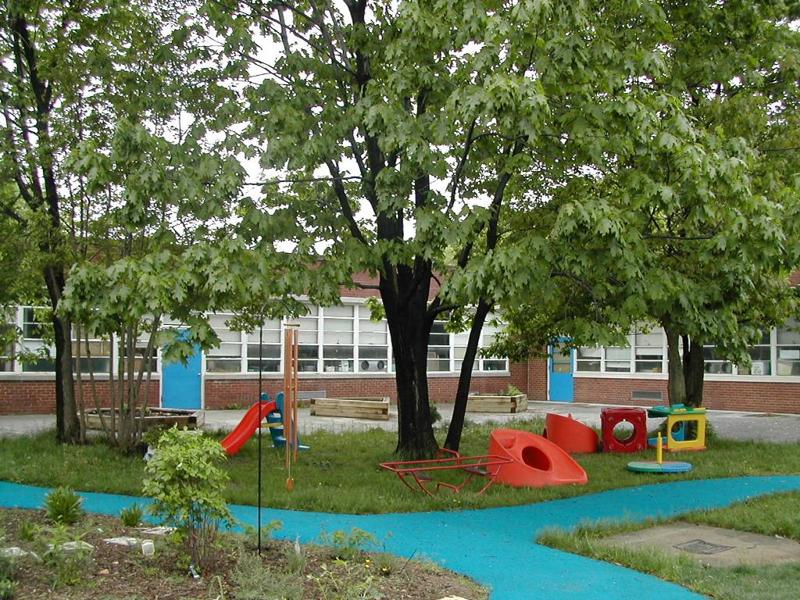

Supplement: S2 Data — Images used in our study. (ZIP) [file pone.0114572.s002.zip › Stimuli/MDS600X800/MDS161.jpg]

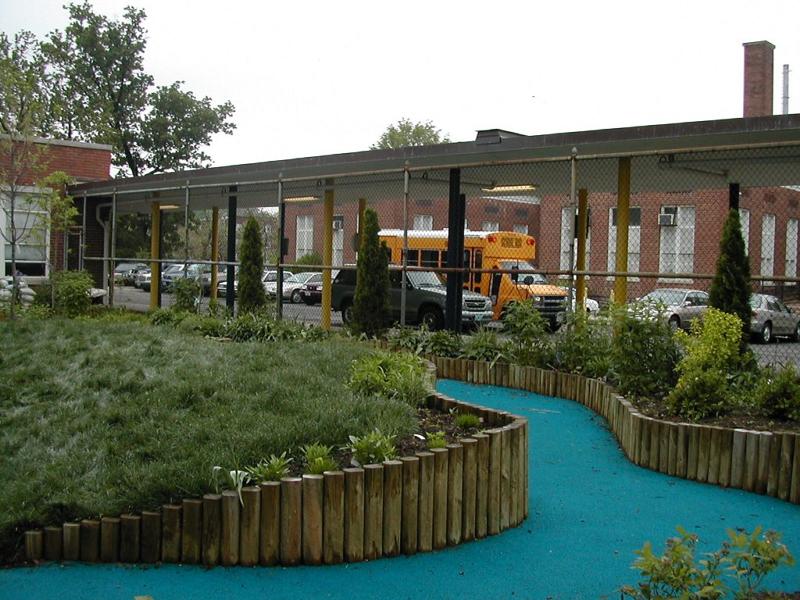

Supplement: S2 Data — Images used in our study. (ZIP) [file pone.0114572.s002.zip › Stimuli/MDS600X800/MDS162.jpg]

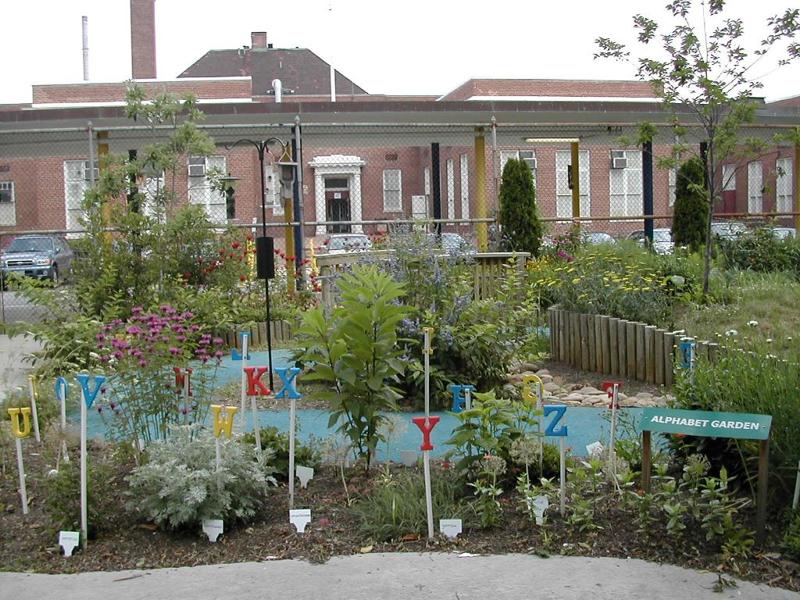

Supplement: S2 Data — Images used in our study. (ZIP) [file pone.0114572.s002.zip › Stimuli/MDS600X800/MDS163.jpg]

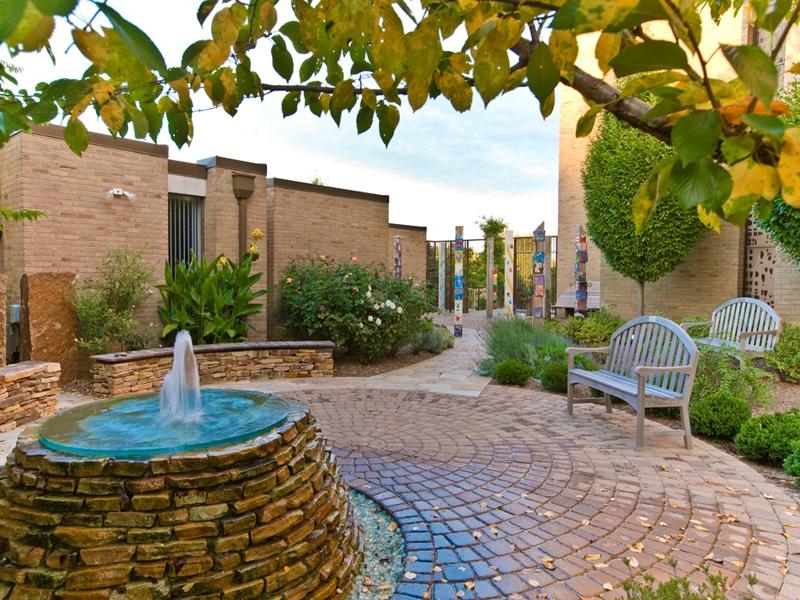

Supplement: S2 Data — Images used in our study. (ZIP) [file pone.0114572.s002.zip › Stimuli/MDS600X800/MDS164.jpg]

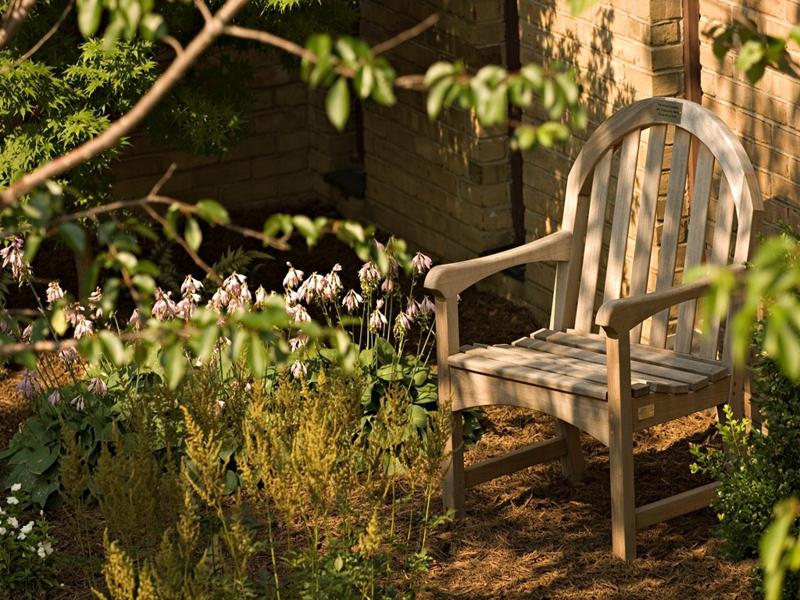

Supplement: S2 Data — Images used in our study. (ZIP) [file pone.0114572.s002.zip › Stimuli/MDS600X800/MDS165.jpg]

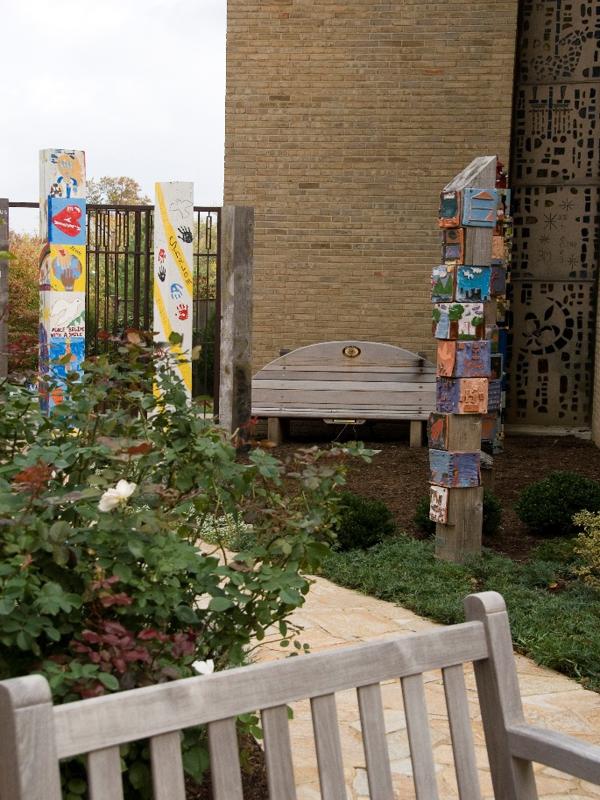

Supplement: S2 Data — Images used in our study. (ZIP) [file pone.0114572.s002.zip › Stimuli/MDS600X800/MDS166.jpg]

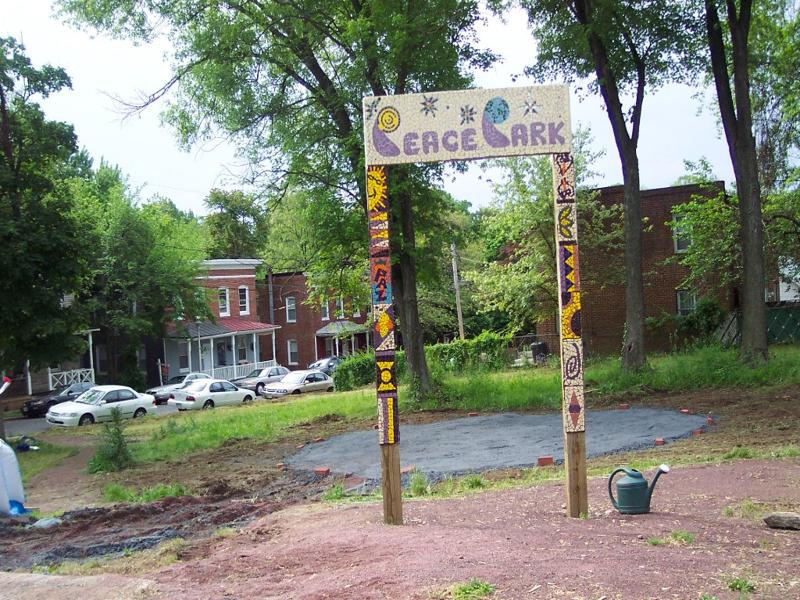

Supplement: S2 Data — Images used in our study. (ZIP) [file pone.0114572.s002.zip › Stimuli/MDS600X800/MDS167.jpg]

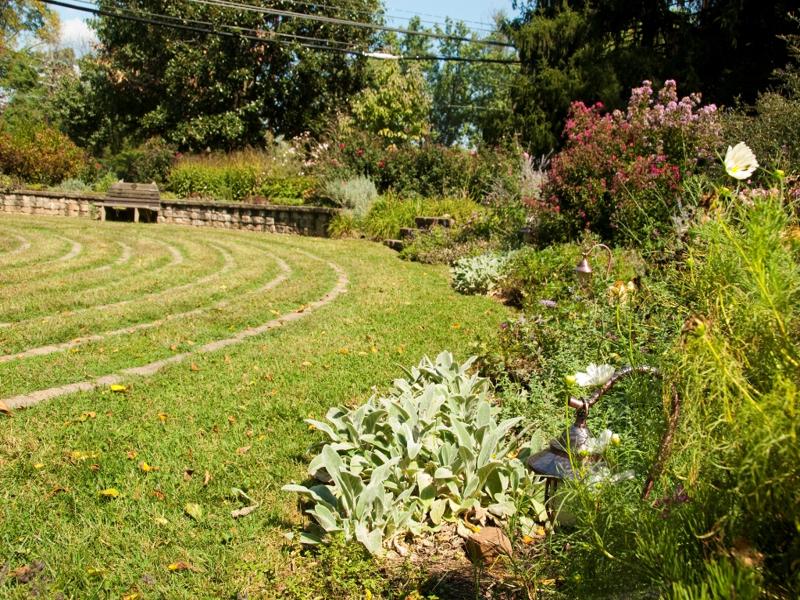

Supplement: S2 Data — Images used in our study. (ZIP) [file pone.0114572.s002.zip › Stimuli/MDS600X800/MDS168.jpg]

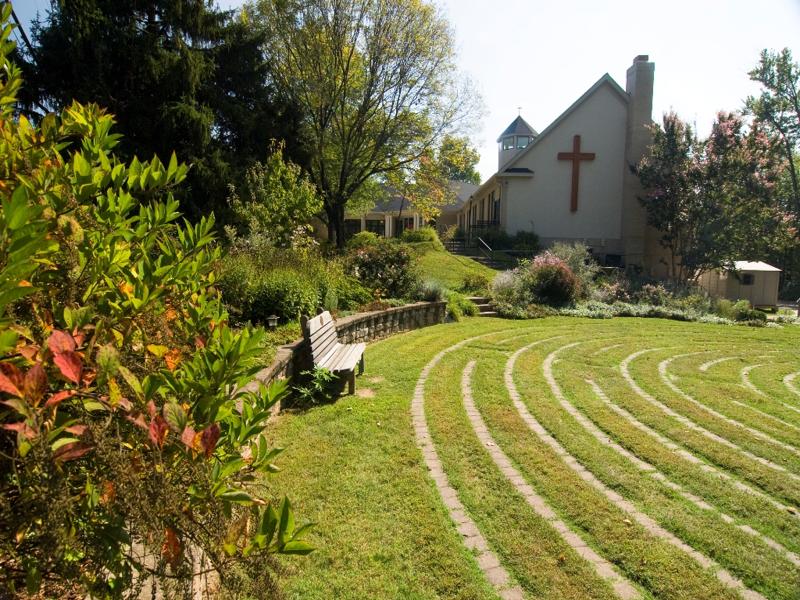

Supplement: S2 Data — Images used in our study. (ZIP) [file pone.0114572.s002.zip › Stimuli/MDS600X800/MDS169.jpg]

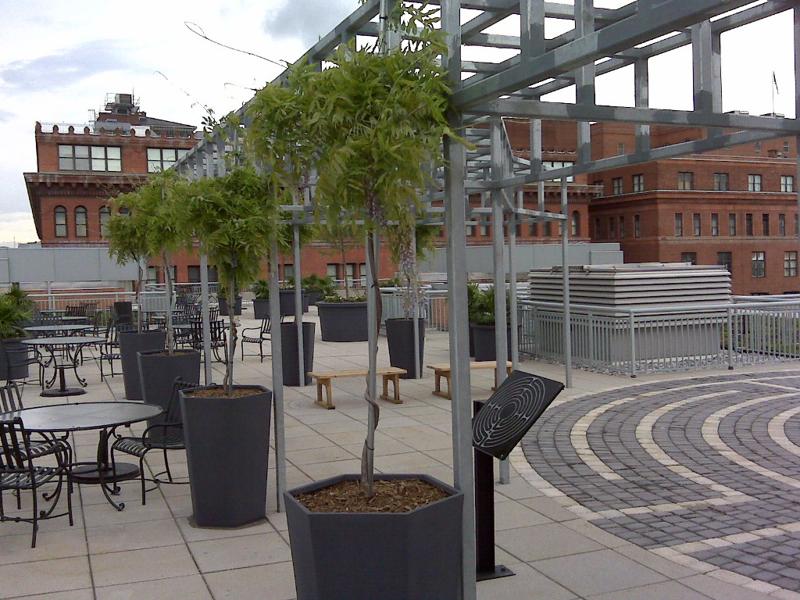

Supplement: S2 Data — Images used in our study. (ZIP) [file pone.0114572.s002.zip › Stimuli/MDS600X800/MDS17.jpg]

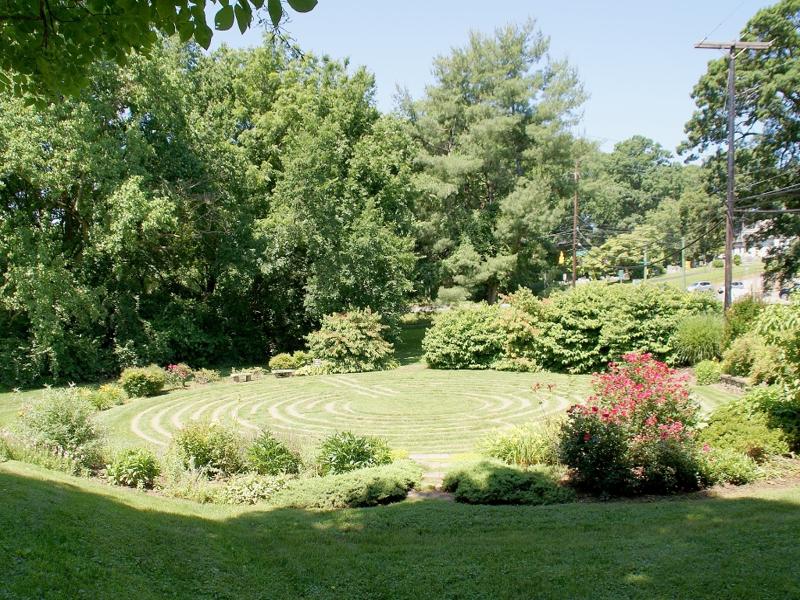

Supplement: S2 Data — Images used in our study. (ZIP) [file pone.0114572.s002.zip › Stimuli/MDS600X800/MDS170.jpg]

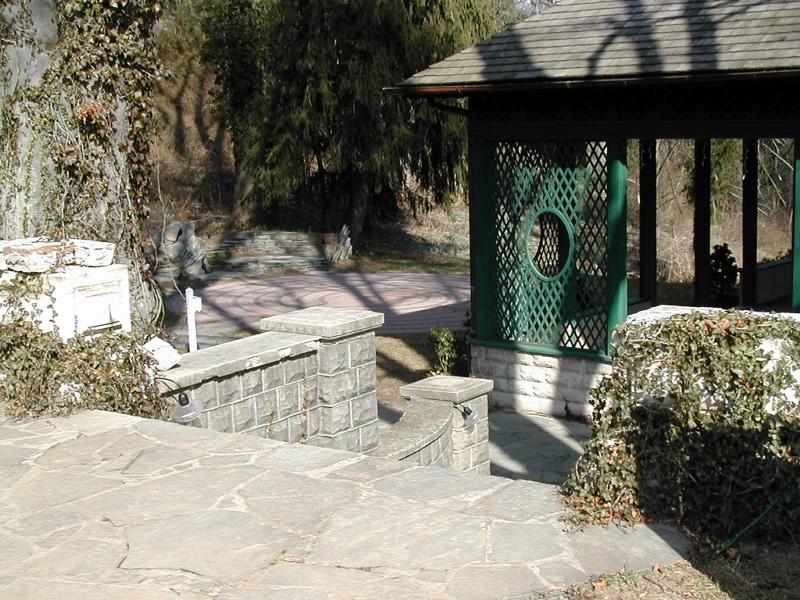

Supplement: S2 Data — Images used in our study. (ZIP) [file pone.0114572.s002.zip › Stimuli/MDS600X800/MDS171.jpg]

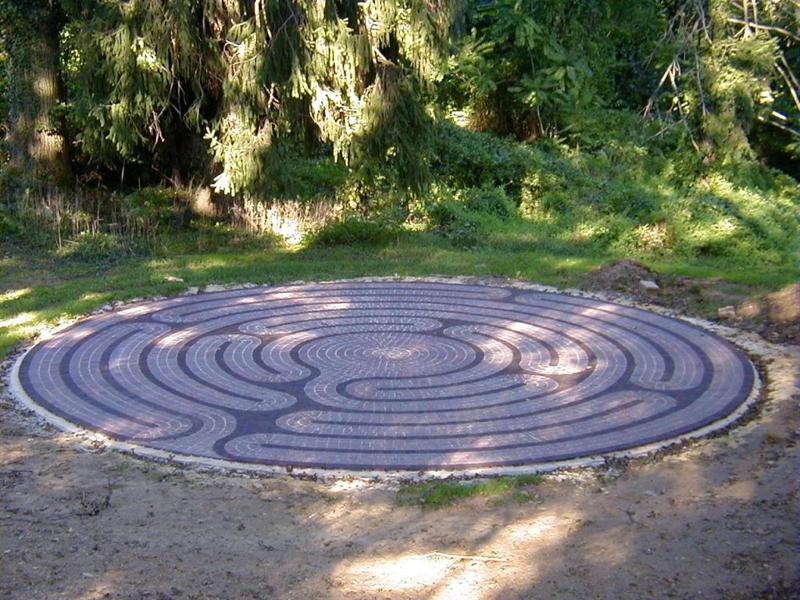

Supplement: S2 Data — Images used in our study. (ZIP) [file pone.0114572.s002.zip › Stimuli/MDS600X800/MDS172.jpg]

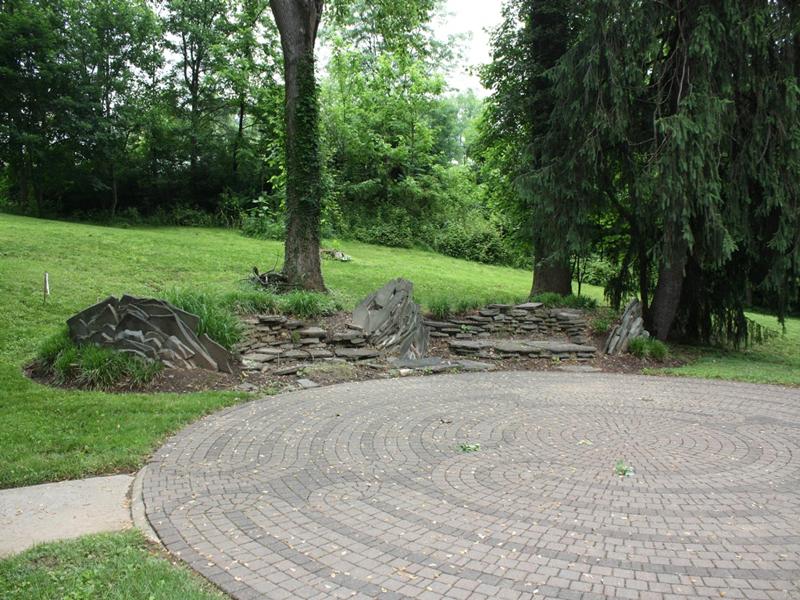

Supplement: S2 Data — Images used in our study. (ZIP) [file pone.0114572.s002.zip › Stimuli/MDS600X800/MDS173.jpg]

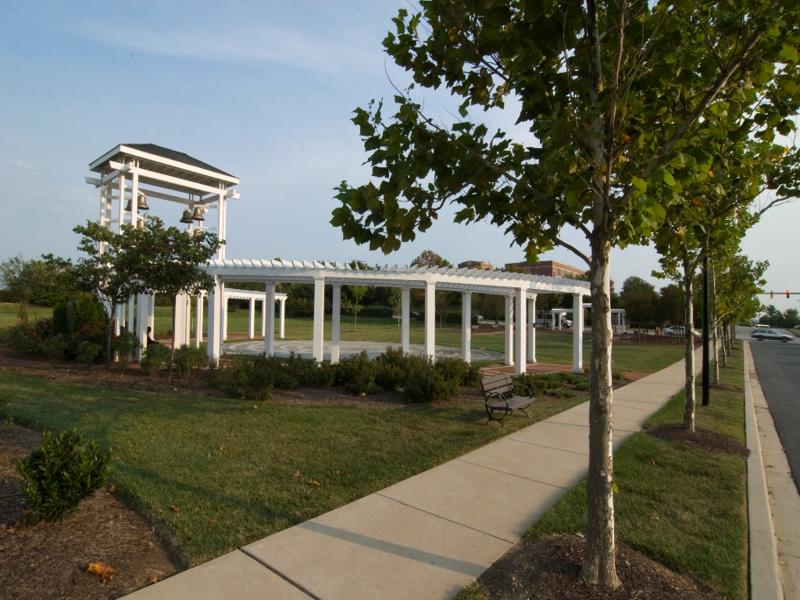

Supplement: S2 Data — Images used in our study. (ZIP) [file pone.0114572.s002.zip › Stimuli/MDS600X800/MDS174.jpg]

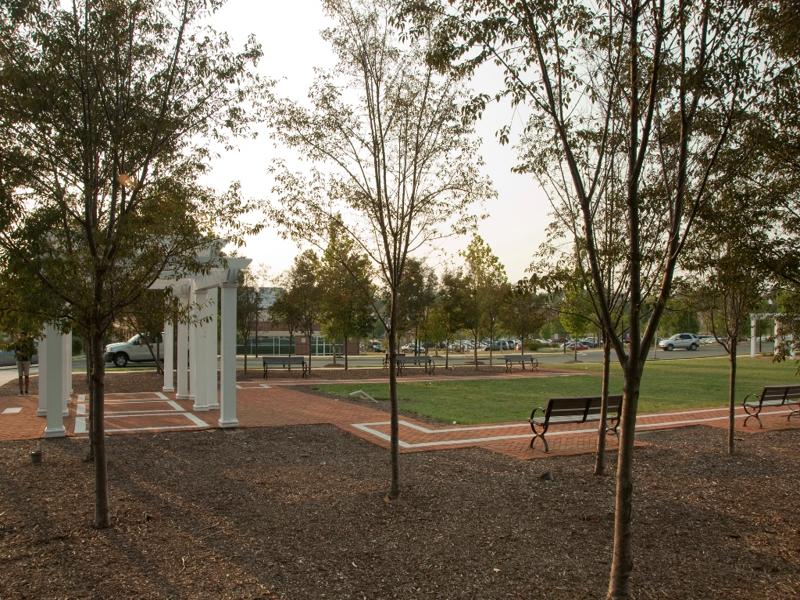

Supplement: S2 Data — Images used in our study. (ZIP) [file pone.0114572.s002.zip › Stimuli/MDS600X800/MDS175.jpg]

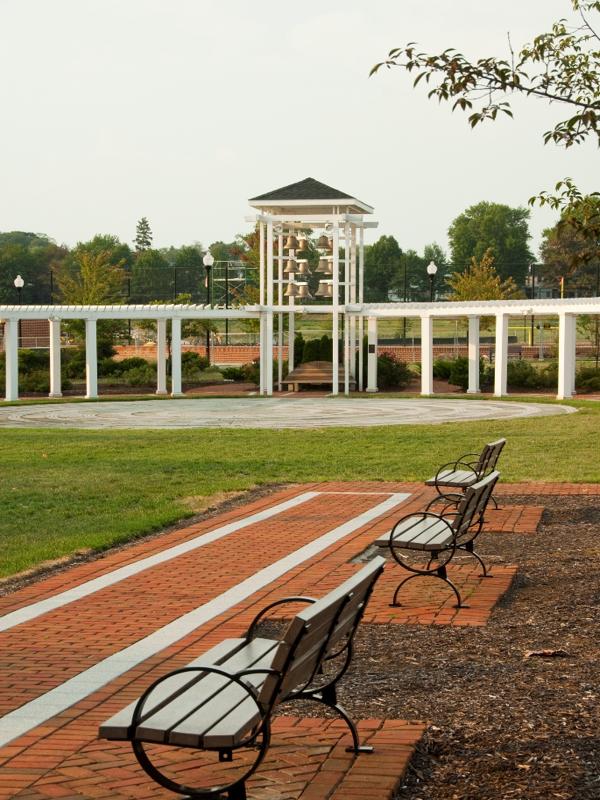

Supplement: S2 Data — Images used in our study. (ZIP) [file pone.0114572.s002.zip › Stimuli/MDS600X800/MDS176.jpg]

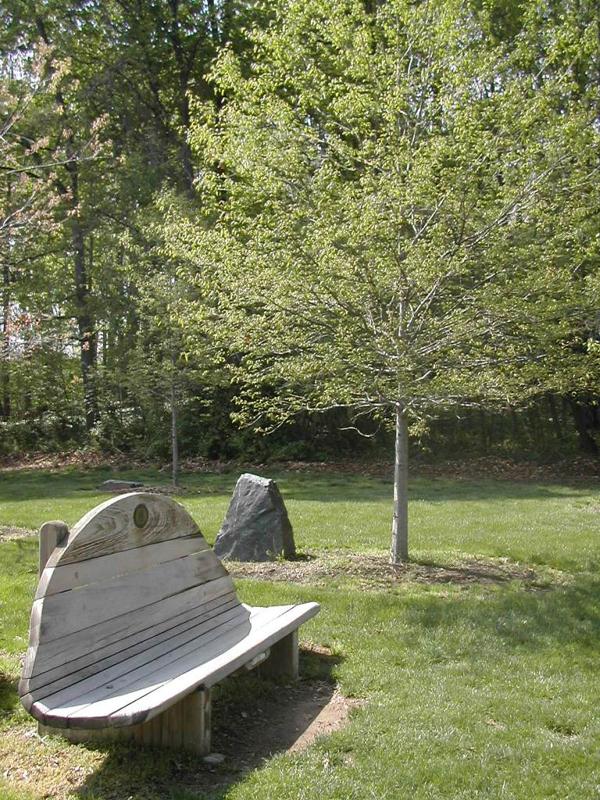

Supplement: S2 Data — Images used in our study. (ZIP) [file pone.0114572.s002.zip › Stimuli/MDS600X800/MDS177.jpg]

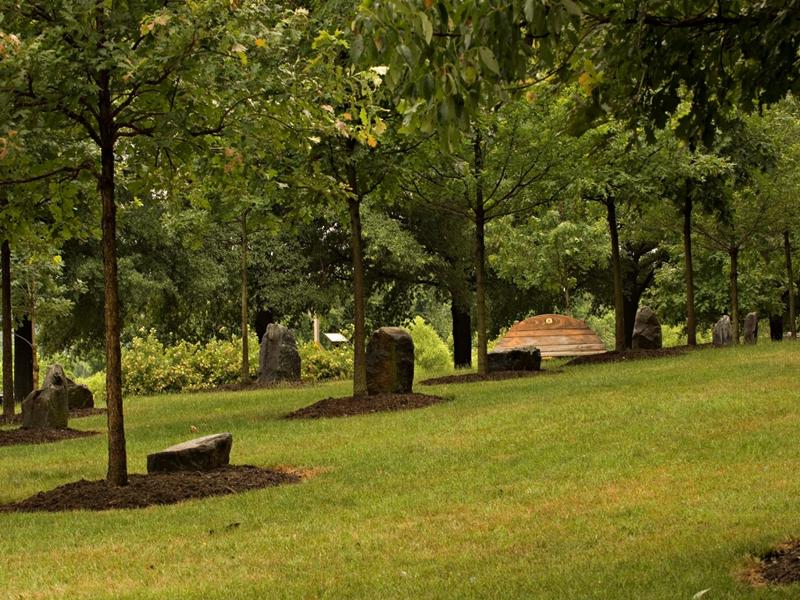

Supplement: S2 Data — Images used in our study. (ZIP) [file pone.0114572.s002.zip › Stimuli/MDS600X800/MDS178.jpg]

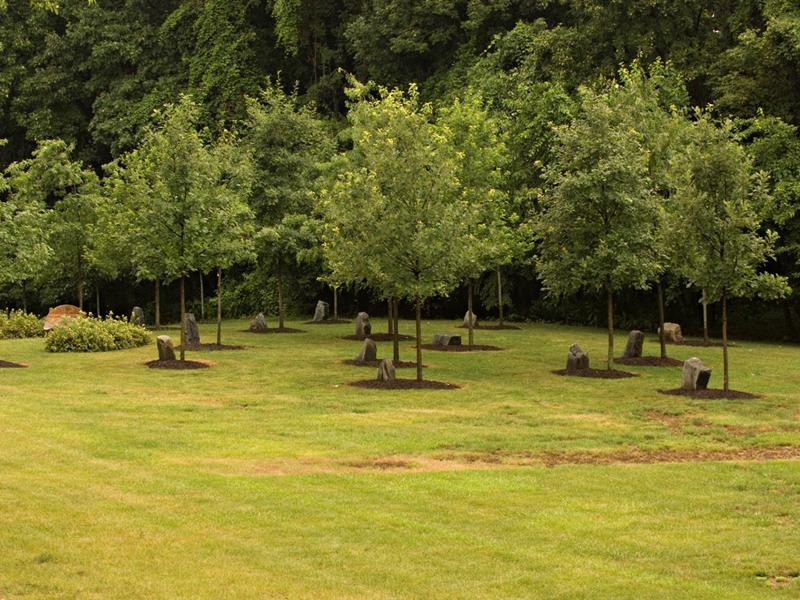

Supplement: S2 Data — Images used in our study. (ZIP) [file pone.0114572.s002.zip › Stimuli/MDS600X800/MDS179.jpg]

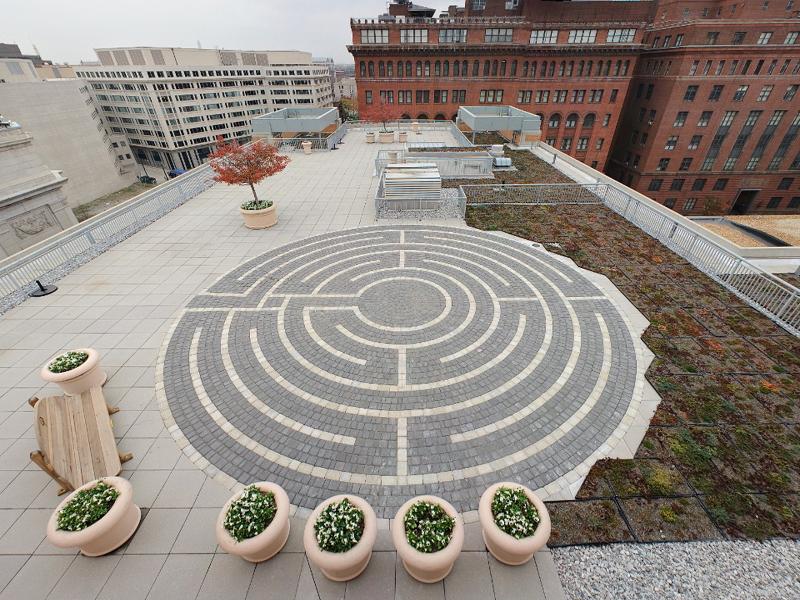

Supplement: S2 Data — Images used in our study. (ZIP) [file pone.0114572.s002.zip › Stimuli/MDS600X800/MDS18.jpg]

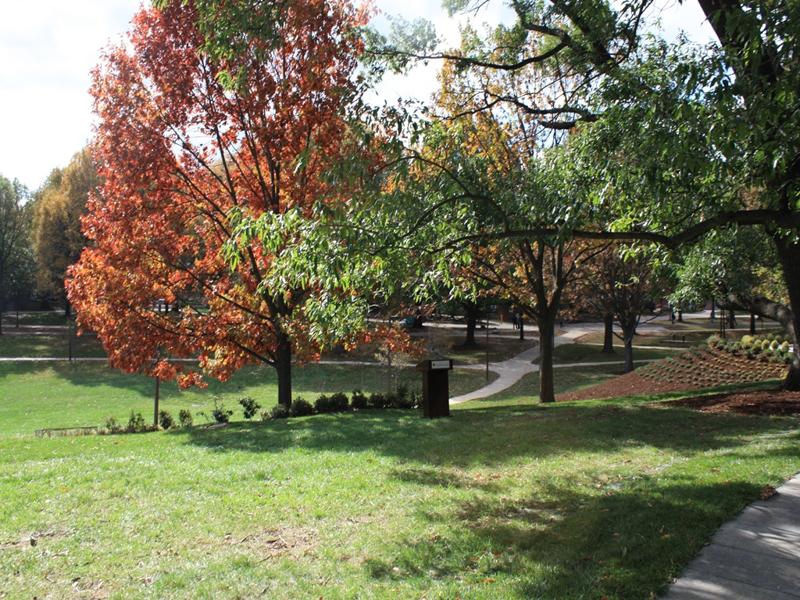

Supplement: S2 Data — Images used in our study. (ZIP) [file pone.0114572.s002.zip › Stimuli/MDS600X800/MDS180.jpg]

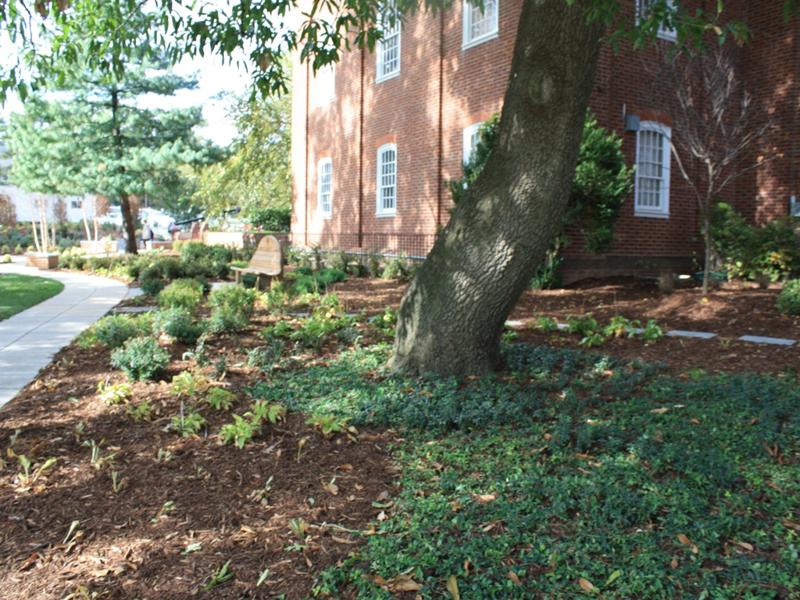

Supplement: S2 Data — Images used in our study. (ZIP) [file pone.0114572.s002.zip › Stimuli/MDS600X800/MDS181.jpg]

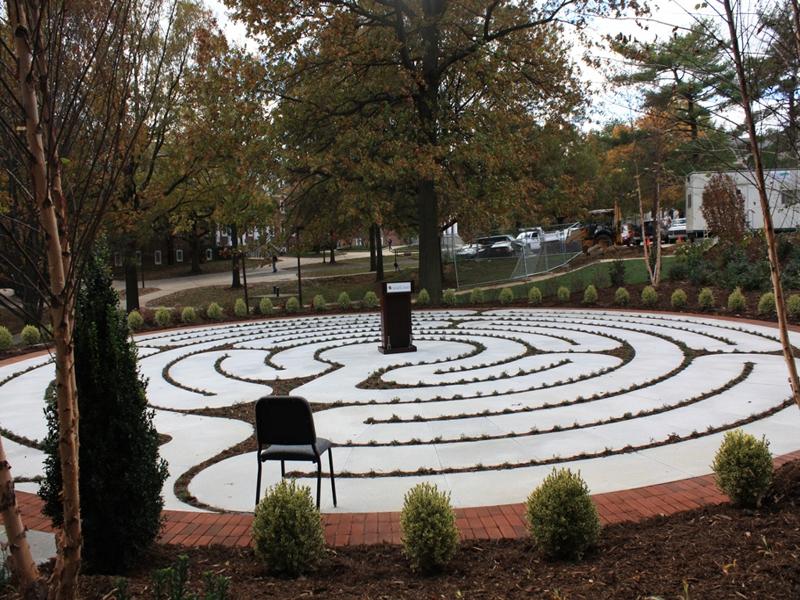

Supplement: S2 Data — Images used in our study. (ZIP) [file pone.0114572.s002.zip › Stimuli/MDS600X800/MDS182.jpg]

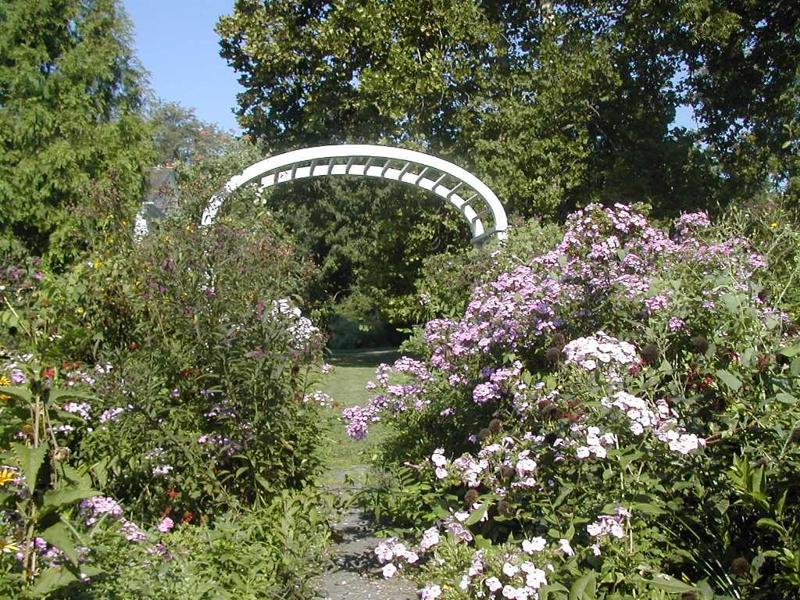

Supplement: S2 Data — Images used in our study. (ZIP) [file pone.0114572.s002.zip › Stimuli/MDS600X800/MDS183.jpg]

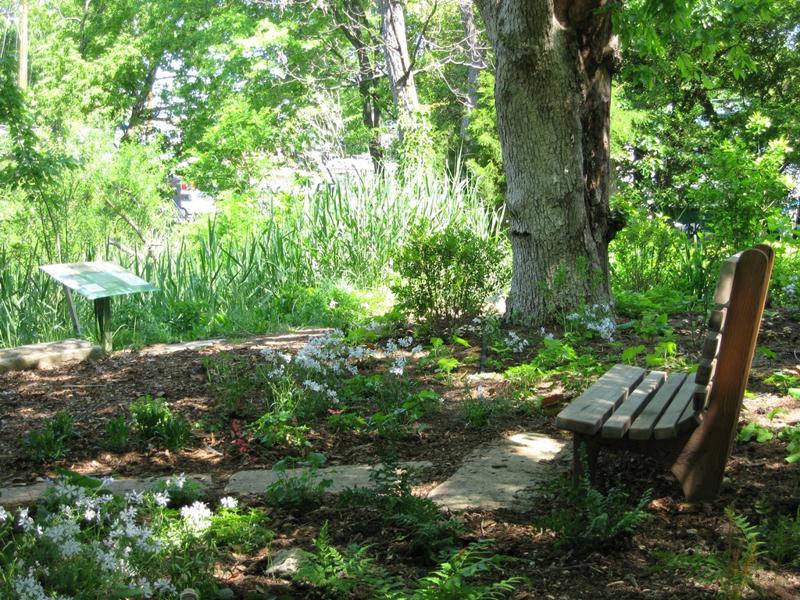

Supplement: S2 Data — Images used in our study. (ZIP) [file pone.0114572.s002.zip › Stimuli/MDS600X800/MDS184.jpg]

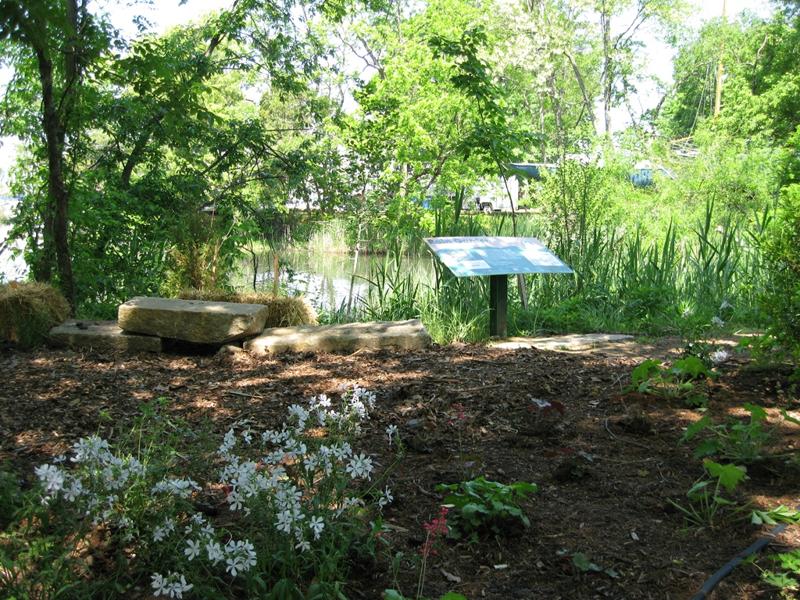

Supplement: S2 Data — Images used in our study. (ZIP) [file pone.0114572.s002.zip › Stimuli/MDS600X800/MDS185.jpg]

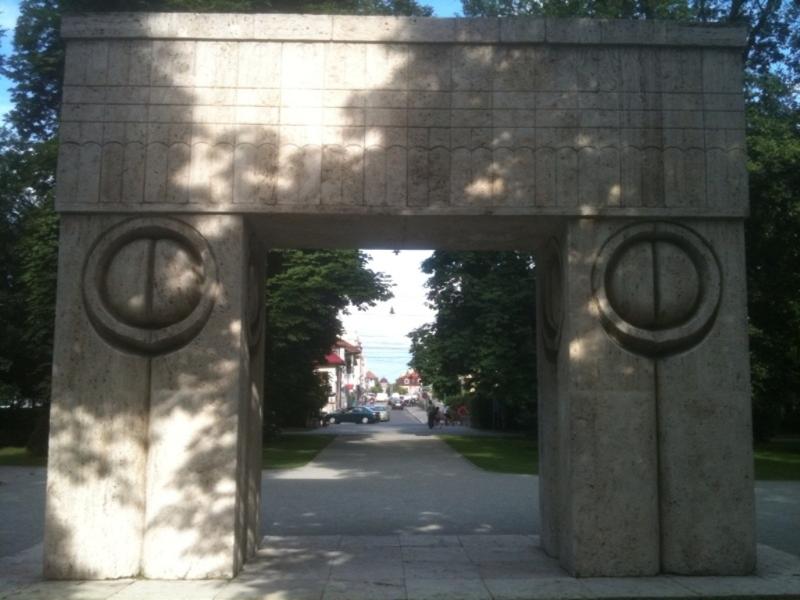

Supplement: S2 Data — Images used in our study. (ZIP) [file pone.0114572.s002.zip › Stimuli/MDS600X800/MDS186.jpg]

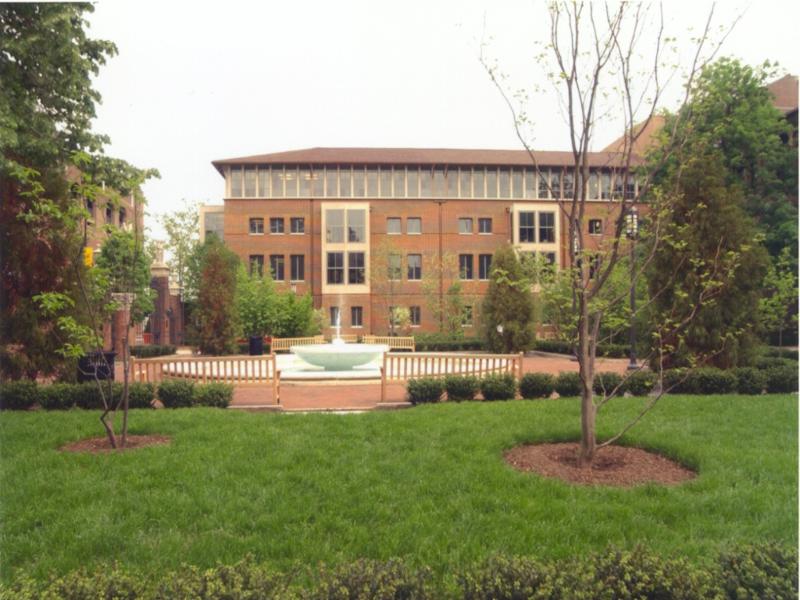

Supplement: S2 Data — Images used in our study. (ZIP) [file pone.0114572.s002.zip › Stimuli/MDS600X800/MDS187.jpg]

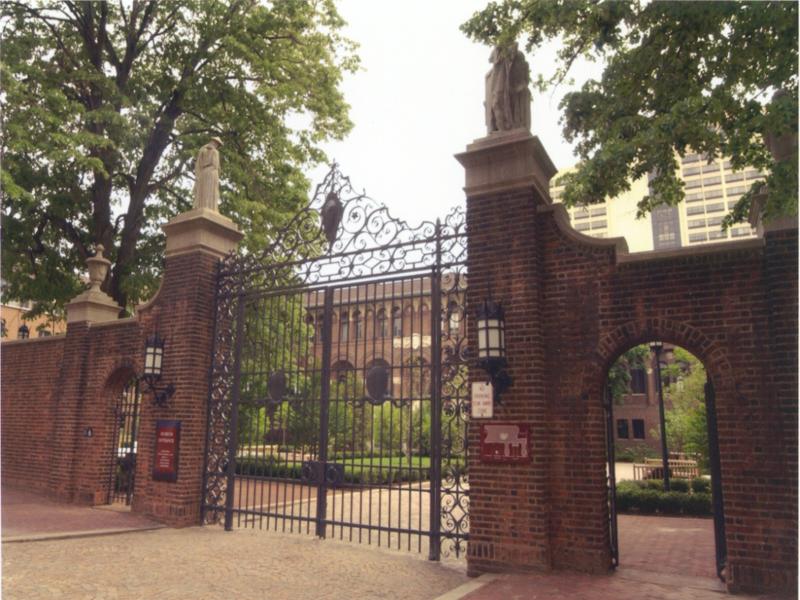

Supplement: S2 Data — Images used in our study. (ZIP) [file pone.0114572.s002.zip › Stimuli/MDS600X800/MDS188.jpg]

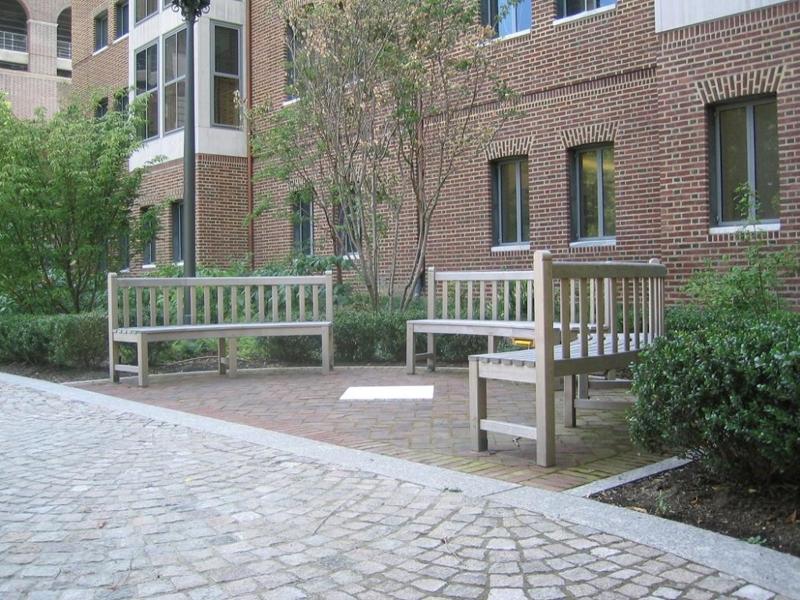

Supplement: S2 Data — Images used in our study. (ZIP) [file pone.0114572.s002.zip › Stimuli/MDS600X800/MDS189.jpg]
